# Loss of ESRP2 Activates TAK1‐MAPK Signaling through the Fetal RNA‐Splicing Program to Promote Hepatocellular Carcinoma Progression

**DOI:** 10.1002/advs.202305653

**Published:** 2023-11-20

**Authors:** Qian Yan, Xiaona Fang, Xiaoxia Liu, Sai Guo, Siqi Chen, Min Luo, Ping Lan, Xin‐Yuan Guan

**Affiliations:** ^1^ Guangdong Provincial Key Laboratory of Colorectal and Pelvic Floor Diseases Guangdong Institute of Gastroenterology The Sixth Affiliated Hospital Sun Yat‐sen University Guangzhou 510655 China; ^2^ Department of General Surgery (Colorectal Surgery) The Sixth Affiliated Hospital Sun Yat‐sen University Guangzhou 510655 China; ^3^ Biomedical Innovation Center, The Sixth Affiliated Hospital, Sun Yat‐sen University Guangzhou 510655 China; ^4^ Sun Yat‐sen University Cancer Center; State Key Laboratory of Oncology in South China; Collaborative Innovation Center for Cancer Medicine Guangzhou 510060 China; ^5^ Department of Pediatric Oncology, Sun Yat‐sen University Cancer Center Guangzhou 510060 China; ^6^ Shenzhen Traditional Chinese Medicine Hospital Shenzhen China; ^7^ Department of Clinical Oncology The University of Hong Kong‐Shenzhen Hospital Shenzhen China; ^8^ Shenzhen Key Laboratory of recurrent metastatic cancer and personalized therapy The University of Hong Kong‐Shenzhen Hospital Shenzhen China; ^9^ State Key Laboratory of Oncology in South China Guangzhou China; ^10^ Department of Clinical Oncology State Key Laboratory for Liver Research The University of Hong Kong Hong Kong China; ^11^ Advanced Energy Science and Technology Guangdong Laboratory Huizhou China; ^12^ MOE Key Laboratory of Tumor Molecular Biology Jinan University Guangzhou China

**Keywords:** epithelial splicing regulatory protein 2, fetal reprogramming, hepatocellular carcinoma, RNA splicing, TAK1/MAPK activation

## Abstract

Tumors usually display fetal‐like characteristics, and many oncofetal proteins have been identified. However, fetal‐like reprogramming of RNA splicing in hepatocellular carcinoma (HCC) is poorly understood. Here, it is demonstrated that the expression of epithelial splicing regulatory protein 2 (ESRP2), an RNA splicing factor, is suppressed in fetal hepatocytes and HCC, in parallel with tumor progression. By combining RNA‐Seq with splicing analysis, it is identified that ESRP2 controls the fetal‐to‐adult switch of multiple splice isoforms in HCC. Functionally, ESRP2 suppressed cell proliferation and migration by specifically switching the alternative splicing (AS) of the TAK1 gene and restraining the expression of the fetal and oncogenic isoform, TAK1_ΔE12. Notably, aberrant TAK1 splicing led to the activation of p38MAPK signaling and predicted poor prognosis in HCC patients. Further investigation revealed that TAK1_ΔE12 protein interacted closely with TAB3 and formed liquid condensation in HCC cells, resulting in p38MAPK activation, enhanced cell migration, and accelerated tumorigenesis. Loss of ESRP2 sensitized HCC cells to TAK1 kinase inhibitor (TAK1i), promoting pyroptotic cell death and CD8+ T cell infiltration. Combining TAK1i with immune checkpoint therapy achieved potent tumor regression in mice. Overall, the findings reveal a previously unexplored onco‐fetal reprogramming of RNA splicing and provide novel therapeutic avenues for HCC.

## Graphical abstract

ESRP2 controls the fetal‐to‐adult switch of multiple splice isoforms in HCC. Loss of ESRP2 leads to splice switching of the TAK1 gene and restrains expression of the fetal and oncogenic isoform, TAK1_ΔE12. Fetal TAK1 interacts closely with TAB3 and forms liquid condensation in HCC cells, resulting in p38MAPK activation, enhanced cell migration, and accelerated tumorigenesis.

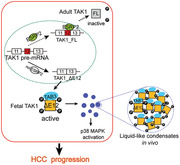

## 1 Introduction

Tumor cells are known to display properties reminiscent of fetal development, contributing to cellular diversity and tumor evolution.^[1^ ^]^ The gene expression signature and key signaling pathways of fetal development are invariably harnessed by tumor cells to facilitate malignant transformation.^[2^ ^]^ Hepatocellular carcinoma (HCC) is the second most prevalent cause of cancer‐associated mortality worldwide, with an inferior prognosis.^[3^ ^]^ Similar to many other tumors, HCCs regain the expression of oncofetal genes that play essential roles in cancer initiation and progression. For example, the oncofetal protein SALL4 is highly expressed in both fetal hepatocytes and HCC cells to drive tumorigenesis.^[4^ ^]^ In addition, an oncofetal tumor microenvironment (TME) comprising shared stromal and immune cell types as well as signaling events in the fetal liver and HCC was identified in a recent study.^[5^ ^]^ Collectively, these studies have determined the oncofetal genes, signaling pathways, and TME that recapitulate early developmental features. However, fetal‐like reprogramming of RNA splicing events in HCC remains to be explored.

We recently established an in vitro hepatocyte differentiation model that mimics liver development and HCC progression.^[6^ ^]^ By comparing the gene expression signatures of fetal hepatocytes and HCC clinical samples, we identified various oncofetal drivers and suppressors, among which epithelial splicing regulatory protein 2 (ESRP2) attracted our attention as a splicing regulatory protein. Previous studies found that ESRP2 controls a conserved epithelial splicing program specifically involved in epithelial‐mesenchymal transition (EMT)‐associated alternative splicing, such as promoting splicing of the epithelial variants of the FGFR2, CTNND1, CD44, and ENAH transcripts.^[^ ^7^, ^8^ ^]^ In addition, ESRP2 has been reported to regulate splicing events of Hippo signaling in hepatocytes and participate in regulating postnatal hepatocytes differentiation,^[9^ ^]^ liver regeneration,^[10^ ^]^ severe alcoholic hepatitis,^[11^ ^]^ and hepatobiliary carcinogenesis in non‐alcoholic fatty liver disease.^[12^ ^]^ All of the evidence indicates that ESRP2 might be critical in development and tumor progression through a remodeling RNA splicing program.

Liquid‐liquid phase separation (LLPS) functions as activation platforms for kinases, offering spatiotemporal control over signaling events. Multiple studies have reported the influence of LLPS on MAPK signaling activation. For instance, cancer‐associated SHP‐2 mutants exhibited LLPS capability, expediting local dephosphorylation and MAPK signaling activation.^[13^ ^]^ Additionally, the fusion protein EML4‐ALK formed phase‐separated condensates, concentrating the RAS activating complex and coordinating the oncogenic RTK/RAS/MAPK pathway.^[14^ ^]^ The convergence of these findings underscores the significance of LLPS as a pivotal mechanism for enhancing kinase signaling activation.

In this study, we determined the fetal‐like recapitulation of RNA splicing events in HCC controlled by ESRP2. Downregulation of ESRP2 was observed in both fetal hepatocytes and HCC and predicted patients’ adverse prognosis. We further verified that ESRP2 inhibited tumor cell proliferation and migration by suppressing the splicing of the oncofetal variant of TAK1 transcripts and inactivating p38MAPK signaling. Notably, the oncofetal TAK1 variant interacted closely with TAB3, forming liquid‐like condensates in HCC cells and sustaining TAK1/MAPK signaling activation. This novel mechanism sheds light on how ESRP2 loss contributes to HCC progression by promoting splicing of variants enriched in the fetal liver. This study provides an unprecedented opportunity to understand oncofetal reprogramming of RNA splicing and its implications in HCC progression.

## 2 Results

### 2.1 ESRP2 is Identified as an Oncofetal Suppressor in HCC

**Figure 1:**
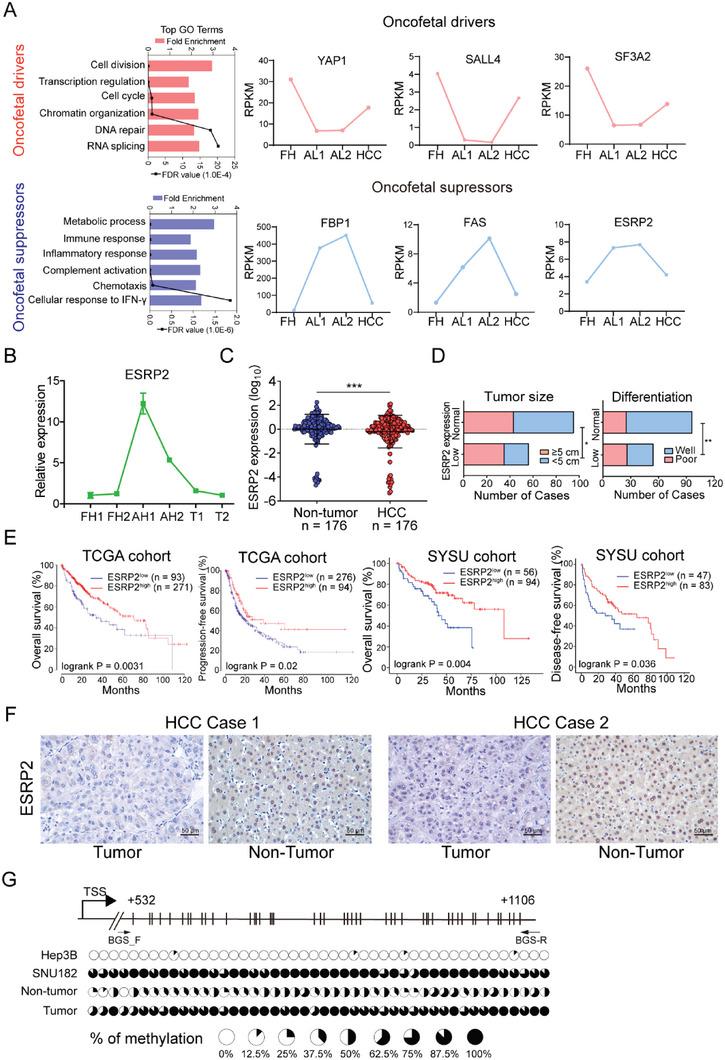
ESRP2 is identified as an oncofetal suppressor and its downregulation predicts poor prognosis of HCC patients. A) Two types of oncofetal genes with typical expression patterns were identified as “oncofetal drivers” or “oncofetal suppressors” based on the RNA‐sequencing data of fetal hepatocyte, adult liver and HCC. Left: Gene ontology enrichment analysis was used to characterize their distinct functions. Right: Expression patterns of representative oncofetal drivers or suppressors. FH, fetal hepatocyte; AL, adult liver; HCC, hepatocellular carcinoma. B) Primary fetal hepatocytes (FH) or adult hepatocytes (AH) were isolated from liver tissues. qRT‐PCR was performed to verify the expression of ESRP2 in FH, AH and HCC tumors. Data were shown as the mean of three independent experiments. C) Scatterplots of the relative expression of ESRP2 detected by qRT‐PCR in HCC and paired adjacent nontumor tissues. Black lines, mean ± SD. D) Low expression of ESRP2 was significantly correlated with large tumor size and poor differentiation status (Pearson's chi‐square tests, ^*^ *p*< 0.05, ^**^ *p*< 0.01). The cutoff value for stratifying HCC patients into 'low' or 'normal' ESRP2 expression subgroups was 0.5. This value was calculated as the fold change in ESRP2 expression in tumor tissues compared with normal tissues. E) Kaplan‐Meier analysis of the OS, DFS or PFS of HCC patients from two independent cohorts stratified by the expression level of ESRP2. For TCGA‐HCC cohort, the Kaplan‐Meier Plotter online tool was used and the best‐performing threshold was selected as the cutoff value. For the SYSU cohort, the same cutoff value as in Figure 1D was utilized. The *P* values of the log‐rank tests were presented. F) IHC staining of ESRP2 in two pairs of non‐tumor tissues and HCC clinical specimens. G) A schematic diagram showing the predicted CpG island around the promoter region of ESRP2. The methylation status of CpG dinucleotides in HCC cells lines and clinical samples was detected by BGS sequencing. The percentage of methylation at each CpG site is displayed in the pie charts.

We recently generated a novel in vitro hepatocyte differentiation model that induces the differentiation of human embryonic stem cells (hESCs) into fetal hepatocytes.^[^ ^6^ ^]^ By comparing the transcriptomic data of fetal hepatocyte (FH), adult liver (AL), and HCC tissues, we can readily identify oncofetal proteins that play essential roles in tumor development. Genes activated or suppressed in fetal hepatocytes and HCC were defined as oncofetal drivers or suppressors, respectively (Figure S1A, Supporting Information). A variety of oncofetal genes have been identified using this model (Table S1, Supporting Information), and Gene Ontology (GO) enrichment analysis revealed that oncofetal drivers were involved in biological processes, including cell division, cell cycle, and RNA splicing, whereas oncofetal suppressors were closely associated with immune response and metabolic processes (**Figure** **1**A). In the present study, we mainly focused on RNA alternative splicing involved in oncofetal regulation. The expression patterns of splicing factors from the oncofetal genes were compared in fetal hepatocytes, adult liver, and HCC. Among them, ESRP2 caught our attention with its distinct expression pattern as an oncofetal suppressor (Figure S1B, Supporting Information). ESRP2 expression was further verified by qRT‐PCR in isolated fetal and adult hepatocytes. The results showed that ESRP2 transcripts were relatively low in fetal hepatocytes, activated in adult hepatocytes, and re‐silenced in HCC (Figure 1B), suggesting that ESRP2 is a novel oncofetal suppressor in HCC.

### 2.2 Loss of ESRP2 Correlates with HCC Progression and Worse Prognosis

To define the clinical significance of ESRP2, we analyzed its expression in HCC tissues from TCGA database. ESRP2 expression was downregulated in tumor tissues compared with non‐tumor counterparts (Figure S1C, Supporting Information) and decreased in middle/late‐stage and poorly differentiated tumors compared with early stage and well‐differentiated ones, respectively (Figure S1D, Supporting Information). This result was reinforced by quantification of ESRP2 mRNA in 176 pairs of HCC samples. Downregulation of ESRP2 was frequently observed in HCC specimens (Figure 1C) and was significantly correlated with larger tumor size (Pearson χ^2^ test, *P* < 0.05) and poor differentiation status (Pearson χ^2^ test, *P* < 0.01) (Figure 1D). More importantly, Kaplan‐Meier analysis revealed that patients with low ESRP2 expression displayed worse overall survival (OS; *P* < 0.01), progression‐free survival (PFS; *P* < 0.05), and disease‐free survival (DFS; *P* < 0.05) in both the TCGA cohort and an independent validation cohort (Figure 1E). The protein expression level of ESRP2 was also determined by IHC staining in paired clinical samples, and a relatively strong ESRP2 expression was observed in non‐tumor liver tissues (Figure 1F). Expression of ESRP2 in HCC cell lines was detected using qRT‐PCR and western blot analysis. Compared with the immortalized liver cell line MIHA, downregulation of ESRP2 was detected in 4/5 HCC cell lines (Figure S1E,F, Supporting Information). Collectively, these results strongly indicate that low expression of ESRP2 is closely associated with HCC progression and that ESRP2 is a potential prognostic biomarker for HCC patients.

### 2.3 Promoter Hypermethylation of ESRP2 in HCC

Oncofetal suppressors are usually turned off in tumor tissues, but the underlying mechanism of their inactivation remains elusive. Analysis of the genetic alteration of ESRP2 in HCCs from TCGA database suggested that DNA variations of ESRP2 are rare (Figure S1G, Supporting Information). Interestingly, a negative correlation between ESRP2 mRNA expression and DNA methylation levels was observed in the TCGA‐HCC cohort (Pearson R = −0.34; *P* < 0.001) (Figure S1H, Supporting Information). The inverse correlation was further confirmed in pan‐cancer and HCC cell lines from the Cancer Cell Line Encyclopedia (CCLE) dataset (Figure S1I, Supporting Information). The enzymes governing DNA methylation regulation encompass DNA methyltransferases (DNMTs), which aid in methylation establishment, as well as the Ten‐Eleven Translocation (TET) enzymes accountable for active demethylation. We then investigated the expression correlation between ESRP2 and DNMTs or TETs enzymes in the TCGA‐HCC cohort. The expression of ESRP2 was found positively correlated with TET1, TET2, and TET3 (Figure S1J, Supporting Information). To validate the effect of hypermethylation on ESRP2 silencing, bisulfite genomic sequencing within the CpG island (+532 to +1106) around the promoter region was conducted in HCC cell lines with different expression level of ESRP2, as well as in one pair of clinical samples. Methylation was rarely detected in ESRP2‐expressing Hep3B cells, whereas a high density of methylation was observed in ESRP2‐absent SNU‐182 cells (Figure 1G). Consistently, HCC specimens with ESRP2 silencing showed higher methylation density compared to non‐tumor tissues (Figure 1G), supporting that promoter hypermethylation might be the major cause of ESRP2 downregulation in HCC.

### 2.4 Tumor‐Suppressive Function of ESRP2 in HCC

**Figure 2:**
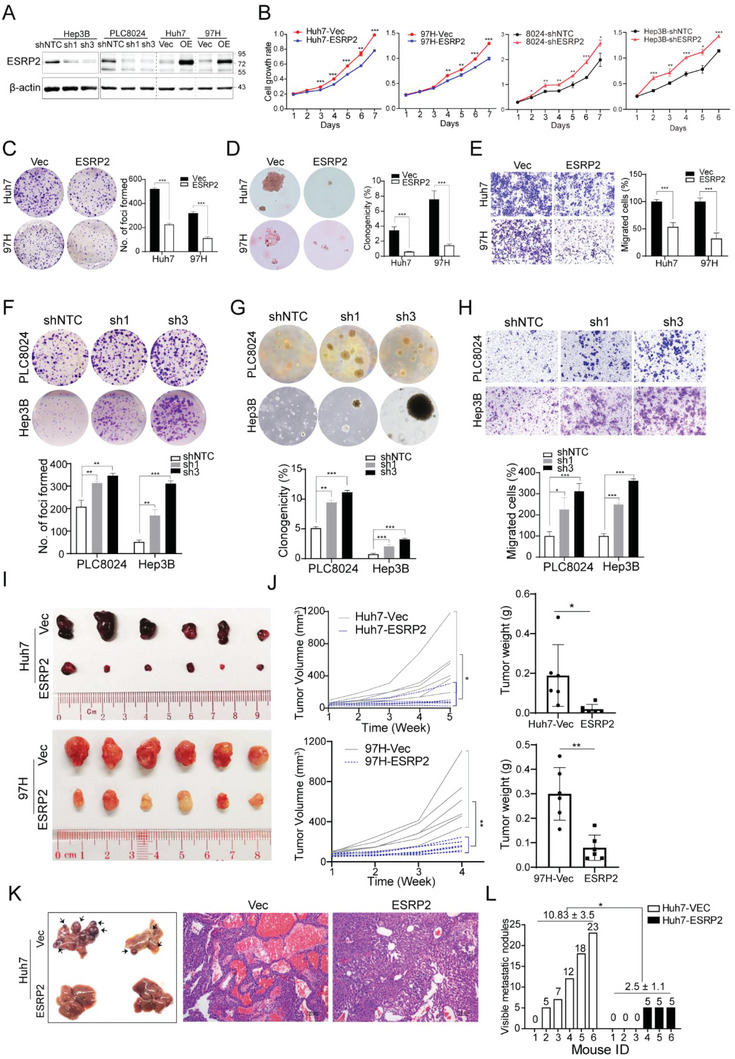
ESRP2 exerts tumor‐suppressive function in HCC. A) Western blot analysis was used to detect ESRP2 expression in ESRP2‐transefeced or silenced HCC cells. β‐actin was used as loading control. B) XTT assay was used to determine the HCC cells proliferation rates. C) Representative images of foci formation in monolayer culture. The numbers of foci were calculated and depicted in the bar chart. D) Representative images of colony formation in soft agar. The clonogenicity ratio was illustrated in the bar chart. E) Transwell migration assay was used to compare cell motility between Vec‐ and ESRP2‐transfected Huh7 and 97H cells. Representative images of migration were shown in the left panel. The number of migrated cells was calculated and depicted in the bar chart. F–H) Representative images of foci formation (F), colony formation in soft agar (G), and cell migration (H) induced by PLC8024‐shNTC/shESRP2 and Hep3B‐shNTC/shESRP2 (upper panel). The numbers of foci, colonies, and migrated cells were illustrated in the bar chart, respectively (lower panel). I,J) Images of xenograft tumors induced by subcutaneous injection of indicated cells into nude mice (I). Tumor growth curves were summarized in the line chart, and the average tumor weight was expressed as the mean ± SD of 6 mice (J). K) Representative images of livers derived from nude mice after intrasplenic injection of Vec‐ and ESRP2‐transfected Huh7 cells (left). Representative images of hematoxylin and eosin staining of liver sections mentioned above (right). L) The numbers of metastatic nodules on the surface of the liver were summarized and denoted on the bar chart. Statistics: in Figure 2B–L, Student's *t*‐test was used for statistical analysis, ^*^ *p*< 0.05, ^**^ *p*< 0.01, ^***^ *p*< .001, data were shown as mean ± SD. Data represent at least three independent experiments.

To explore the tumor‐suppressive role of ESRP2 in HCC, gain‐ and loss‐of‐function studies were performed. HCC cell lines with ESRP2 overexpression or silencing were generated, and the expression of ESRP2 was determined by western blotting (**Figure** **2**A). Compared to control cells, ESRP2‐transfected cells showed impaired proliferation (Figure 2B), foci formation (Figure 2C), colony formation in soft agar (Figure 2D) and migration (Figure 2E). Conversely, knockdown of ESRP2 significantly enhanced proliferation, foci formation, colony formation in soft agar, and migratory capabilities of HCC cells (Figure 2B,F–H). To assess the in vivo tumor‐suppressive function of ESRP2, we subcutaneously injected HCC cells transfected with Vec or ESRP2, as well as shNTC or shESRP2, into the left and right dorsal flanks of nude mice. Notably, tumors induced by ESRP2‐transfected cells showed smaller volumes and decreased tumor weights compared to tumors induced by vector‐transfected cells (Figure 2I,J). Conversely, silencing of ESRP2 led to an elevated tumorigenesis ratio and increased tumor weights relative to the control group (Figure S2A,B, Supporting Information). Furthermore, the effect of ESRP2 on metastasis was investigated by the intrasplenic injection of HCC cells into nude mice. Liver metastasis was observed and confirmed by histological analysis (Figure 2K). The number of metastatic nodules formed on the liver surface was significantly lower in mice injected with Huh7‐ESRP2 cells (10.83 ± 3.5) than in mice injected with Huh7‐Vec cells (2.5 ± 1.1; *P* < 0.05, Student's *t* test, Figure 2L). Collectively, these data indicate that ESRP2 suppresses HCC growth and metastasis, both in vitro and in vivo.

### 2.5 ESRP2 Suppresses the Fetal Splicing Program in HCC

**Figure 3:**
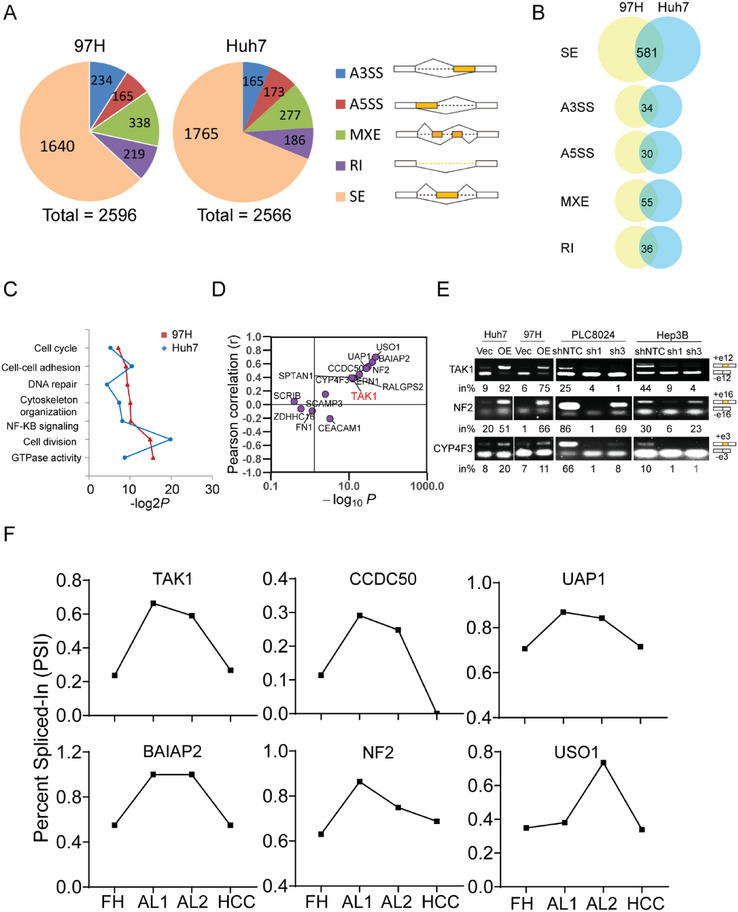
Global profiles of ESRP2‐affected AS events in HCC. A) ESRP2‐affected AS events in 97H (left) and Huh7 (right) cell lines. The AS events were classified into 5 categories: skipped exon (SE), retained intron (RI), alternative 5′ splice site (A5SS), alternative 3′ splice site (A3SS), and mutually exclusive exon (MXE). The cutoff settled for differential alternative splicing events in Figure 3A–C was *p*< 0.05. B) Overlapping AS events in each category between 97H and Huh7 cell lines. C) Gene ontology of the ESRP2‐affected AS targets shared by 97H and Huh7 cells. Fisher's exact *P* values (–log_2_ transformed) were plotted for each enriched functional category. D) Pearson correlation analysis between the Percent Spliced In (PSI) indexes of each AS target with ESRP2 expression in TCGA‐HCC cohort. Pearson coefficient R was used to denote the correlation. Every dot represents one AS target of ESRP2. E) RT‐PCR validation of ESRP2‐affected AS events. The structure of each PCR product was indicated schematically on the right. Alternative exons affected by ESRP2 were painted in orange. The percentage of exon inclusion products (in%) out of the total products was indicated below each gel. F) The PSI index of ESRP2‐affected splicing events was analyzed using transcriptomic data from fetal hepatocytes (FH), adult liver (AL) and HCC samples.

To screen for ESRP2‐regulated AS events involved in HCC progression, we performed high‐throughput RNA sequencing on Vec‐ and ESRP2‐ transfected HCC cell lines 97H and Huh7. A total of 2596 and 2566 ESRP2‐reguated AS events (*P* < 0.05, Likelihood‐Ratio test) were identified in 97H and Huh7 cells, respectively, which could be divided into five categories, with the largest number being skipped exon (SE) events (**Figure** **3**A; Table S2, Supporting Information). Multiple AS events were shared by 97H and Huh7 cells, the majority of which belonged to the SE category (Figure 3B). Interestingly, these overlapping ESRP2‐regulated splicing targets were enriched in functional clusters, such as cell cycle, cell adhesion, DNA repair, and cytoskeleton organization (Figure 3C), indicating that they play important roles in tumor cell proliferation and migration.

Among all the ESRP2‐regulated AS events, we mainly focused on the 50 shared SE events by 97H and Huh7 cells with FDR values less than 0.05 (Table S3, Supporting Information). To further identify splicing events that play essential roles in HCC progression, we downloaded the Percent Spliced In (PSI) indexes of each SE events from TCGA SpliceSeq,^[15^ ^]^ a web‐based resource for AS quantification in cancer, and analyzed their clinicopathological association and prognostic value in HCC. A total of 15 SE events were found to be crucial for HCC progression, as their PSI was significantly different between tumor and normal tissues and proved to be significant prognostic factors for OS in patients with HCC (Figure S2C,D and Table S4, Supporting Information). Subsequent analysis indicated the regulatory role of ESRP2 in most SE events, as evidenced by a high correlation between ESRP2 expression and PSI indexes in the TCGA‐HCC cohort (Figure 3D). To verify the accuracy of our RNA‐Seq results on AS, we subsequently validated the crucial AS events affected by ESRP2 in four HCC cells lines. Representative results of validated AS events confirmed that ESRP2 regulated the splicing the target exons (Figure 3E; Figure S2E, Supporting Information).

Variants that showed upregulation or suppressed in both fetal hepatocytes and HCC were defined as fetal variants or adult variants, respectively. To better understand the regulatory role of ESRP2 on fetal/adult variants expression, we further investigated the PSI value of ESRP2‐regulated AS events using RNA‐Seq data from fetal hepatocytes, adult liver, and HCC samples. Strikingly, in line with the expression pattern of ESRP2, the PSI value of multiple splicing targets were found to be down‐regulated in both fetal hepatocytes and HCC (Figure 3F), suggesting that ESRP2 may inhibit the accumulation of fetal splicing variants in HCC.

### 2.6 ESRP2 Suppresses TAK1 exon 12 Skipping and Inactivates p38MAPK Signaling

**Figure 4:**
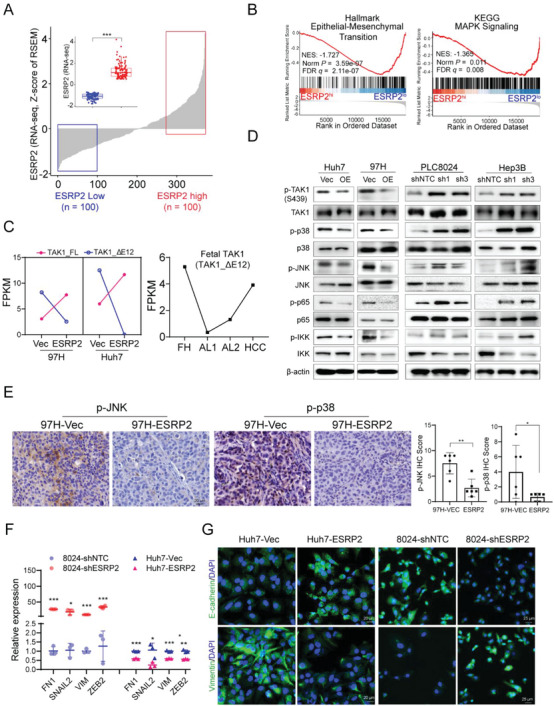
ESRP2 mediates inclusion of exon 12 in TAK1 pre‐mRNA and inactivates p38MAPK signaling. A) ESRP2 expression in HCC specimens (n = 371) based on TCGA datasets. Inset shows comparison between low (blue box, n = 100) and high (red box, n = 100) ESRP2 expression cohorts. RSEM, RNA‐Seq by Expectation Maximization. B) Representative gene sets enriched by GSEA analysis of DEGs from HCC specimens with low or high ESRP2 expression in the TCGA cohort. C) Left: FPKM expression values of TAK1 variants based on RNA‐Seq data from Vec‐ or ESRP2‐ transfected HCC cells; Right: FPKM expression values of the fetal TAK1 variant analyzed using transcriptomic data from fetal hepatocytes (FH), adult liver (AL) and HCC samples. D) Western blot analysis of phosphorylation of TAK1, p38, JNK, p65, IKK in ESRP2‐overexpressed or silenced HCC cells. E) Representative images of IHC staining for p‐JNK and p‐p38 in xenograft tumors induced by Vec‐ or ESRP2‐ transfected HCC cells. IHC scores were calculated and shown in the bar chart, with each dot representing an individual xenograft tumor. F) qRT‐PCR was used to assess the relative expression of EMT markers in ESRP2‐overexpressed or silenced HCC cells. G) Immunofluorescence staining of E‐cadherin and Vimentin in ESRP2‐overexpressed or silenced HCC cells. Cell nuclei were counterstained with DAPI (blue). Scale bars: 20 µm.

To identify the key signaling pathways that may be regulated by ESRP2, we conducted gene set enrichment analysis (GSEA) on TCGA‐HCC gene expression data and examined whether high or low ESRP2 expression was associated with particular gene sets (**Figure** **4**A). GSEA analysis showed that tumors with low ESRP2 expression exhibited enrichment of genes associated with EMT and MAPK signaling (Figure 4B), suggesting that ESRP2 may be involved in controlling the MAPK pathway to affect HCC progression. Among the screened AS targets closely related to HCC progression, TGF‐β‐activated kinase 1 (TAK1, also known as MAP3K7) attracted our attention because of its potential role in the regulating of p38MAPK signaling.^[16^ ^]^ Human TAK1 has 17 exons, of which exons 12 and 16 are subject to AS regulation, giving rise to four different isoforms depending on the inclusion/exclusion of the two alternative exons.^[17^ ^]^ Splicing variants switching from exon 12 exclusion (TAK1_ΔE12) to inclusion (TAK1_FL) were observed in our RNA‐Seq data after ESRP2 transfection (Figure S3A,B, Supporting Information). The expression levels of TAK1_ΔE12 decreased while TAK1_FL increased in ESRP2‐transfected HCC cell lines (Figure 4C). Interestingly, TAK1_ΔE12, a fetal TAK1 variant, was found to be highly expressed in fetal hepatocytes, nearly absent in adult hepatocytes, but reactivated in HCC (Figure 4C), suggesting its role as an oncofetal protein in the development and progression of HCC. RT‐PCR was used to determine the expression of these two variants in the HCC cell lines. The results showed that ESRP2 overexpression significantly suppressed TAK1 exon 12 skipping, while knockdown of ESRP2 facilitated the expression of the TAK1_ΔE12 isoform (Figure 3E), suggesting that the inclusion of TAK1 exon 12 is ESRP2 dependent.

It has been reported that TAK1 functions as a key kinase to activate p38MAPK, JNK and NF‐κB signaling upon phosphorylation.^[17^ ^]^ To investigate whether these signaling pathways are influenced by ESRP2, we performed western blot analysis of HCC cells overexpressing or silencing ESRP2. Phosphorylation of TAK1 at Ser439 was upregulated upon ESRP2 knockdown, which coincided with the activation of downstream p38MAPK, JNK, and NF‐κB signaling. Conversely, the phosphorylation of TAK1, p38, JNK, p65, and IKK was decreased in HCC cells with ESRP2 ectopic expression (Figure 4D). IHC staining of p‐p38 and p‐JNK was also performed in xenograft tumors originating from ESRP2‐overexpressed or silenced HCC cells. The findings demonstrated reduced expression of p‐JNK and p‐p38 in ESRP2‐overexpressed tumors, whereas their levels were elevated in ESRP2‐silenced tumors (Figure 4E, Figure S3C, Supporting Information), indicating that ESRP2 might suppress TAK1‐mediated signaling by regulating the AS of TAK1. Furthermore, downstream target genes of the MAPK pathway, including EMT markers FN1, SNAIL2, VIM, ZEB2, and E‐cadherin, were assessed using qRT‐PCR or immunofluorescence staining in ESRP2‐overexpressed or silenced HCC cells. The findings revealed that ESRP2 could enhance E‐cadherin expression while suppressing the expression of Vimentin, FN1, SNIAL2, and ZEB2 in HCC cells (Figure 4F,G)

The clinicopathological significance of TAK1 exon 12 splicing was investigated in the TCGA‐HCC cohort. The PSI of TAK1 exon 12 was significantly lower in HCC tissues than in normal liver tissues (Figure S2C, Supporting Information) and decreased in parallel with tumor grade progression (Figure S3D, Supporting Information). The exclusion of TAK1 exon 12 (low PSI indices) was significantly correlated with reduced ESRP2 expression in patients with HCC (Figure S3E, Supporting Information). In addition, patients with a low PSI of TAK1 exon 12 had a worse overall survival probability (Figure S2D, Supporting Information). GSEA analysis was performed to investigate the signaling pathways influenced by TAK1 exon 12 splicing, and the results showed that tumors with low PSI were enriched in EMT, MAPK, and primary immunodeficiency signaling (Figure S3F, Supporting Information). Taken together, these data imply that ESRP2 suppresses fetal TAK1 variant expression, leading to the inactivation of p38MAPK in HCC.

### 2.7 Fetal TAK1 is Constitutively Activated and Forms Liquid‐Like Condensates with TAB3 in HCC Cells

**Figure 5:**
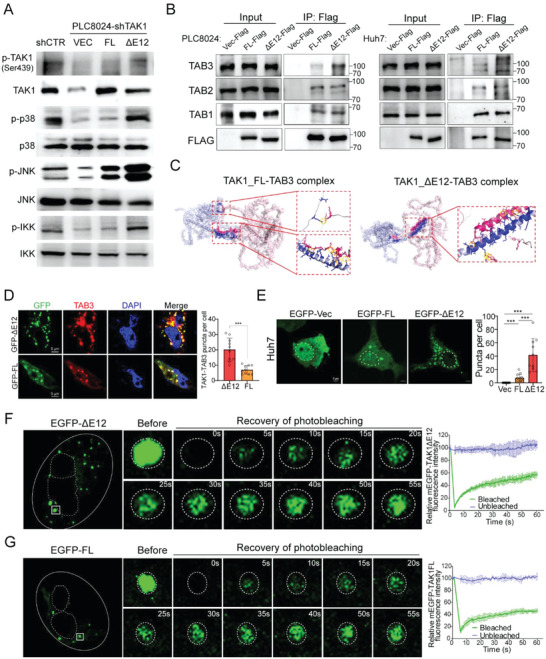
TAK1_ΔE12 is constitutively activated and forms liquid condensates with TAB3 within HCC cells. A) Effects of TAK1_FL and TAK1_ΔE12 isoforms on the activation of TAK1, p38, JNK, and IKK in HCC cells. HCC cells were stably transfected with shCTR (scrambled shRNA) or shTAK1, then TAK1_ΔE12 or TAK1_FL was introduced into shTAK1 cells. The levels of phosphor‐ or total‐TAK1, p38, JNK, and IKK in cell lysates were analyzed by western blot. B) PLC8024 or Huh7 cells were transfected with Flag‐tagged empty vector, TAK1_FL or TAK1_ΔE12. Cell lysates were immunoprecipitated with anti‐Flag and normal IgG. Western blot analysis was performed to detect TAB1, TAB2, and TAB3 in this immunoprecipitation. C) Predicted structure of human TAK1_FL‐TAB3 complex (left) and TAK1_ΔE12‐TBA3 complex (right). The binding domain was colored blue and red. The figures on the right displayed enlarged views of the binding domain structure, with important interacting residue pairs shown in stick form and hydrogen bonds encircled in yellow. D) Huh7 cells were transfected with GFP‐tagged TAK1_FL or TAK1_ΔE12, followed by immunofluorescence staining with TAB3 antibody (red). Nuclei were counterstained with DAPI (blue). Quantitative result of the co‐localization puncta between TAK1 and TAB3 was summarized in the bar chart. E) Left: live cell imaging of GFP‐tagged TAK1_FL or TAK1_ΔE12 in Huh7 cells; Right: quantitative analysis of the puncta per cell (10 cells per sample). F,G) Left: Representative images of the FRAP assay with EGFP‐TAK1_ΔE12 (F) or EGFP‐TAK1_FL (G) transfected Huh7 cells. The white box highlighted the puncta undergoing targeted bleaching; Right: Quantification of FRAP data for EGFP‐TAK1 puncta over a 60 s time course. Data were plotted as means ± SD (n = 4). Statistics: in Figure 4D,E, Student's *t*‐test was used for statistical analysis, ^***^ *p*< .001, data were shown as mean ± SD. Data represent at least three independent experiments.

To decipher the oncogenic role of the fetal TAK1 variant and the underlying mechanism, we depleted endogenous TAK1 expression in HCC cells with shRNA targeting both variants (shTAK1 cells) and then stably transfected TAK1_ΔE12 (fetal variant) and TAK1_FL (adult variant) into shTAK1 cells. Western blot analysis showed that fetal TAK1 was constitutively activated, marked by upregulated phosphorylation at Ser439 compared with adult TAK1. Accordingly, the phosphorylation levels of p38MAPK, JNK, p65, and IKK were decreased upon TAK1 silencing and restored by ectopic expression of fetal TAK1 instead of adult TAK1 (**Figure** **5**A). Since activation of TAK1 requires the assembly of kinase complexes with TRAF6 and TABs,^[18^ ^]^ the interactions between TABs and TAK1 variants were investigated. Immunoprecipitation analysis demonstrated that fetal TAK1 with exon 12 skipping displayed a stronger ability to bind to TAB3 than adult TAK1, although binding to TAB1 or TAB2 was not affected by the presence or absence of exon 12 (Figure 5B). To unravel the mechanism underlying exon 12 splicing in TAK1‐TAB3 interaction, we modeled the protein structures of TAK1_FL, TAK1_ΔE12, and TAB3. The results showed that the binding affinities of TAB3‐TAK1_FL and TAB3‐TAK1_ΔE12 are −10.5 and −12.8 kcal mol^−1^, respectively (see revised Table S5, Supporting Information), indicating that exon 12 skipping in fetal TAK1 promotes the formation of TAB3‐TAK1 complex. Notably, the TAB3‐TAK1_ΔE12 complex exhibited a greater number of contact points among interacting residues in contrast to the TAB3‐TAK1_FL complex (see revised Table S6, Supporting Information). A comprehensive analysis of the binding structure unveiled shorter distances between hydrogen bonds within the TAB3‐TAK1_ΔE12 complex (averaging 2.64 Å compared to 1.72 Å). Moreover, the interacting residues in the TAB3‐TAK1_ΔE12 complex are clustered along two parallel‐aligned α‐helices, suggesting higher potential stability compared to the TAB3‐TAK1_FL complex (see revised Figure 5C).

Immunofluorescence staining further verified the colocalization of exogenous TAK1 and endogenous TAB3 in the cytoplasm of HCC cells. Notably, discrete puncta staining patterns were observed for the TAK1‐TAB3 complex, with significantly more colocalized speckles for fetal TAK1 and TAB3 than for adult TAK1 and TAB3 (Figure 5D). Discrete puncta are always visualized when proteins form condensates via multivalent interactions, providing membrane‐free compartments to concentrate intracellular biochemical reactions,^[19^ ^]^ we therefore investigated whether TAK1‐TAB3 forms condensates in the cytoplasm of HCC cells. The amino acid sequences of TAK1 and TAB3 were analyzed to predict intrinsically disordered regions (IDRs), which are known to facilitate condensates formation.^[20^ ^]^ The results showed that both TAB3 and fetal TAK1 contained extended IDRs; however, the IDR of adult TAK1 was interrupted by exon 12 inclusion (Figure S4A,B, Supporting Information). To determine whether puncta occur in live cells, we transfected HCC cells with EGFP‐labeled fetal or adult TAK1. Live‐cell imaging showed that fetal TAK1 cells formed more puncta in the cytoplasm than adult TAK1 cells (Figure 5E). Immunofluorescence staining of TAK1 was then performed in Vec‐ or ESRP2‐transfected HCC cells to prevent specific condensate formation resulting from TAK1 overexpression. The findings indicated that Vec‐transfected cells exhibited more puncta‐staining in comparison to ESRP2‐transfected cells (Figure S4C, Supporting Information). The properties of these puncta were investigated using fluorescence recovery after photobleaching (FRAP) experiments. Both fetal and adult TAK1 in the foci achieved half‐maximal recovery within 1 min after photobleaching (Figure 5F,G), reflecting the dynamic reorganization and liquid‐like properties of these puncta in vivo. Taken together, these results suggest that fetal TAK1 interacts closely with TAB3, and the complex may form liquid condensates in HCC cells, leading to continuous activation of downstream signaling.

### 2.8 Fetal TAK1 Splicing Variant Increases the Oncogenic Capacities of HCC Cells

**Figure 6:**
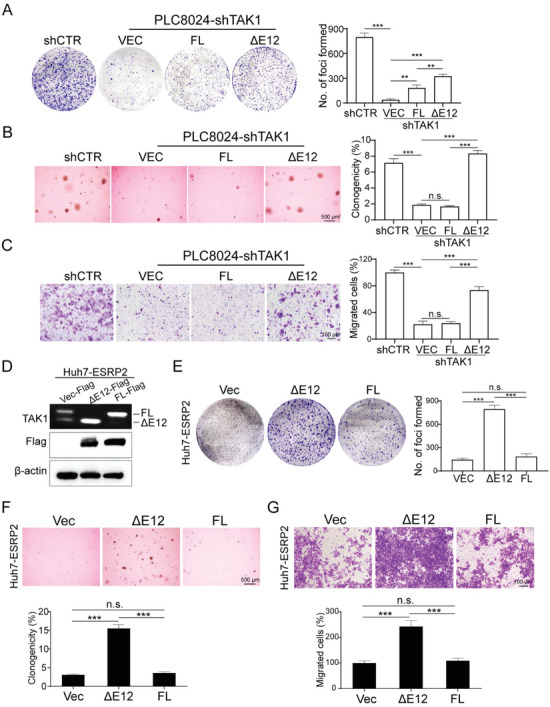
TAK1 isoforms differ in their biological functions. A–C) TAK1 was knocked down by the specific shRNA in PLC8024 cells, and the TAK1_FL or TAK1_ΔE12 isoforms were overexpressed individually. Representative images of foci formation (A), colony formation in soft agar (B), and cell migration (C) induced by indicated cells were shown in the left panel. The numbers of foci, colonies, and migrated cells were illustrated in the bar chart, respectively (right panel). D) Flag‐tagged TAK1_FL or TAK1_ΔE12 were transfected into ESRP2‐overepxressed Huh7 cells. RT‐PCR and western blot were used to validate the isoforms expression. β‐actin was used as loading control. E–G) Representative images of foci formation (E), colony formation in soft agar (F), and cell migration (G) induced by indicated cells. The numbers of foci, colonies, and migrated cells were demonstrated in the bar chart, respectively. Statistics: In Figure 6A–G, data were shown as mean ± SD, and student's *t*‐test was used for statistical analysis, ^**^ *p*< 0.01, ^***^ *p*< .001, n.s., nonsignificant. Data represent at least three independent experiments.

Given that ESRP2 depletion facilitated the expression of fetal TAK1 (TAK1_ΔE12 with exon 12 skipping), we investigated how fetal TAK1 contributes to HCC malignant progression. We designed shRNA targeting endogenous TAK1 in HCC cells and then introduced fetal or adult TAK1 variants into TAK1 knockdown cells, respectively. Functional studies revealed that TAK1 knockdown suppressed tumor cell foci formation (**Figure** **6**A), colony formation in soft agar (Figure 6B), and migratory capabilities (Figure 6C), which could be recovered by ectopic expression of fetal, but not adult, TAK1. Additionally, to examine whether TAK1 variants could rescue the tumor‐suppressive function of ESRP2, we introduced both variants into ESRP2‐overexpresed HCC cells. The specific expression of each variant was verified using RT‐PCR (Figure 6D). Functional assays, including foci formation (Figure 6E), colony formation in soft agar (Figure 6F), and transwell migration (Figure 6G), were then performed, and the results showed that fetal TAK1 significantly rescued ESRP2‐suppressed tumor proliferation and motility, whereas adult TAK1 failed to do so.

### 2.9 ESRP2 Downregulation Sensitizes HCC cells to TAK1 Inhibitors

**Figure 7:**
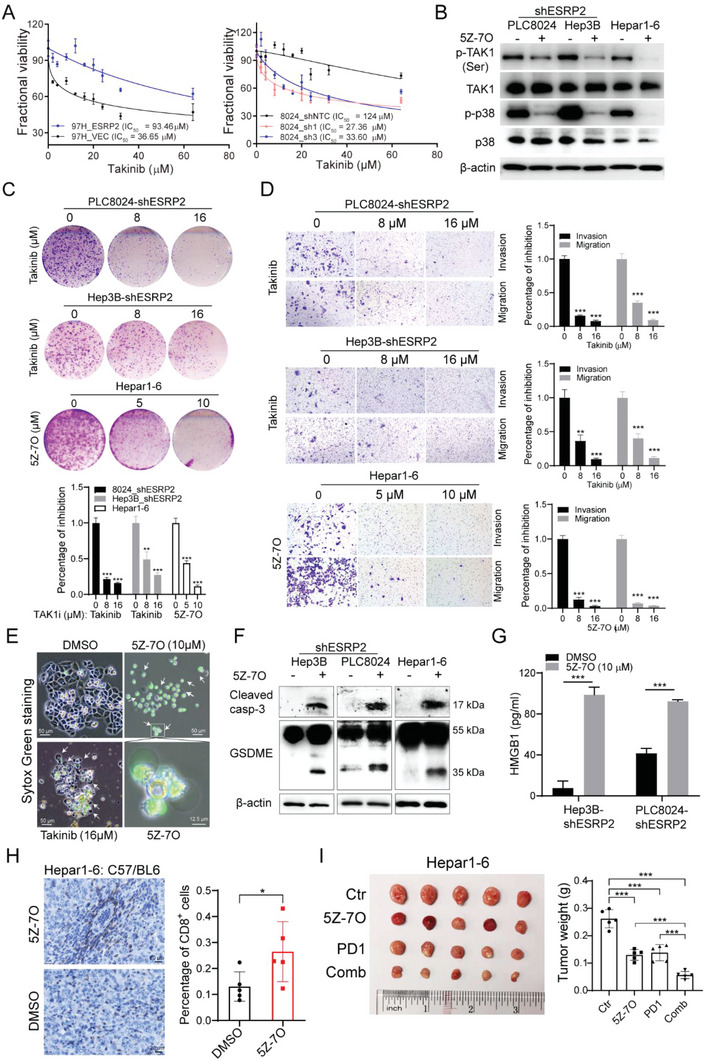
Targeting TAK1 might be a novel effective strategy in ESRP2‐deficient HCC treatment. A) IC_50_ of ESRP2‐overepxressed or silenced HCC cells was determined by XTT assay after treatment with Takinib at indicated concentrations for 48 h. Data were represented as means ± SD. B) Western blot analysis was used to detect the phosphorylation or total level of TAK1 and p38 in indicated cells after treatment with 5 µmol 5Z‐7‐oxozeaenol for 12 h. C) Representative images of foci formation of PLC8024/Hep3B‐shERRP2 cells and Hepar1‐6 cells following 14 days of exposure to indicated concentrations of Takinib or 5Z‐7‐oxozeaenol. The percentage of cell growth inhibition was depicted in the bar chart. D) Representative images of cell migration following treatment of indicated concentrations of Takinib or 5Z‐7‐oxozeaenol for 3 days. The percentage of cell migration inhibition was depicted in the bar chart. E) PLC8024 cells were treated with indicated concentrations of Takinib or 5Z‐7‐oxozeaenol for 24 h. Add 1 µM SYTOX^®^ Green stain (Invitrogen) into the culture medium and incubate for 15 min. Dead Cells stained with SYTOX green dye were viewed with a fluorescence microscope under 488 nm excitation. F) Cleaved Caspase‐3 and GSDME were analyzed by western blot in 5Z‐7‐oxozeaenol treated HCC cells. β‐actin was used as loading control. G) ELISA assay was used to detect the release of HMGB1 in the culture supernatant of 5Z‐7‐oxozeaenol‐treated Hep3B/PLC8024‐shESRP2 cells. H) Mice with established subcutaneous Hepar1‐6 tumors of similar size were randomly divided into two groups and were given vehicle control or 15 mg kg^−1^ 5Z‐7‐oxozeaenol every other day for 12 days. Infiltration of CD8+ T cells into xenograft tumors was evaluated by immunohistochemistry (left) and quantified with Image J (right). Data for each tumor (n = 5) were shown as mean ± SD. I) Mice with established subcutaneous Hepar1‐6 tumors of similar size were randomly divided into four groups and were given vehicle control, 15 mg kg^−1^ 5Z‐7‐oxozeaenol, 5 mg kg^−1^ anti‐PD1 antibody, or combined treatment. 5Z‐7‐oxozeaenol was given every other day and PD1 antibody was administered every three days via intraperitoneal injection. The image of tumors (left) and a bar graph (right) showing the weight of tumors at the end of treatment. Each dot represents a single tumor. Statistics: in Figure 7A–I, student's *t*‐test was used for statistical analysis, ^*^ *p*< 0.05, ^**^ *p*< 0.01, ^***^ *p*< .001, data were shown as mean ± SD. Data represent at least three independent experiments.

Since HCCs with ESRP2 downregulation might be highly dependent on TAK1‐mediated signaling for proliferation and migration, we sought to determine whether they are sensitive to TAK1 inhibitors. The effect of takinib, a small molecule inhibitor of TAK1,^[21^ ^]^ as well as 5Z‐7‐oxozeaenol, a natural compound that acts as a potent TAK1 inhibitor,^[22^ ^]^ was tested in several human and murine HCC cell lines. Viability analysis showed that ESRP2‐silenced PLC8024 and Hep3B cells and ESRP2‐low‐expressing Hepar1‐6 were highly sensitive to TAK1 inhibition (Figure S5A,B, Supporting Information). Additionally, the IC50 of Takinib was significantly increased upon ESRP2 ectopic expression, but decreased as a result of ESRP2 knockdown (**Figure** **7**A), indicating that ESRP2 deficiency sensitizes HCC cells to TAK1 inhibition. Western blot analysis was then performed to examine the cellular signaling altered by the TAK1 inhibitor, and the results demonstrated that 5Z‐7‐oxozeaenol significantly suppressed the phosphorylation of TAK1 and p38, but did not affect total protein levels (Figure 7B). Functional studies were performed in ESRP2‐silenced or low‐expression HCC cells following TAK1 inhibitor treatment. Both Takinib and 5Z‐7‐oxozeaenol inhibited tumor cell foci formation (Figure 7C), migration, and invasion (Figure 7D) in a dose‐dependent manner.

Previous studies have shown that TAK1 deficiency or pathogen blockade induces macrophage pyroptosis in a RIPK1‐dependent or independent manner.^[^ ^23^, ^24^, ^25^ ^]^ We then investigated whether TAK1 inhibition causes pyroptotic cell death in HCC cells. Treatment of HCC cells with takinib or 5Z‐7‐oxozeaenol in vitro increased cell death as determined by Sytox Green staining compared with DMSO‐treated cells (Figure 7E). Morphologically, the dying cells exhibit cell swelling and large bubbles emerging from the plasma membrane, which are typical features of pyroptosis.^[26^ ^]^ As pyroptosis can be triggered by gasdermin family members owing to cleavage,^[26^ ^]^ we examined the processing of gasdermin D or E (GSDMD or GSDME), two typical executors of pyroptosis, in human or murine HCC cell lines. Treatment with 5Z‐7‐oxozeaenol caused the production of a 35 kDa GSDME cleavage fragment in HCC cells (Figure 7F); however, no cleavage of GSDMD was observed (data not shown). Since GSDME has been shown to initiate pyroptosis via cleavage by caspase‐3,^[27^ ^]^ we determined the expression of caspase‐3 by western blotting. The levels of active caspase‐3 dramatically increased in response to 5Z‐7‐oxozeaenol treatment (Figure 7F). Moreover, the release of the proinflammatory factor HMGB1 significantly increased in 5Z‐7‐oxozeaenol‐treated HCC cells, indicating plasma membrane rupture and leakage (Figure 7G). Previous studies have demonstrated that the release of proinflammatory factors, including HMGB1, during pyroptotic cell death can trigger an anti‐tumor immune response by increasing tumor‐associated T cell infiltration.^[28^ ^]^ We analyzed T‐cell infiltrates of syngeneic tumors collected after treatment of mice with DMSO or 5Z‐7‐oxozeaenol. Compared with the DMSO‐treated group, the proportion of CD8+ cells was evidently increased in Hepar1‐6 tumors treated with the TAK1 inhibitor (Figure 7H). Based on these results, we investigated whether TAK1 inhibition enhances the effectiveness of anti‐PD‐1 immunotherapy in mouse models. We administered 5Z‐7‐oxozeaenol or anti‐PD‐1 antibody alone or in combination to mice bearing subcutaneous Hepar1‐6 tumors and analyzed tumor growth. The combined treatment group showed maximal tumor suppression compared to the mice treated with 5Z‐7‐oxozeaenol or anti‐PD‐1 alone (Figure 7I). Collectively, these data indicate that TAK1 inhibition may promote pyroptosis and regulate immune cell infiltration in HCC. Therefore, targeting TAK1 may be a novel therapeutic strategy for the treatment of ESRP2‐deficicent HCC.

## 3 Discussion

Accumulating evidence has revealed that, during cancer initiation and progression, tumor cells reacquire gene expression patterns and phenotypic features of fetal development.^[29^ ^]^ Many oncofetal proteins have been identified in HCC, including the most well‐known oncofetal gene AFP, which is used as a diagnostic marker, and recently identified HLF, PGC7, and CLDN6, which play essential roles in tumor malignant transformation.^[30^ ^]^ Elucidation of oncofetal proteins in HCC is critical, as it not only provides insight into the molecular mechanisms of tumor initiation and progression, but also facilitates the development of anti‐cancer therapies by targeting oncofetal proteins. Current studies have mainly focused on oncofetal drivers that are highly activated in both fetal and tumor tissues. However, few studies have investigated genes that are repressed in fetal hepatocytes, activated in the adult liver, but re‐silenced in HCC, which we define as “oncofetal suppressors.” In the present study, we identified ESRP2 as a novel oncofetal suppressor that inhibits HCC proliferation and migration by promoting the fetal‐to‐adult switch in RNA‐splicing programs.

The downregulation of ESRP2 in HCC might be attributed to several regulatory mechanisms. Transcription factors such as ZEB1/2 have been shown to regulate ESRP2 expression in non‐small‐cell lung cancer (NSCLC) cells.^[31^ ^]^ Also, miRNAs can target 3′UTR of ESRP2 mRNA and regulate its expression in multiple cancer types.^[32^ ^]^ Shen et al. reported that long noncoding RNAs such as Lnc‐LSG1 promotes the ubiquitin‐dependent degradation of ESRP2 by binding to ESRP2.^[33^ ^]^ In the present study, we found that hypermethylation of the ESRP2 promoter was the major cause of ESRP2 downregulation in HCC, which explains the “turn off” mechanism of oncofetal suppressors in tumor tissues.

Previous studies have shown that ESRP2 dictates alternative splicing in epithelial cells, which is essential for mammalian development.^[34^ ^]^ Loss of ESRP2 and its paralog, ESRP1, in mice resulted in developmental defects.^[34^ ^]^ Furthermore, knockdown of both ESRP2 and ESRP1 induced multiple splicing events switching from epithelial to mesenchymal isoforms, indicating the critical role of both factors in the EMT process.^[35^ ^]^ It is noteworthy that ESRP2 has been reported to control the splicing of Hippo components, such as facilitating the expression of adult NF2, HK, YAP1, and TEAD1 isoforms, leading to the dysregulation of Hippo signaling in various diseases.^[^ ^9^, ^10^, ^11^, ^12^ ^]^ Here, we demonstrated a global map of ESRP2‐affected RNA splicing alterations in HCC, in which multiple splice isoforms undergo a fetal‐to‐adult transition. Alternative splicing of TAK1, which has rarely been reported in previous studies, was screened as a determinant event for HCC progression. Interestingly, we found that ESRP2 suppressed the expression of TAK1_ΔE12, the fetal and oncogenic isoform of TAK1, resulting in impaired proliferation and migration of HCC cells.

Increasing evidence suggests that condensates formed by phase separation play indispensable roles in oncogenic processes.^[36^ ^]^ Under specific conditions, proteins and macromolecules can organize into membrane‐free compartments, also known as biomolecular condensates, which are critical for homeostasis because they confer the ability of cells to exercise spatiotemporal control over protein function.^[37^ ^]^ Condensates are usually driven by phase separation and have multiple functions, including concentrating proteins, accelerating biochemical reactions, and regulate gene expression.^[37^ ^]^ While no direct evidence exists in current literatures regarding the impact of TAK1 phase separation on MAPK signaling activation, Mingjian et al. reported the recruitment of TAK1‐TABs into liquid condensates formed by NEMO, resulting in IKK activation.^[38^ ^]^ The authors raised that whether TAK1 or TABs could initiate LLPS require further investigation. In the present study, we found that fetal TAK1 interacts closely with TAB3 protein, and the complex forms liquid condensates within HCC cells, leading to persistent activation of TAK1 and downstream signaling. Our findings provide a link between tumor‐associated alternative splicing and aberrantly altered condensate formation, indicating the pivotal role of biomolecular condensates in cancer initiation and progression. However, whether fetal TAK1‐TAB3 complexes can form liquid condensates in vitro and whether they are driven by liquid‐liquid phase separation (LLPS) requires further investigation. Individual fetal or adult TAK1 and TAB3 proteins should be purified for in vitro LLPS experiments.

Pyroptosis is a form of programmed cell death that has been discovered in recent years and is typically characterized by cell swelling, membrane pore formation, cell lysis, and the release of pro‐inflammatory molecules, thereby triggering the immune response.^[39^ ^]^ Members of the gasdermin superfamily have been shown to be involved in initiating pyroptosis when they are cleaved by caspases. For example, in tumors, GSDME cleaved by caspase 3 induces pyroptosis and activates antitumor immunity.^[40^ ^]^ Here, we demonstrate that TAK1 inhibition in HCC cells leads to GSDME‐mediated pyroptotic cell death and the release of HMGB1, which promotes T cell infiltration, potentially turning “cold tumors” into “hot tumors.” Although our data clearly showed that TAK1 inhibition leads to pyroptosis, other forms of cell death, such as apoptosis, necroptosis, and ferroptosis, cannot be excluded. Further characterization of cell death by TAK1 inhibition should provide deeper insights into the development of novel treatment strategies against HCC.

In summary, unlike adult TAK1, fetal TAK1 serves as an oncogenic factor by enhancing the proliferation and migration of HCC cells. The splicing regulator ESRP2 inhibits HCC progression by repressing exon 12 skipping in the TAK1 pre‐mRNA and suppressing the expression of the fetal TAK1 isoform. ESRP2 and fetal TAK1 are prognostic biomarkers and potential targets for HCC treatment.

## 4 Experimental Section

### In Vitro Hepatocyte Differentiation and Identification of Oncofetal Proteins

hESCs were induced to differentiate along hepatic lineages into fetal hepatocytes as previously described.^[6^ ^]^ Fetal hepatocytes (FH), non‐tumor adult liver tissues (AL), and HCC specimens were collected for RNA sequencing. Genes that reached their peak expression in fetal hepatocytes and were significantly upregulated in tumor tissues were defined as oncofetal drivers. Genes suppressed in both fetal hepatocytes and HCC were defined as oncofetal suppressors. The selection criteria for oncofetal drivers: fold change and expression (calculated as RPKM) cut‐off were AL/FH<0.5, AL/HCC<0.5, FH>2, HCC>2; for oncofetal suppressors: AL/FH>1.5, AL/HCC>1.5, AL>4.

### Human Primary Hepatocyte Isolation

Fetal liver samples were obtained from the University of Hong Kong, Shenzhen Hospital. Adult liver samples were obtained from excised normal tissues adjacent to the hemangioma at Sun Yat‐sen University Cancer Center. The samples used in this study were approved by the Medical Ethical Cfouncil of the University of Hong Kong‐Shenzhen Hospital and the Committee for Ethical Review of Research Involving Human Subjects at the Sun Yat‐sen University Cancer Center. Informed consent was obtained from all patients. Primary human hepatocytes were isolated from these tissues by a two‐step collagenase perfusion technique. Briefly, the tissue was pre‐perfused with calcium‐free buffer comprising 1 × Hanks without Ca^2+^ and Mg^2+^, 5 mg ml^−1^ BSA, and 0.5 mM EDTA for 15–30 min at 37 °C. The tissue was then perfused with 1 × Hanks'solution with Ca^2+^ and Mg^2+^ buffer containing 1 mg ml^−1^ collagenase type IV for 20–40 min at 37 °C. Digestion was stopped by the addition of cold DMEM/F12 (Invitrogen) and 1% FBS (Invitrogen). The suspension was then filtered through a 100 µm Nylon cell strainer, centrifuged for 3 min at 70 g, and resuspended in 35 ml cold DMEM/F12 + 13.5 ml Percoll (density 1.130 g ml^−1^, GE healthcare) + 1.5 ml 10 × HBSS. The cells were pelleted at 100 × g for 10 min and washed three times in cold DMEM/F12.

### Cell Lines and HCC Clinical Specimens

A total of 176 primary HCC samples and their adjacent non‐tumor tissues were obtained from patients who underwent hepatectomy for HCC at Sun Yat‐sen University Cancer Center (Guangzhou, China). All patients gave written informed consent for the use of their clinical specimens for medical research. The samples used in this study were approved by the Committee for Ethical Review of Research Involving Human Subjects at the Sun Yat‐sen University Cancer Center (Guangzhou, China). The human immortalized liver cell lines MIHA and HCC cell lines 97H, Huh7, Hep3B, PLC8024, and SNU182, as well as murine hepatoma cell line Hepar1‐6 were used in this study. STR DNA profiling was performed for cell line authentication. Cells were maintained in high‐glucose Dulbecco's modified Eagle's medium (Gibco BRL, Grand Island, NY, USA) supplemented with 10% fetal bovine serum (Gibco BRL) and 1% penicillin/streptomycin. cells were incubated at 37°C in a humidified incubator containing 5% CO2.

### Antibodies and Reagents

The commercial antibodies used were ESRP2, β‐actin, TAK1, phospho‐TAK1 (Ser439), GSDME (Abcam), p38, phospho‐p38, JNK, phospho‐JNK, p65, phospho‐p65, IKK, phospho‐IKK, cleaved caspase‐3, TAB1, TAB2, GSDMD, anti‐mouse CD8α, FLAG (Cell Signaling Technology), TAB3 (Santa Cruz Biotechnology), InVivoMAb anti‐mouse PD‐1 (CD279), and InVivoMAb rat IgG2a isotype control (Bio X Cell). The TAK1 inhibitor, takinib, was purchased from Cayman Chemical, and 5Z‐7‐oxozeaenol was obtained from Enzo Life Sciences.

### IHC Staining

IHC was performed using rabbit anti‐human ESRP2 (Ab113486) or rabbit anti‐mouse CD8α (CST98941) primary antibodies as previously described.^[41^ ^]^ Immunohistochemistry (IHC) images were acquired using a DM6000B microscope (Leica).

### Western Blot Analysis and Co‐IP

Western blot analysis was performed as described previously.^[41^ ^]^ For co‐IP, 5 mg of total cell lysate was immunoprecipitated with anti‐FLAG or anti‐IgG affinity gel at 4 °C overnight. Extensive washing and immunocomplex denaturation steps were performed according to the manufacturer's instructions (Pierce magnetic co‐IP kit, Thermo Fisher). Denatured immunocomplexes were analyzed by western blotting. ≈5% of the whole lysate (input) was used as a positive control.

### Enzyme Linked Immunosorbent Assay (ELISA)

The release of HMGB1 was measured using an HMGB1 ELISA kit (SEA399Hu, Cloud‐Clone Corp., China), according to the manufacturer's instructions. The absorbance was measured at 450 nm. Three independent experiments were performed.

### DNA Extraction and Bisulfite Sequencing

Genomic DNA was extracted from the cell lines or clinical samples using the phenol‐chloroform method. An EpiTect Bisulfite Kit (Qiagen, Hilden, Germany) was used to treat the DNA for bisulfite conversion. The converted DNA was amplified as a template by PCR using the primers listed in Table S7 (Supporting Information). The PCR product was cloned into a pGEM‐T Easy vector (Promega) and sequenced as individual clones.

### Plasmids and Transfection

ESRP2 shRNA or overexpression lentivirus was obtained from GeneChem Corp. (Shanghai, China), and lentiviral transfection was conducted according to the manufacturer's instructions. The following plasmid constructs were ordered from Genecopoeia: plasmids expressing Flag‐tagged TAK1_FL or TAK1_ΔE12 (pReceiver‐Lv158 vector), EGFP‐fused TAK1_FL and TAK1_ΔE12 (pReceiver‐M29 vector), negative control shRNA (shNTC) or shRNAs targeting TAK1 (shTAK1) (psi‐LVRU6P vector). The lentiviral plasmids were transfected into 293FT cells for virus production. HCC cells or immortalized liver cells were infected with virus supernatants.

### RNA‐Seq and Data Analysis

Total RNA isolated from Vec‐ and ESRP2‐transfected 97H and Huh7 cells was subjected to paired‐end RNA‐Seq using the Illumina Novaseq platform by Novogene. Data analysis for differentially spliced exons/introns was carried out using rMATs software. The Likelihood‐Ratio test was used to compare the splicing difference between Vec‐ and ESRP2‐transfected HCC cells, and FDR < 0.05 was used to further screen the AS targets.

### RNA Extraction, qRT‐PCR, and RT‐PCR

Total RNA was extracted and purified using TRIzol reagent (Invitrogen), following the standard protocol. cDNA was synthesized using the Transcription High Fidelity cDNA Synthesis Kit (Roche). The SYBR Green PCR Kit (Applied Biosystems) was used to conduct qRT‐PCR using the primers listed in Table S7 (Supporting Information). RT‐PCR was performed using DreamTaq DNA polymerase (Thermo Fisher Scientific). The primers flanking TAK1 exon 12 used to distinguish the two mRNA variants were listed in Table S7 (Supporting Information).

### Bisulfite Treatment and Methylation Analysis

Genomic DNA was isolated and purified from HCC clinical samples and cell lines by phenol‐chloroform method. EpiTECT Bisulfite Kit (Qiagen, Hilden Germany) was used to treat DNA. Bisulfite genomic sequencing was carried out using primers listed in Table S7 (Supporting Information).

### Cell Growth, Proliferation and Migration Assays

Cell growth was quantified using XTT. A focus formation assay was conducted to determine the anchorage‐dependent proliferation ability of tumor cells. Briefly, 1 × 10^3^ cells were seeded in a 6‐well plate. Two weeks later, surviving colonies were fixed, stained with crystal violet, and counted. Anchorage‐independent proliferation was assessed using a colony formation assay on soft agar. For the migration assay, cells were suspended in serum‐free DMEM and seeded into culture inserts with an 8 mm microporous filter (Corning). FBS‐containing medium (10%) was placed below the insert. After 48 h, migrated cells were fixed, stained, and counted.

### In Vivo Tumorigenicity and Metastasis Assay

The mice were housed in a pathogen‐free laboratory animal unit at The University of Hong Kong (HKU). All animal procedures were approved by the Committee on the Use of Live Animals in the Teaching and Research of HKU. For the in vivo tumorigenicity assay, the cells were injected subcutaneously into the left and right dorsal flanks of BALB/c nude mice. Tumor formation was monitored weekly by measuring the tumor volume. Tumor volume was calculated as 0.5 × l × w^2^, where l was the length and w was the width of the tumor. For the in vivo metastasis assay, cells were transplanted through intrasplenic injection into 4‐week‐old BALB/c nude mice. All mice were euthanized 6 weeks after injection. Tumor nodules that formed on the liver surface were counted. Hematoxylin and eosin staining was performed to visualize liver metastasis.

### Fluorescence Microscopy

Cells grown on glass‐bottom culture dishes (Nest Scientific) were fixed with 4% paraformaldehyde (PFA, Sigma‐Aldrich), permeabilized with 0.2% Triton X‐100 for 10 min, and washed with PBS. cells were then blocked with 10% BSA for 1 h at 37 °C, and incubated with primary antibodies diluted in 10% BSA at 4 °C overnight. Cells were washed thoroughly with PBS and subsequently incubated with a fluorescence‐labeled secondary antibody (Alexa Fluor 555‐conjugated antibody) for 1 h at room temperature. DAPI was used to stain cell nuclei. The cells were imaged using a Zeiss LSM 800 confocal laser‐scanning microscope.

### Fluorescence Recovery after Photobleaching (FRAP)

The FRAP assay was performed using the FRAP module of a Zeiss LSM 800 Confocal Microscope system. EGFP‐ΔE12 or EGFP‐FL of TAK1 was bleached using a 488‐nm laser beam with 100% laser power. Bleaching was performed on a pre‐selected square region of interest (ROI) and time‐lapse images were collected. Fluorescence intensity was measured and normalized relative to the pre‐bleaching time points using the ZEN software. GraphPad Prism was used to plot and analyze the FRAP results.

### Statistics

Statistical analysis was performed using the SPSS software (version 16.0; International Business Machines Corporation). Two‐tailed Student's *t* tests, Pearson's correlation analysis, Kaplan‐Meier analysis, log‐rank test, Cox's proportional hazards regression model, and Pearson's chi‐square (χ2) tests were used to analyze the corresponding data, as indicated in the figure legends. Data were presented as mean ± standard deviation of three independent experiments. *P*‐values are denoted as ^*^ *p*< 0.05, ^**^ *p*< 0.01, ^***^ *p*< .001 in all figures.

## Conflict of Interest

The authors declare no conflict of interest.

## Author Contributions

Q.Y., X.F., and X.L. contributed equally to this work. Conceptualization, Q.Y. and X.Y.G.; Methodology, Q.Y., X.N.F., X.X.L.; Investigation, Q.Y., X.N.F., X.X.L., S.G., M.L., S.Q.C., and P.L.; Writing–Original Drafts, Q.Y.; Writing–Review & Editing, Q.Y. and X.Y.G.; Funding Acquisition, X.Y.G., P.L., and Q.Y.; Supervision, X.Y.G.

## Supporting information

Supporting Information

## Data Availability

The data that support the findings of this study are available in the supplementary material of this article.
