# Supplementary material for: Loss of ESRP2 Activates TAK1‐MAPK Signaling through the Fetal RNA‐Splicing Program to Promote Hepatocellular Carcinoma Progression
Source: Adv Sci (Weinh). 2023 Nov 20;11(1):2305653. doi: 10.1002/advs.202305653 (PMC10767434; doi:10.1002/advs.202305653)
Supplement: Supplementary file 1 — Supporting Information [file ADVS-11-2305653-s001.pdf]

ADVANCED SCIENCE
Open Access

Supporting Information

for *Adv. Sci.*, DOI 10.1002/advs.202305653

Loss of ESRP2 Activates TAK1-MAPK Signaling through the Fetal RNA-Splicing Program to Promote Hepatocellular Carcinoma Progression

Qian Yan, Xiaona Fang, Xiaoxia Liu, Sai Guo, Siqi Chen, Min Luo, Ping Lan and Xin-Yuan Guan**

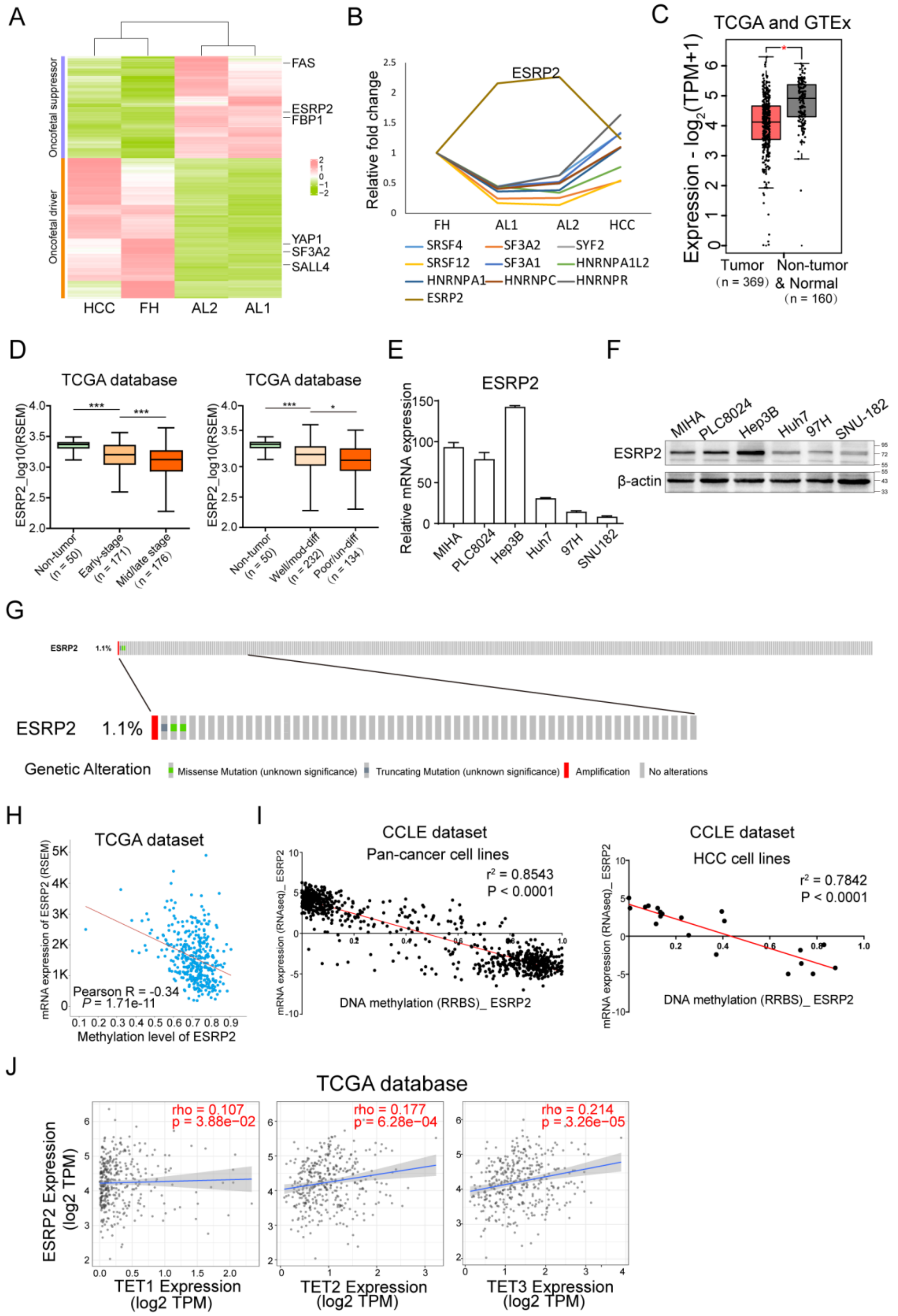

Supplementary Figure 1. ESRP2 is downregulated in HCC and the down-regulation is correlated

with promoter hypermethylation.

(A) Hierarchical clustering analysis was used to illustrate the unique expression patterns of oncofetal drivers or suppressors among fetal hepatocytes (FH), adult liver (AL), and HCC samples. Representative molecules were denoted on the right of the heatmap. **(B)** The expression patterns of various splicing factors across FH, AL and HCC samples. The relative fold change was determined based on gene expression at the FH stage. **(C, D)** The expression of ESRP2 was analyzed in TCGA cohorts divided by tumor and non-tumor **(C)**, different clinicopathological stage or differentiation status **(D)**. **(E, F)** Expression of ESRP2 in HCC cell lines or immortalized liver cell line was detected by qRT-PCR **(E)** or western blot analysis **(F)**. **(G)** The DNA alterations of ESRP2 in HCCs from TCGA dataset were shown. The data were downloaded from cBioportal. **(H)** The association between ESRP2 expression and methylation level in TCGA-HCC cohort. Pearson coefficient R was used to denote the correlation. **(I)** Association between mRNA expression and DNA methylation of ESRP2 in pan-cancer cell lines (left) and HCC cell lines (right) from CCLE dataset (https://sites.broadinstitute.org/ccle/). Pearson coefficient R was used to denote the correlation. **(J)** The correlation between ESRP2 expression and the expression of TET1, TET2, and TET3 in the TCGA-CRC database. Pearson coefficient R was used to quantify the correlation.

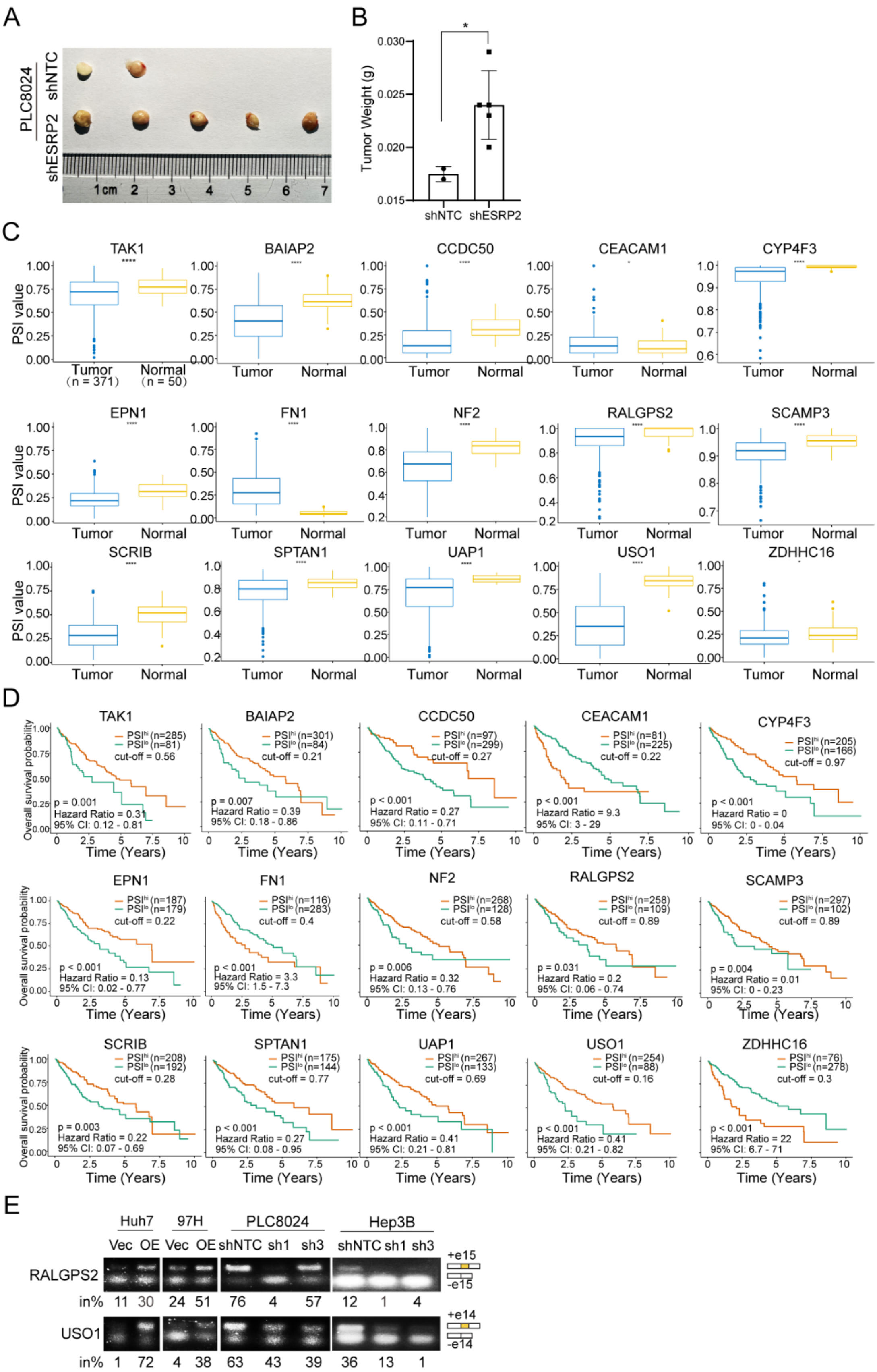
A
PLC8024
shNTC
shESRP2
1 cm 2 3 4 5 6 7
B
Tumor Weight (g)
shNTC
shESRP2
C
PSI value
TAK1
BAIAP2
CCDC50
CEACAM1
CYP4F3
Tumor (n = 371)
Normal (n = 50)
EPN1
FN1
NF2
RALGPS2
SCAMP3
SCRIB
SPTAN1
UAP1
USO1
ZDHHC16
Tumor
Normal
D
Overall survival probability
Time (Years)
TAK1 PSIhi (n=285) PSIlo (n=81) cut-off = 0.56 p = 0.001 Hazard Ratio = 0.31 95% CI: 0.12 - 0.81
BAIAP2 PSIhi (n=301) PSIlo (n=84) cut-off = 0.21 p = 0.007 Hazard Ratio = 0.39 95% CI: 0.18 - 0.86
CCDC50 PSIhi (n=97) PSIlo (n=299) cut-off = 0.27 p < 0.001 Hazard Ratio = 0.27 95% CI: 0.11 - 0.71
CEACAM1 PSIhi (n=81) PSIlo (n=225) cut-off = 0.22 p < 0.001 Hazard Ratio = 9.3 95% CI: 3 - 29
CYP4F3 PSIhi (n=205) PSIlo (n=166) cut-off = 0.97 p < 0.001 Hazard Ratio = 0 95% CI: 0 - 0.04
EPN1 PSIhi (n=187) PSIlo (n=179) cut-off = 0.22 p < 0.001 Hazard Ratio = 0.13 95% CI: 0.02 - 0.77
FN1 PSIhi (n=116) PSIlo (n=283) cut-off = 0.4 p < 0.001 Hazard Ratio = 3.3 95% CI: 1.5 - 7.3
NF2 PSIhi (n=268) PSIlo (n=128) cut-off = 0.58 p = 0.006 Hazard Ratio = 0.32 95% CI: 0.13 - 0.76
RALGPS2 PSIhi (n=258) PSIlo (n=109) cut-off = 0.89 p = 0.031 Hazard Ratio = 0.2 95% CI: 0.06 - 0.74
SCAMP3 PSIhi (n=297) PSIlo (n=102) cut-off = 0.89 p = 0.004 Hazard Ratio = 0.01 95% CI: 0 - 0.23
SCRIB PSIhi (n=208) PSIlo (n=192) cut-off = 0.28 p = 0.003 Hazard Ratio = 0.22 95% CI: 0.07 - 0.69
SPTAN1 PSIhi (n=175) PSIlo (n=144) cut-off = 0.77 p < 0.001 Hazard Ratio = 0.27 95% CI: 0.08 - 0.95
UAP1 PSIhi (n=267) PSIlo (n=133) cut-off = 0.69 p < 0.001 Hazard Ratio = 0.41 95% CI: 0.21 - 0.81
USO1 PSIhi (n=254) PSIlo (n=88) cut-off = 0.16 p < 0.001 Hazard Ratio = 0.41 95% CI: 0.21 - 0.82
ZDHHC16 PSIhi (n=76) PSIlo (n=278) cut-off = 0.3 p < 0.001 Hazard Ratio = 22 95% CI: 6.7 - 71
E
Huh7 97H PLC8024 Hep3B
Vec OE Vec OE shNTC sh1 sh3 shNTC sh1 sh3
RALGPS2
+e15
-e15
in% 11 30 24 51 76 4 57 12 1 4
USO1
+e14
-e14
in% 1 72 4 38 63 43 39 36 13 1

Supplementary Figure 2. The expression and prognostic value of ESRP2-regulated AS targets in HCC.

(A, B) Images of xenograft tumors induced by subcutaneous injection of indicated cells into nude mice **(A)**. The average tumor weight was expressed as the mean ± SD **(B)**. **(C)** The PSI index of each AS target was analyzed in TCGA cohort divided by tumor and non-tumor. Student's *t*-test was used to compare the means between two groups. $^{*}P < 0.05$, $^{****}P < .0001$. **(D)** Kaplan-Meier analysis of the OS of HCC patients from TCGA cohort stratified by the PSI level of AS targets. The *P* values of the log-rank tests, Hazard ratio (HR), and 95% confidence interval (CI) were presented. The optimal cutoff point was determined and survival curves were generated using survminer R package. **(E)** RT-PCR validation of ESRP2-regulated AS events. The structure of each PCR product was indicated schematically on the right. Alternative exons affected by ESRP2 were painted in orange. The percentage of exon inclusion products (in%) out of the total products was indicated below each gel.

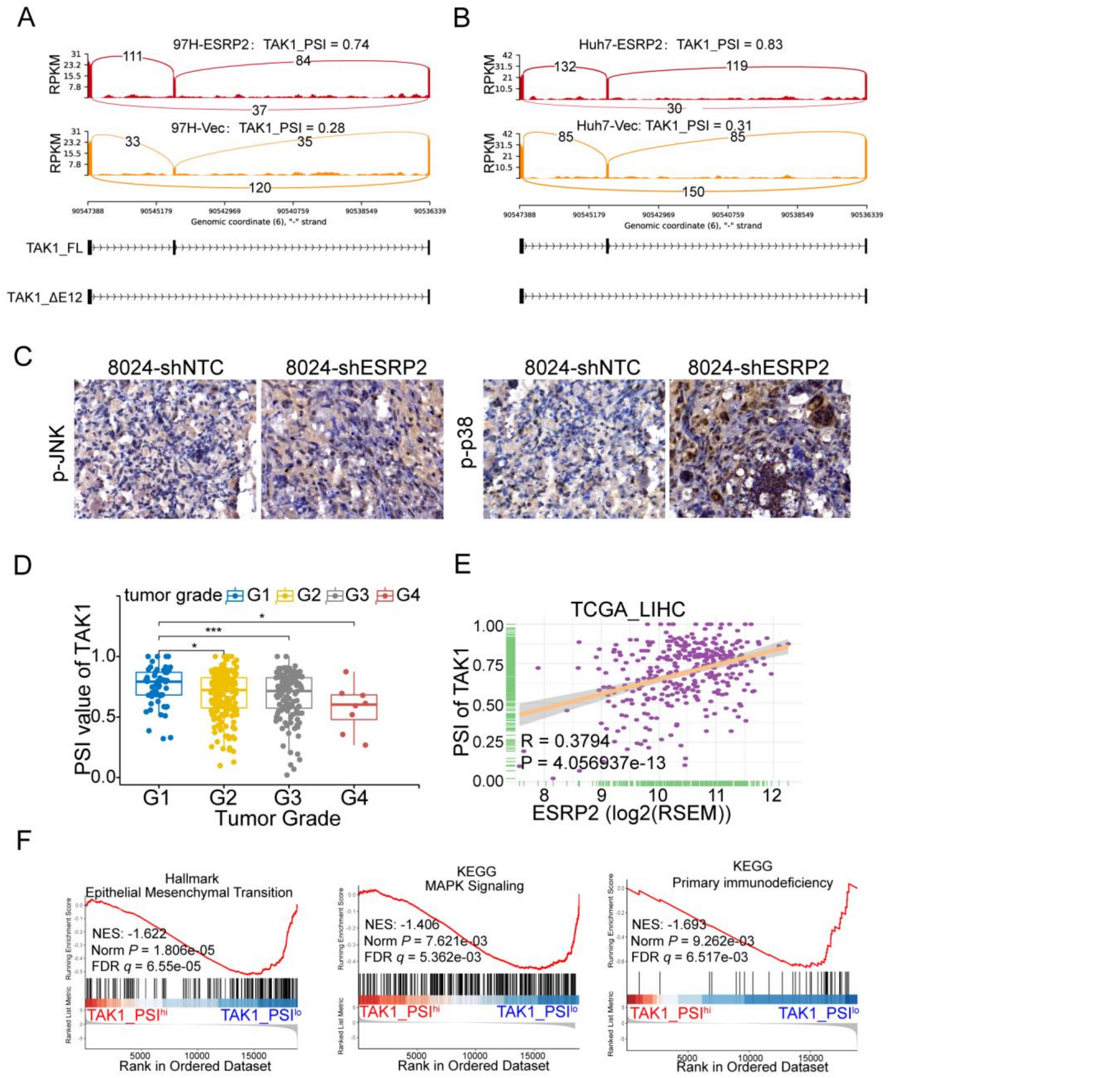

Supplementary Figure 3. ESRP2-regulated TAK1 splicing was correlated with MAPK signaling activation.

(A, B) IGV visualization of TAK1 variants expression in Vec- or ESRP2- transfected 97H **(A)** or Huh7 **(B)** cells. The PSI index was calculated based on the RNA-seq data. **(C)** Representative images of IHC staining for p-JNK and p-p38 in xenograft tumors induced by shNTC- or shESRP2- transfected HCC cells. **(D)** The PSI index of TAK1 exon 12 was analyzed in TCGA-HCC cohorts divided by different

tumor grade. **(E)** The association between ESRP2 expression and the PSI index of TAK1 exon 12 in the TCGA-HCC cohort was analyzed. Pearson coefficient R was used to denote the correlation. **(F)** Representative gene sets enriched by GSEA analysis of DEGs from HCC specimens with low or high TAK1 PSI index in the TCGA cohort.

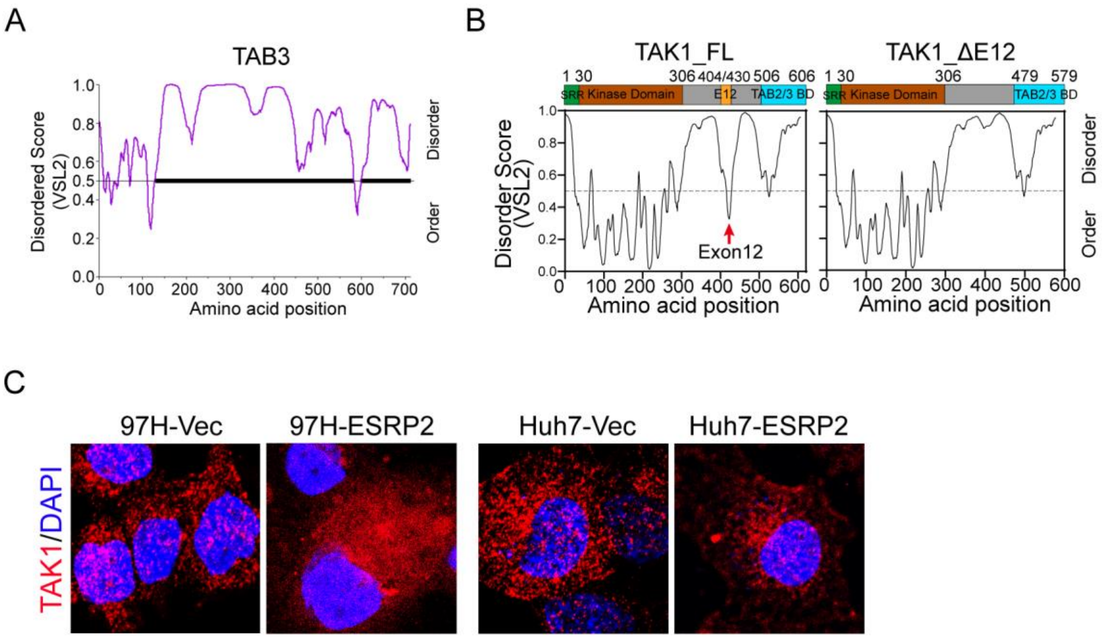

Supplementary Figure 4. Graph of intrinsic disorder of TAB3 and TAK1 isoforms.

(A) Graph of intrinsic disorder of TAB3 as calculated by the VSL2 algorithm (http://www.pondr.com/). **(B)** Graph of intrinsic disorder of TAK1_FL (left) and TAK1_ΔE12 (right) as visualized by the VSL2 algorithm. The serine rich region (SRR), kinase domain, TAB2/3 binding domain and exon 12 position were indicated above the disorder score graph. **(C)** Immunofluorescence staining of TAK1 (red) in Vec- or ESRP2- transfected HCC cells. Cell nuclei were counterstained with DAPI (blue).

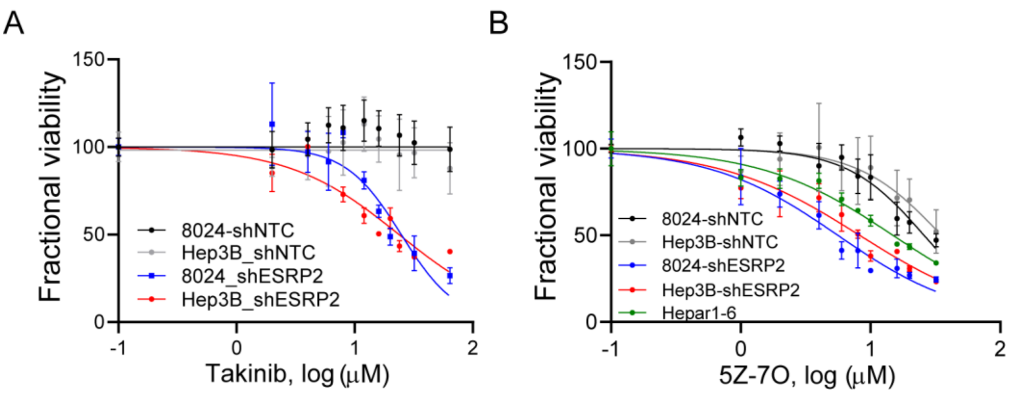

Supplementary Figure 5. HCC cells with low expression of ESRP2 are sensitive to TAK1 inhibitors.

(A, B) PLC8024 and Hep3B cells transfected with shNTC or shESRP2, as well as ESRP2-low-expressed Hepar1-6 cells, were treated with varying concentrations of Takinib **(A)** or 5Z-7-oxozeaenol **(B)** for 48 h. Cell viability was determined by XTT assay. Data were represented as means ± SD.

Supplementary Table 1. Relative expression value (RPKM) of oncofetal drivers or suppressors identified from RNA-seq data

Gene_name	Fetal hepatocytes	Adult liver 1	Adult liver 2	HCC	Type
A2M	270.6283096	576.3739758	850.6861498	297.9099072	Oncofetal suppressor
FTL	1026.025848	2996.0248	3330.526934	1298.174822	Oncofetal suppressor
ENG	1.628205211	13.28095459	16.09747711	4.196338601	Oncofetal suppressor
CXCL12	0.818101177	44.32602377	37.80556619	2.472448972	Oncofetal suppressor
PHYHD1	1.572662101	6.576480437	5.798253746	1.90358389	Oncofetal suppressor
SORL1	2.436358443	11.92096292	14.94488348	6.037531137	Oncofetal suppressor
CLIC2	0.126412757	5.060803714	8.077838848	1.331457362	Oncofetal suppressor
CDA	1.218789026	24.22624101	15.88834167	11.22224491	Oncofetal suppressor
ITGB2	0.103606929	3.071871861	5.271404027	1.382582067	Oncofetal suppressor
ACSS3	8.506874961	8.356253811	22.21017239	1.308638057	Oncofetal suppressor
FUCA1	11.02899172	34.26172395	40.22890748	16.07880355	Oncofetal suppressor
ECHDC3	0.492922971	19.959834	36.69029951	5.017686662	Oncofetal suppressor
LGMN	11.33268036	20.35358362	26.10362964	12.17730106	Oncofetal suppressor
DHTKD1	5.624192013	45.23365382	47.62041977	27.73863413	Oncofetal suppressor
GZMA	0	5.689077436	9.107655656	1.051164464	Oncofetal suppressor
FCGR3A	0	32.8200919	51.92706499	12.96489977	Oncofetal suppressor
TSPAN33	1.078762713	5.345801586	5.30287274	1.810779993	Oncofetal suppressor
ALDH8A1	0.621078449	55.59920836	70.86056444	32.22811813	Oncofetal suppressor
RANBP10	2.362621813	4.33882036	5.052563128	1.898053565	Oncofetal suppressor
LY96	0.682446263	8.61643143	16.52899022	1.732479346	Oncofetal suppressor
PROZ	11.48891961	10.84332259	37.9775951	1.738626562	Oncofetal suppressor
ZFP36	28.07527871	40.8874681	92.82328616	32.23507915	Oncofetal suppressor
FNDC4	5.225086803	9.267094995	11.78886958	0.891830539	Oncofetal suppressor
DGAT2	6.650668915	37.00040158	35.81900006	10.47563411	Oncofetal suppressor
ORM2	1.374940459	585.59722	504.4956483	341.5249318	Oncofetal suppressor
KYNU	2.984433577	15.17680722	15.07936874	4.293700719	Oncofetal suppressor
42065	4.78754059	64.00773704	53.79343177	28.44794276	Oncofetal suppressor
GNG11	5.743851922	25.76040488	27.26036986	16.98998307	Oncofetal suppressor
AIF1	1.548187942	20.99682069	29.74556021	6.356473422	Oncofetal suppressor
ALAS1	17.15675108	45.76191034	146.5797501	18.62506868	Oncofetal suppressor
PECR	3.857780221	38.01160237	36.70911475	13.6484791	Oncofetal suppressor
GIMAP4	0.762568549	26.53083455	32.66225206	11.10059008	Oncofetal suppressor
NDUFA4	12.46074077	22.54844544	22.30577898	13.26379354	Oncofetal suppressor
SUCLG2	38.30983141	76.40747864	83.49674754	28.42828854	Oncofetal suppressor
AKR7A2	5.752755049	8.254992879	9.360121652	4.572949054	Oncofetal suppressor
UGT2B4	0.149787221	258.0812335	405.9191942	32.20027523	Oncofetal suppressor
MGST2	4.100789999	17.31035688	15.94123929	6.588678472	Oncofetal suppressor
MSR1	0.383560076	4.024938169	6.831587125	3.485677816	Oncofetal suppressor

GCSH	4.950373233	27.56815976	25.33931736	14.50790674	Oncofetal suppressor
C19orf60	1.94790919	8.995488651	6.102216207	4.250954876	Oncofetal suppressor
C1QTNF1	1.812072447	4.259085431	5.978327939	0.852767922	Oncofetal suppressor
ADK	15.20386651	22.5407429	28.46706669	6.528492271	Oncofetal suppressor
GLT1D1	0.21860937	3.597835375	7.792189884	1.333212664	Oncofetal suppressor
ARSE	9.282798471	28.06322732	22.24975028	7.734599592	Oncofetal suppressor
APOH	2.280627477	5336.515553	5245.633526	1556.021935	Oncofetal suppressor
HAL	0.751318609	29.54638257	31.18376793	11.95698848	Oncofetal suppressor
QTRT1	1.350531624	4.052335664	4.998020402	2.360825126	Oncofetal suppressor
NADKD1	7.258825359	42.05501719	52.21934347	21.11062246	Oncofetal suppressor
PSAT1	11.44790091	47.53488653	66.38408338	7.291235253	Oncofetal suppressor
BRI3	6.714408798	10.46532586	10.40444645	3.494962718	Oncofetal suppressor
SHH	1.800707207	4.09127173	4.557434844	1.154713076	Oncofetal suppressor
NPY1R	0.657374254	7.566540214	6.448397405	0.471048136	Oncofetal suppressor
PQLC1	1.63858142	6.978061614	6.315429254	1.987489705	Oncofetal suppressor
PCBD1	11.84045676	89.25420399	77.56021084	22.63818232	Oncofetal suppressor
TNFRSF1B	3.233881977	7.321206759	10.8614352	5.210485319	Oncofetal suppressor
PHLDA1	2.414588872	1.898609418	11.37005266	3.399614349	Oncofetal suppressor
C1QB	0	116.1520612	151.9691467	18.4057425	Oncofetal suppressor
PTH1R	2.820753126	16.49689714	6.405802595	0.251675755	Oncofetal suppressor
NAAA	1.694664669	9.377842048	7.116191981	3.483358177	Oncofetal suppressor
CD163	0.800507627	21.4378391	35.44888666	3.133162939	Oncofetal suppressor
ACSM3	2.040735335	21.31061552	16.41634928	2.511948547	Oncofetal suppressor
ZFP1	2.272601097	9.192418189	10.23973869	2.094103916	Oncofetal suppressor
ICAM1	2.705481052	6.65440736	18.12343769	2.150626423	Oncofetal suppressor
CHRD	0.765497843	3.804090832	4.765313067	1.216755758	Oncofetal suppressor
ATP6V0E2	0.505126193	7.359746028	7.399568185	1.314979953	Oncofetal suppressor
RNMTL1	2.36711361	4.058417892	4.718489493	2.871340633	Oncofetal suppressor
C3AR1	0.154496926	3.830257837	7.259018586	2.315712163	Oncofetal suppressor
PCOLCE	2.009455165	6.861801944	4.260941562	2.248881423	Oncofetal suppressor
42064	1.400176741	11.20299331	13.21720733	2.403300797	Oncofetal suppressor
CYP4F12	0	7.011256124	13.13578073	6.313946464	Oncofetal suppressor
CTSC	1.470061348	3.793930219	5.634518114	1.32643652	Oncofetal suppressor
IGFBP4	77.05342004	201.3768839	265.5316751	128.571534	Oncofetal suppressor
HAMP	0.162832108	1458.33542	1172.818445	5.646467334	Oncofetal suppressor
GYS2	0.224845562	76.92128294	66.77273006	7.879753974	Oncofetal suppressor
GCKR	0.837522731	27.95412283	27.58446087	5.023491272	Oncofetal suppressor
MT1A	0.278952782	17.78333996	4.726157805	0.113003972	Oncofetal suppressor
MT1M	0.102608428	39.65233719	13.35452247	0.023752427	Oncofetal suppressor
ZGPAT	0.886438311	11.67551543	7.092518479	1.864247189	Oncofetal suppressor
SUOX	2.36293839	8.309681995	9.558596176	3.641264168	Oncofetal suppressor
STEAP3	0.84671314	18.84362396	24.56732421	1.965449096	Oncofetal suppressor
SORD	9.889236204	144.4662912	93.26731544	14.92015716	Oncofetal suppressor
IL7R	0.205071724	4.26775447	7.676376308	1.01904968	Oncofetal suppressor

AADAC	0.390014212	170.224182	215.1489877	116.2171149	Oncofetal suppressor
ABHD14B	6.871319193	49.52058655	45.13221211	25.60961005	Oncofetal suppressor
POLB	2.505377796	3.044052629	6.235677529	1.950845969	Oncofetal suppressor
VSIG4	0	9.882290145	10.36018673	1.08199241	Oncofetal suppressor
KHK	0.135374144	20.05290278	21.82286887	11.07040087	Oncofetal suppressor
ARHGAP30	0.286306692	5.005491942	7.528785317	3.026605196	Oncofetal suppressor
CYP39A1	0.160277654	26.19715078	28.90429963	4.360053356	Oncofetal suppressor
ACSM1	1.433240158	2.201849311	10.66535718	3.764201272	Oncofetal suppressor
NR2F1	0.663948791	6.950338557	5.488414385	1.448688863	Oncofetal suppressor
CCND1	12.72466507	21.0346081	41.72867174	7.092297732	Oncofetal suppressor
SLC17A3	0.088366094	6.972602642	4.874605183	1.058351056	Oncofetal suppressor
SLC17A1	0.520170514	18.03244226	19.28497789	4.871223076	Oncofetal suppressor
PROSC	9.260395678	20.71715768	19.63715461	8.00774972	Oncofetal suppressor
LEPR	3.420403755	21.02437599	22.76529064	11.94025001	Oncofetal suppressor
GSTZ1	1.053896577	6.58521176	4.943668121	0.453988825	Oncofetal suppressor
MGST1	34.74683099	143.6404751	170.467734	96.54840645	Oncofetal suppressor
PAFAH2	3.013672021	7.259283108	7.486334587	4.072386164	Oncofetal suppressor
N4BP2L1	2.631259127	22.84051959	18.00790131	11.41098688	Oncofetal suppressor
SELL	0.243226353	5.002347912	6.740655191	0.936046112	Oncofetal suppressor
HINT2	1.014712584	8.924301753	9.23231832	2.148726882	Oncofetal suppressor
MPDZ	11.55069409	24.653589	20.96661169	7.383500893	Oncofetal suppressor
CREB3L3	22.61313579	42.16983159	43.66238624	17.60738222	Oncofetal suppressor
DNASE2	3.544782073	10.16244072	10.23276069	6.311693489	Oncofetal suppressor
KIAA0664	2.87902988	14.25844823	13.98259401	5.972217434	Oncofetal suppressor
EPHX1	18.76013163	369.6307729	399.9893007	161.9938113	Oncofetal suppressor
KLRB1	0.14304036	4.451006068	8.381345694	0.988940837	Oncofetal suppressor
SDSL	0.448640666	10.12836054	10.23056881	2.25161434	Oncofetal suppressor
NINJ1	5.699517318	9.440241763	11.88413702	3.286930486	Oncofetal suppressor
ASGR2	8.609339631	95.81447104	103.3806336	24.83296266	Oncofetal suppressor
CCL21	0.014943899	33.65017962	42.70247148	1.162326569	Oncofetal suppressor
DECR2	1.996990722	3.113112117	4.947785995	2.162933672	Oncofetal suppressor
SNX10	4.512577003	16.32973474	27.70928759	7.083277907	Oncofetal suppressor
ACAA2	55.97556292	368.575663	377.3740506	45.03895192	Oncofetal suppressor
BST2	26.97465927	50.12558191	92.67872538	31.43267586	Oncofetal suppressor
SERPING1	37.24716446	660.5368958	586.1422315	126.9705478	Oncofetal suppressor
FAH	1.968684676	16.48639928	13.53040486	8.923937903	Oncofetal suppressor
A1BG	1.860896222	111.6660615	157.8514966	26.07435476	Oncofetal suppressor
RCAN1	8.486079564	5.456811404	23.10955591	4.798458197	Oncofetal suppressor
C8orf4	23.92510901	22.05160707	56.40959248	9.08174638	Oncofetal suppressor
UROS	3.126439154	5.677949027	6.360286786	2.729551667	Oncofetal suppressor
RNF125	0.411613947	6.7772629	3.720727394	1.248009754	Oncofetal suppressor
GIMAP2	0.026338889	4.467817006	5.1893426	2.400728159	Oncofetal suppressor
APOA2	240.6419386	5375.293063	6760.375938	335.976952	Oncofetal suppressor
ICAM3	1.51439355	6.856024498	7.216415969	1.862468163	Oncofetal suppressor

SLC39A14	21.68746422	43.36167353	43.3561812	23.49916376	Oncofetal suppressor
NAGLU	2.235134254	5.592224095	6.503190336	2.156993801	Oncofetal suppressor
MPDU1	3.361253067	7.953128667	8.473022457	2.675394954	Oncofetal suppressor
CETP	0.0993393	7.950093415	11.88202973	0.300476748	Oncofetal suppressor
DDO	0	4.000420049	5.529226223	2.9530175	Oncofetal suppressor
BLVRB	10.76842667	72.38277481	67.62083299	37.91137703	Oncofetal suppressor
CBS	9.990861483	56.87978554	49.31046563	10.07569411	Oncofetal suppressor
CRYAA	0.06066189	2.973912685	6.166584814	0	Oncofetal suppressor
PCK1	4.361176105	482.8573007	212.8177232	37.11944141	Oncofetal suppressor
ACSM2B	0.731468583	116.666836	109.3784081	54.71190883	Oncofetal suppressor
ECSIT	6.399301829	9.955668223	9.920242925	3.878648122	Oncofetal suppressor
GBP4	1.235004598	10.64681489	14.31257177	5.66034258	Oncofetal suppressor
BOK	4.778145366	18.555423	16.84075001	4.55094713	Oncofetal suppressor
SLC17A9	1.20204988	6.967927094	8.497295892	4.854529559	Oncofetal suppressor
MYD88	6.438501547	10.02977464	14.68000925	6.007369336	Oncofetal suppressor
FXYD1	1.162577955	205.5473724	143.0693878	52.54935073	Oncofetal suppressor
LAPTM5	4.757363334	22.93715233	36.0606415	11.747788	Oncofetal suppressor
UROC1	0	35.8815736	38.9307906	0.409991581	Oncofetal suppressor
SORBS2	2.655987307	10.30669504	13.85899667	7.112320042	Oncofetal suppressor
HLF	0.457971776	14.57259497	29.43983881	6.356974007	Oncofetal suppressor
SOD1	13.85603649	124.2511268	121.2901169	63.10155226	Oncofetal suppressor
MRPL34	10.08509406	31.37610818	30.67573185	10.26281527	Oncofetal suppressor
SLC43A1	0.554827138	15.09674358	16.72838003	8.870140297	Oncofetal suppressor
CYP2A6	0.25846249	481.4004331	375.4695821	33.89849948	Oncofetal suppressor
MST1	13.8651988	24.86015047	23.73471413	8.608395414	Oncofetal suppressor
ACBD4	2.107350416	11.5451587	13.20882356	7.561246872	Oncofetal suppressor
TBX15	0.141491946	4.582442636	14.37702781	3.174562449	Oncofetal suppressor
ARHGAP25	0.145375465	3.890992268	4.973329598	1.226572065	Oncofetal suppressor
ATP5D	8.520815994	22.7227908	20.55485676	5.262010078	Oncofetal suppressor
CHCHD10	4.304897791	50.25169957	39.12794359	11.12158503	Oncofetal suppressor
C16orf70	3.61118757	6.596789857	6.22984878	2.404398677	Oncofetal suppressor
MT2A	14.21209666	392.7076784	297.0359968	8.508964206	Oncofetal suppressor
MT1G	1.785855713	654.0325571	263.6131939	1.849649013	Oncofetal suppressor
AMFR	8.522787483	12.01572631	15.68551025	6.46995822	Oncofetal suppressor
PHYH	2.854748816	91.46382086	129.1373476	54.57039013	Oncofetal suppressor
CBR4	6.663586944	20.36154877	19.93253026	6.664271031	Oncofetal suppressor
CNDP1	0.038478926	9.149428839	3.693817689	0.280581131	Oncofetal suppressor
AQP9	0	166.1811384	182.733342	49.77784792	Oncofetal suppressor
ADRA1A	0.042501185	8.316774764	10.46873914	0.443822713	Oncofetal suppressor
LAP3	6.621351479	22.16345955	36.79957713	14.5539562	Oncofetal suppressor
PRKACA	12.34070394	22.27322193	26.06764142	15.50677681	Oncofetal suppressor
CSF1R	0.989169491	7.843138133	12.82548734	1.66366593	Oncofetal suppressor
GIMAP5	0	3.117194202	5.054747064	0.79764203	Oncofetal suppressor
AKR1CL1	0	6.496364626	4.399597361	0.808140677	Oncofetal suppressor

ETFDH	5.184591702	42.81858792	43.81073818	8.909079223	Oncofetal suppressor
GBP7	0.011253596	27.27877237	21.09491523	7.877675423	Oncofetal suppressor
LACTB2	5.591912611	14.07583178	18.98466759	8.864169668	Oncofetal suppressor
FCHSD2	3.12160604	5.390454026	5.918447117	3.731835302	Oncofetal suppressor
GSTK1	12.02498939	44.72798227	46.62969662	15.64859118	Oncofetal suppressor
OGDHL	1.032476179	18.04790875	17.91913401	2.040467946	Oncofetal suppressor
NIPAL1	1.692256024	4.344781726	4.526010309	2.207176727	Oncofetal suppressor
GOT1	5.960245467	46.33805656	58.35686904	24.61395503	Oncofetal suppressor
CPN1	3.629490573	16.99022472	18.53777361	7.458262148	Oncofetal suppressor
DYSF	1.896852309	3.705243827	4.399108768	2.516029527	Oncofetal suppressor
APOC4	0.083136174	66.66511188	53.88074315	16.44736628	Oncofetal suppressor
AC020907.3	0.933648013	6.613377315	6.025213985	2.90633347	Oncofetal suppressor
SLC22A10	0	9.303617271	35.78521824	4.827986348	Oncofetal suppressor
MGLL	3.298250484	12.3084644	16.656209	9.258302147	Oncofetal suppressor
UBE2L6	9.603492328	16.16744727	23.99287593	12.17017287	Oncofetal suppressor
SMOC1	0.398221218	16.48147205	13.61517257	8.234986633	Oncofetal suppressor
AVPI1	3.260129017	9.200339373	14.65951963	4.545598344	Oncofetal suppressor
EXOC3L4	0.103164434	4.757285499	4.368965786	1.716686201	Oncofetal suppressor
TRIM22	2.853381135	6.923033412	18.6662271	6.411379836	Oncofetal suppressor
DIO1	5.996445776	39.74776224	51.20376511	10.1382687	Oncofetal suppressor
GBP5	0.03704629	2.717038667	6.150683801	0.673972226	Oncofetal suppressor
ARID3C	0.088074745	7.223714877	4.703954356	1.316163908	Oncofetal suppressor
CECR1	0.556424025	5.245887237	7.940932887	2.071110733	Oncofetal suppressor
ACSL5	12.56741073	51.3779467	47.66409235	25.30765563	Oncofetal suppressor
MT1H	0.076393708	62.37179291	20.44194157	0.092841438	Oncofetal suppressor
SLC39A5	9.593890533	20.59123574	24.37889188	1.803548063	Oncofetal suppressor
HCLS1	0.281965231	8.272073105	11.49785135	4.173580821	Oncofetal suppressor
RDH16	0.223484715	59.95263261	53.75112417	4.287422952	Oncofetal suppressor
CD36	0.189399108	7.838193661	13.38516404	4.914926057	Oncofetal suppressor
CISD3	0.601482116	6.45650839	6.216526533	3.411251707	Oncofetal suppressor
HADH	7.150335886	25.61218557	28.83526911	9.315572566	Oncofetal suppressor
ENPEP	1.123837904	9.553995563	11.39068017	2.962465316	Oncofetal suppressor
TMEM220	1.798839899	15.88366132	15.06482593	5.963633217	Oncofetal suppressor
XAF1	0.200868594	17.78109409	26.74250239	10.45588401	Oncofetal suppressor
ECI1	1.746301347	6.908315021	7.487474916	4.268782695	Oncofetal suppressor
ALDH6A1	16.72896122	106.4794994	82.17168885	27.99862873	Oncofetal suppressor
GADD45G	3.143916022	8.146154533	5.210368399	3.08034125	Oncofetal suppressor
FBXW5	6.292649803	8.402389246	11.1186157	4.678614632	Oncofetal suppressor
SULT2A1	4.12785539	377.8341495	551.7407139	75.5810602	Oncofetal suppressor
FECH	2.521924838	5.548090642	6.614231964	1.878534124	Oncofetal suppressor
LPXN	1.432869744	4.824375459	5.365208901	1.959040888	Oncofetal suppressor
SLC1A2	0.028300055	10.06097434	11.07396429	0.106389376	Oncofetal suppressor
ECHS1	15.47334005	188.0080423	197.7289989	64.68900843	Oncofetal suppressor
PIGV	1.955680055	5.296976669	3.902241728	1.819357266	Oncofetal suppressor

CFP	0.057332843	4.436835323	5.118754974	0.239590152	Oncofetal suppressor
CTSD	9.664332631	50.60953606	81.32735271	27.21681259	Oncofetal suppressor
ASL	2.914480876	15.9746266	20.55537217	9.487245284	Oncofetal suppressor
LBP	14.89253655	54.62295172	92.61517149	9.470205379	Oncofetal suppressor
SPRYD4	0.722940457	14.14238609	13.60068168	3.02448327	Oncofetal suppressor
42065	6.287870094	8.664071869	13.40182972	3.450634946	Oncofetal suppressor
KMO	0.192747647	9.627251153	14.11873701	2.069648931	Oncofetal suppressor
NR1H3	4.768994996	10.64622796	10.03345223	6.002991485	Oncofetal suppressor
MCOLN1	1.758586762	3.788010348	4.418189927	1.324065407	Oncofetal suppressor
COQ10A	1.213483311	3.771567693	4.47406781	2.43549171	Oncofetal suppressor
MOCOS	0.562254608	4.819225386	10.7301309	2.248969731	Oncofetal suppressor
ASPA	0.099067789	6.422889727	3.938645171	1.643518649	Oncofetal suppressor
EHHADH	2.74694301	121.9702134	127.5812564	41.31242484	Oncofetal suppressor
DCAF11	4.342504089	25.87361732	20.62223797	9.851953212	Oncofetal suppressor
FPR3	0.071317487	4.916699515	12.47966292	4.258054313	Oncofetal suppressor
GNPNAT1	5.152659376	19.69239158	21.09920829	12.6356988	Oncofetal suppressor
C2orf42	1.453090403	5.831347201	4.129962078	3.192875862	Oncofetal suppressor
ADCK3	2.644040907	7.85937124	6.765542806	2.9442774	Oncofetal suppressor
TST	5.234843937	28.78471962	33.71233458	13.22624452	Oncofetal suppressor
FABP1	7.61945753	444.3891559	763.152618	86.24430077	Oncofetal suppressor
GPSM3	1.616165756	4.520298245	5.338746571	2.522292539	Oncofetal suppressor
PIGR	0.015777151	6.719443473	22.90109784	5.542565929	Oncofetal suppressor
AGT	62.49534671	494.9446398	438.3900502	188.5595818	Oncofetal suppressor
LILRB2	0.00362419	2.983297147	5.114793073	0.552028852	Oncofetal suppressor
HIBADH	13.67144824	76.84262705	71.85363978	20.29430695	Oncofetal suppressor
SARDH	0.819475533	21.90491992	13.90543512	5.079142917	Oncofetal suppressor
LYVE1	0.439975598	19.85311463	11.52724797	0.687475657	Oncofetal suppressor
GIMAP6	0.112392022	4.627042217	5.681757226	2.035845394	Oncofetal suppressor
TMEM56	7.127937014	28.84497749	28.85300008	11.11484595	Oncofetal suppressor
CD4	0.753227292	33.59835498	24.16538426	3.900819195	Oncofetal suppressor
ECM2	4.250374687	16.60079626	17.91941297	6.599276094	Oncofetal suppressor
LYPLAL1	3.83146255	5.928831868	10.75959758	3.907666682	Oncofetal suppressor
SAMD9L	0.046446518	3.353471048	7.504135722	3.474598932	Oncofetal suppressor
COX5B	16.39539655	38.25311456	39.04408136	21.28787801	Oncofetal suppressor
SLC47A1	1.105811948	25.57432199	24.44949172	6.18544105	Oncofetal suppressor
C11orf71	1.286217081	7.833084269	4.893175575	2.111082615	Oncofetal suppressor
ETNK2	3.217349448	11.80186145	11.44464476	1.328623291	Oncofetal suppressor
ABCC9	7.355648544	14.26836876	13.85967959	7.885344141	Oncofetal suppressor
CYP4F2	0	44.85917807	67.94340717	15.77089513	Oncofetal suppressor
APOF	0.144925323	189.4513754	95.0770389	0.678419181	Oncofetal suppressor
CPN2	0.684023913	95.03385716	112.5741324	25.04232208	Oncofetal suppressor
RP3-402G11.5	3.412597136	11.48508197	13.03985808	6.609225958	Oncofetal suppressor
S100A9	1.172616939	22.73243876	27.75453461	4.265557208	Oncofetal suppressor
CES1	0.023507257	291.7267004	314.327542	44.85622832	Oncofetal suppressor

BRP44L	11.95198347	65.62190537	105.5436264	49.72337787	Oncofetal suppressor
PROC	2.374229182	85.59186608	82.70693164	14.68037091	Oncofetal suppressor
LRAT	0.191729316	4.944946324	5.490820724	0.417197155	Oncofetal suppressor
MMAB	2.638909085	5.961038448	8.628713267	1.609283382	Oncofetal suppressor
UGT1A4	0	19.00729453	14.42108519	4.879513301	Oncofetal suppressor
SQRDL	1.895939442	4.627693524	4.261267062	2.840322631	Oncofetal suppressor
CYP2C18	0.116928245	30.2924184	27.16665857	2.333936538	Oncofetal suppressor
ABAT	6.644753184	59.31814319	51.63791234	24.79504762	Oncofetal suppressor
ADH1A	0	243.4106574	471.6622026	121.0101382	Oncofetal suppressor
TENC1	2.467144854	7.607536743	6.794648422	2.930591082	Oncofetal suppressor
CLEC1B	0.090298612	7.288287528	8.117324391	0.018290016	Oncofetal suppressor
CD1D	1.556712531	3.975671348	6.003278571	0.744833104	Oncofetal suppressor
ACAT1	4.240866596	94.32364237	132.3830614	44.06438512	Oncofetal suppressor
TTC38	5.974017859	23.92335578	30.25200721	15.10829627	Oncofetal suppressor
TDO2	1.280088034	121.2434179	141.2187148	5.691458786	Oncofetal suppressor
PIPOX	0.640023322	91.18323105	92.80919233	60.43840244	Oncofetal suppressor
ASPDH	1.319251216	40.06765935	32.78207548	4.293251221	Oncofetal suppressor
ALDH2	23.33388604	368.829835	316.695189	43.85251924	Oncofetal suppressor
VSNL1	1.555849321	8.093633871	7.020269254	3.845848791	Oncofetal suppressor
AP006621.5	2.594735016	6.260980083	5.79379107	4.014362063	Oncofetal suppressor
POR	7.473396973	26.02203941	36.84311941	10.26487258	Oncofetal suppressor
EHD3	1.979754728	5.892107989	4.167033892	1.091893188	Oncofetal suppressor
HSD17B7	1.761176914	3.260445426	9.764172934	3.114240402	Oncofetal suppressor
FCN2	0	29.392431	14.30098883	0.013820128	Oncofetal suppressor
MTHFD1	3.03872721	66.69060263	63.46334098	20.9485353	Oncofetal suppressor
FAM195A	0.83121914	2.747348165	5.5300262	2.462633728	Oncofetal suppressor
C1QBP	12.28837779	17.3372032	20.63304511	12.63911919	Oncofetal suppressor
ACACB	0.317922244	12.31198544	12.88974465	5.997055905	Oncofetal suppressor
MAP1LC3A	1.912140582	4.277180636	4.708626275	1.092072437	Oncofetal suppressor
LYRM2	3.678358362	3.713018082	24.70103377	3.317934111	Oncofetal suppressor
RGN	12.78331881	66.66126479	70.89608815	16.85529807	Oncofetal suppressor
CD8A	0.015182147	2.682860292	5.563453515	0.528925683	Oncofetal suppressor
TLR4	4.581703918	6.295684139	8.894667701	3.861098041	Oncofetal suppressor
HGD	31.60648176	161.9204213	167.8995936	25.66762293	Oncofetal suppressor
GSTO1	19.10768822	44.6163736	46.32485794	14.05205393	Oncofetal suppressor
CHST13	0.246987818	4.051489648	4.713057808	2.52638729	Oncofetal suppressor
TRPM8	0.026208078	4.804534823	7.770989585	1.735511083	Oncofetal suppressor
ASGR1	9.722449689	55.90070001	59.94756964	26.30291064	Oncofetal suppressor
SAT2	27.87685682	71.5492178	51.90585822	18.99746019	Oncofetal suppressor
PRG4	0.360926234	88.66056038	129.1465145	4.612307463	Oncofetal suppressor
INSIG1	6.620351992	54.90244006	154.8575229	14.65088756	Oncofetal suppressor
SERPINA7	25.43351993	56.94828632	109.2570749	16.1256668	Oncofetal suppressor
SAMD5	1.446361349	5.346553457	2.906374271	0.400055517	Oncofetal suppressor
RNF130	4.850979857	35.10727949	31.002195	18.2071707	Oncofetal suppressor

TTPAL	2.522871299	4.788251582	6.365733406	3.084315394	Oncofetal suppressor
FST	7.106390248	20.83397513	17.40331758	4.928632649	Oncofetal suppressor
CFHR4	0	52.28531111	44.79914254	3.939439704	Oncofetal suppressor
SDHB	15.98426636	44.1682843	52.56984246	19.9664524	Oncofetal suppressor
QDPR	5.073676006	25.55898075	27.74636884	5.398036089	Oncofetal suppressor
FETUB	0.054577718	56.55468354	51.62019288	16.91374667	Oncofetal suppressor
SRD5A1	1.671690403	10.76214362	9.376913349	4.728811663	Oncofetal suppressor
FNDC5	0.91057333	6.566367307	13.53498158	1.24739969	Oncofetal suppressor
AGMO	0.076990035	25.37882721	23.24797105	8.863833715	Oncofetal suppressor
42251	0.070660843	4.114649079	7.927761132	1.446597526	Oncofetal suppressor
CCL2	0.507550795	5.754348356	26.57111363	1.228022015	Oncofetal suppressor
AC008537.2	1.038418742	40.73604902	18.22250535	5.426946847	Oncofetal suppressor
UGT1A9	0	11.3780214	20.50031852	5.426997043	Oncofetal suppressor
ASB13	1.675449044	8.125013083	8.258502239	3.320589326	Oncofetal suppressor
CRHBP	4.285801488	42.59293072	62.88066473	0.606665633	Oncofetal suppressor
SCP2	7.433629807	53.88680525	81.39897695	28.67906101	Oncofetal suppressor
NAT2	0.752033068	43.47211721	28.62053902	0.761622837	Oncofetal suppressor
P2RY13	0.079931477	3.024617117	6.275572095	0.501894631	Oncofetal suppressor
RAMP3	0	4.487648434	5.327260314	0.391989952	Oncofetal suppressor
CFI	13.77251036	183.4337246	242.3882985	21.28868785	Oncofetal suppressor
NDRG2	3.286525618	30.55139374	24.76329866	10.84362167	Oncofetal suppressor
CCR1	0.879758445	4.95365869	14.53969648	2.212080681	Oncofetal suppressor
FOLH1	0.220266194	12.17489122	11.10034476	4.622406195	Oncofetal suppressor
PDK4	22.79767694	201.6975105	71.66558898	14.28441224	Oncofetal suppressor
LDB2	2.115966218	6.218656408	5.630128968	3.208414597	Oncofetal suppressor
GPER	0.22801666	5.571207637	3.045197035	2.501087592	Oncofetal suppressor
ETS1	9.043942838	13.55355543	15.90943192	9.773521889	Oncofetal suppressor
NMRK1	3.13318466	19.2433489	18.84512957	11.31764748	Oncofetal suppressor
FAM176A	2.324843724	13.7700137	18.69674051	0.782791362	Oncofetal suppressor
ACADL	0.737191439	11.50064912	10.50341775	0.464546102	Oncofetal suppressor
IRF1	1.527945177	3.060038751	6.788547434	3.25336682	Oncofetal suppressor
PPP1R3B	10.90357171	9.839302397	64.7044435	19.03561298	Oncofetal suppressor
OLFM2	2.962073617	11.3141091	16.47286822	8.913826212	Oncofetal suppressor
EDNRB	11.15348516	14.80033409	19.99568424	4.830465316	Oncofetal suppressor
PANK1	6.885679401	38.70773125	32.96928319	20.97060188	Oncofetal suppressor
DHRS1	1.330574657	7.154651683	7.753254506	2.87177387	Oncofetal suppressor
CDNF	1.808686058	5.822103585	3.963538722	2.956622098	Oncofetal suppressor
GCH1	3.99131237	11.01781133	19.902476	2.44143722	Oncofetal suppressor
ECM1	1.084927071	15.99497776	12.04178374	1.085443509	Oncofetal suppressor
MAT1A	11.14382347	381.8976695	305.1184662	29.94825752	Oncofetal suppressor
AKR1C1	2.911686229	24.01108971	29.57125932	8.73811038	Oncofetal suppressor
GHR	11.55501677	54.98712211	78.08800079	20.50355154	Oncofetal suppressor
AGFG2	1.915699831	7.247152703	9.414745856	5.436444096	Oncofetal suppressor
COQ9	3.378593007	8.3669753	10.93677547	4.434595741	Oncofetal suppressor

RANBP3L	0.410590625	8.95776922	5.467612195	0.465725558	Oncofetal suppressor
NR1I2	0.010264384	17.28117833	18.21513484	6.765243922	Oncofetal suppressor
CCDC25	10.88865101	17.20451934	17.27655753	11.16201347	Oncofetal suppressor
TMEM176A	7.445850323	119.2208283	141.0782957	24.86373222	Oncofetal suppressor
ACADM	7.029767715	57.27112481	48.37936892	31.09247892	Oncofetal suppressor
HSD17B8	1.426804176	9.407609839	8.633881071	5.044358344	Oncofetal suppressor
AADAT	3.34434535	22.15923658	17.54037948	0.960742256	Oncofetal suppressor
ID1	17.6493629	18.68742319	70.22980165	1.887248816	Oncofetal suppressor
SHMT2	9.975098951	18.18651309	16.92548731	11.15039482	Oncofetal suppressor
GPT	0.360735746	14.22558521	13.19665343	1.372038791	Oncofetal suppressor
RILP	1.066216569	5.368639633	6.731893975	1.151800255	Oncofetal suppressor
HFE2	0	62.48984723	55.2902284	0.509172899	Oncofetal suppressor
IFI6	8.987278167	8.450738367	34.61842295	7.889375567	Oncofetal suppressor
NECAB2	0	3.129540987	5.260025705	1.349590293	Oncofetal suppressor
KLKB1	0.046759779	102.4541235	118.0076103	37.20291278	Oncofetal suppressor
COMMD1	2.088927182	4.593429511	4.805935478	2.425984171	Oncofetal suppressor
SIGLEC7	0.015744769	5.705439321	6.380138034	0.676091038	Oncofetal suppressor
AHSG	111.3924968	680.4558935	952.3389888	115.9723313	Oncofetal suppressor
CYP3A5	1.177519745	104.3450393	126.96879	48.59227875	Oncofetal suppressor
TOLLIP	4.583488413	10.17344022	10.47761361	5.269275297	Oncofetal suppressor
CRYZ	24.93657419	28.71470782	46.74913001	13.20775804	Oncofetal suppressor
PGLYRP2	0	39.59726007	32.07484149	1.767374871	Oncofetal suppressor
SLC17A2	0.188783475	30.62141971	20.94499455	1.625668078	Oncofetal suppressor
CD2	0	6.7084014	10.95150674	3.184975557	Oncofetal suppressor
PLGLB1	1.783911706	14.33712749	13.49410809	6.267719681	Oncofetal suppressor
SLC6A12	0.068032475	6.105266976	10.33811386	0.830737276	Oncofetal suppressor
MRPL41	9.716044771	16.77320946	16.63417287	7.730116394	Oncofetal suppressor
MMAA	1.462294685	3.948195781	5.094409813	1.932328177	Oncofetal suppressor
CD97	0.260854112	5.777534711	10.26565783	2.432192642	Oncofetal suppressor
IL13RA2	0	4.457635289	4.153164884	0.013760631	Oncofetal suppressor
ABCG2	6.351526996	13.74023259	12.79130727	4.68910541	Oncofetal suppressor
KEAP1	4.585680106	7.173770023	7.586327772	4.837791664	Oncofetal suppressor
PRCP	3.908968085	6.41002369	9.326087376	2.3392299	Oncofetal suppressor
ZNF385B	0.977753142	7.433785106	5.381361999	0.349737717	Oncofetal suppressor
SLC25A47	0.092105588	46.10422321	53.09986699	0.808427526	Oncofetal suppressor
ARHGDIB	12.44179838	41.21185954	46.00552815	20.40554977	Oncofetal suppressor
PXMP2	0.525127593	9.808696888	7.394155096	2.858212603	Oncofetal suppressor
PCCB	2.469409877	9.691440613	10.17491052	2.159648134	Oncofetal suppressor
CDHR2	0.38212567	16.1518762	24.42468325	0.142248081	Oncofetal suppressor
SLC25A18	0.81154284	10.23332805	12.01106669	0.730033006	Oncofetal suppressor
THRSP	0.10585474	51.61840441	148.6853446	0.62893352	Oncofetal suppressor
ESYT1	5.965186628	8.190760985	11.6476039	6.195530708	Oncofetal suppressor
TMBIM6	71.09630383	179.8496506	185.6218384	93.56038469	Oncofetal suppressor
AHR	3.094968766	14.7919326	12.76832188	8.511891435	Oncofetal suppressor

ZCCHC6	11.02264846	32.2975697	19.54757216	14.97212488	Oncofetal suppressor
RETSAT	4.776510324	26.28473174	27.99138514	11.43926699	Oncofetal suppressor
PLIN5	0.141535083	14.45354473	17.56195437	6.898663143	Oncofetal suppressor
C1S	5.824541954	277.5095247	298.5908491	169.7913243	Oncofetal suppressor
GLTPD2	2.22712302	4.830788443	6.563496839	0.852997724	Oncofetal suppressor
NFIC	4.57502704	38.3907195	40.6243069	24.72624816	Oncofetal suppressor
CPT2	3.840788574	11.71538993	16.37273999	6.407833577	Oncofetal suppressor
GREM2	1.102666232	6.888434978	8.555246483	0.262917155	Oncofetal suppressor
SLC10A1	31.90905344	175.955074	160.3913188	26.48065258	Oncofetal suppressor
ITIH1	2.629113088	324.5963785	234.5908962	61.29795115	Oncofetal suppressor
F11	0.065086791	74.04206545	69.35167576	37.02412812	Oncofetal suppressor
ACADSB	10.36440032	174.9777703	87.44643322	45.13590014	Oncofetal suppressor
C8G	0.633773094	32.6363043	47.93456831	18.85687568	Oncofetal suppressor
FAHD2A	2.474013269	13.78322435	13.07865189	7.302909115	Oncofetal suppressor
C3	9.543673812	1240.147643	1297.861162	398.8582988	Oncofetal suppressor
CLDN2	1.639972406	5.55540671	10.17907829	0.590536886	Oncofetal suppressor
GADD45A	23.16487812	27.72700956	68.87799283	27.51157889	Oncofetal suppressor
ACOT2	2.324489705	13.17563158	9.550251845	6.971038993	Oncofetal suppressor
C16orf45	0.610284849	4.053480788	5.158431559	0.964639654	Oncofetal suppressor
RCL1	1.627710602	8.900105138	13.3266416	2.903721384	Oncofetal suppressor
CYP2C8	1.740118523	1000.448935	823.9113989	158.2422178	Oncofetal suppressor
ABCA6	0.027249693	39.68633756	42.264951	17.79465883	Oncofetal suppressor
NAGS	0.233584193	5.86188393	4.76532011	1.665402181	Oncofetal suppressor
MAN2B2	2.821222728	6.322236013	6.635302017	3.980589676	Oncofetal suppressor
AR	0.682758501	30.30626725	21.59494248	3.927705608	Oncofetal suppressor
GCAT	2.036408394	9.472752122	8.11430305	2.150193559	Oncofetal suppressor
PION	0.677857994	4.596998422	8.505757113	3.969838021	Oncofetal suppressor
SLC28A1	0.216686536	4.250842227	10.14314206	0.496410305	Oncofetal suppressor
CD3E	0.014565572	5.408457489	7.075976576	1.313849978	Oncofetal suppressor
HMGCS2	35.18906347	910.3392529	753.5104149	276.0688353	Oncofetal suppressor
UCK1	4.273955129	9.223157675	7.357432243	4.952752208	Oncofetal suppressor
DAO	0.024892387	17.60038924	22.46957395	1.734434901	Oncofetal suppressor
C1R	2.031822922	134.334135	133.9104883	62.12624371	Oncofetal suppressor
ADAMTS13	0.640097447	5.918850379	3.221947494	0.424315529	Oncofetal suppressor
PLG	26.36635528	513.6405254	514.8770081	183.2422697	Oncofetal suppressor
GRAMD1C	0.339110673	6.61353197	5.626921431	2.495627242	Oncofetal suppressor
DUSP1	25.01746372	58.49991491	50.36234763	31.40670866	Oncofetal suppressor
AGPAT2	12.39509157	18.82758151	26.99930715	9.594527446	Oncofetal suppressor
IYD	1.142018477	7.008809926	5.159701863	2.472411743	Oncofetal suppressor
GPHN	2.972191511	9.272397649	7.634897659	4.182934106	Oncofetal suppressor
CASP1	0.204043243	5.720402406	7.376667385	2.693150007	Oncofetal suppressor
EMILIN1	2.106329907	4.943355102	5.525241724	2.467848948	Oncofetal suppressor
STAP2	3.326185032	9.625330248	18.71658983	5.112988308	Oncofetal suppressor
SPP2	0.392959572	95.14290348	118.6034703	52.69129551	Oncofetal suppressor

ADI1	10.33057183	149.5511094	133.5556564	57.40280779	Oncofetal suppressor
HBA1	0	28.4578276	11.23004416	0.643107634	Oncofetal suppressor
SLCO1B1	0.179648595	112.5358608	80.3371414	15.81994771	Oncofetal suppressor
SLC25A27	2.011943918	12.1975837	11.44054645	6.630285084	Oncofetal suppressor
LPAR6	1.062962789	7.246303405	6.211249601	3.344194525	Oncofetal suppressor
REEP6	23.88345543	49.76430064	60.92592639	15.59794249	Oncofetal suppressor
FTCD	3.910091918	86.69928631	77.71879784	33.42048168	Oncofetal suppressor
CYP1A1	2.604858645	34.57649134	7.841341117	0.377798432	Oncofetal suppressor
SLC25A25	2.881438255	8.018960856	28.62967584	5.212844401	Oncofetal suppressor
ALDH1L1	0.382860404	23.63221795	15.88181854	1.501992231	Oncofetal suppressor
SLC27A2	12.00452691	100.178346	108.4536312	18.27833197	Oncofetal suppressor
CYP2D6	0.490060055	90.67881559	86.32137068	34.03396535	Oncofetal suppressor
COMT	3.120458718	9.037154473	13.37795796	2.131957515	Oncofetal suppressor
PDSS2	4.50189717	5.80603616	7.769860917	3.807019191	Oncofetal suppressor
BBOX1	0.297009994	22.46316626	17.95539739	1.389343476	Oncofetal suppressor
IFI30	0.856320169	38.18955931	69.23180984	31.56752816	Oncofetal suppressor
APOM	3.967767241	59.01993955	54.18709281	12.33309012	Oncofetal suppressor
MOGAT2	0.223519426	21.18325242	23.97933879	0.518960981	Oncofetal suppressor
CYP2C19	0.011067189	25.36083269	5.891936715	1.416731068	Oncofetal suppressor
APOD	0.075420097	14.88342392	0.474236933	0.325895843	Oncofetal suppressor
RELN	0.502432752	9.782547212	8.139307718	0.086723978	Oncofetal suppressor
AGPAT9	1.250220039	4.369996974	3.805473334	1.896824456	Oncofetal suppressor
MRPL40	7.469759276	21.29332877	17.77246593	12.34313919	Oncofetal suppressor
RASGEF1B	0.794136661	3.943049773	4.891659519	1.703712872	Oncofetal suppressor
APOC1	126.0706224	3627.005476	4090.583909	2075.127971	Oncofetal suppressor
MASP1	8.949693495	16.08334318	19.42156093	4.303123589	Oncofetal suppressor
MSRA	0.252143289	4.789119701	5.488849169	1.128953665	Oncofetal suppressor
MARCO	0.059775596	60.9318337	69.90166046	0.882507209	Oncofetal suppressor
F13B	0.067631817	93.0387672	118.4047209	42.31117779	Oncofetal suppressor
CPB2	0.329723164	599.7722084	497.5644817	312.7257572	Oncofetal suppressor
AMBP	9.612999819	1604.336763	1424.318805	705.3159086	Oncofetal suppressor
SLC16A13	1.190929224	6.590774065	9.651831241	2.744583292	Oncofetal suppressor
C7	0.264372252	86.47293983	72.78904865	5.958898706	Oncofetal suppressor
MSH3	3.328151347	7.438031853	8.19879577	4.908293565	Oncofetal suppressor
KLHL2	3.42351142	7.232458834	6.752049489	4.114371887	Oncofetal suppressor
C9	0.04679147	437.4747936	412.3748566	9.113688006	Oncofetal suppressor
SERPINC1	2.999731324	888.03938	760.0555849	265.857644	Oncofetal suppressor
F8	0.24444316	7.2325563	6.003511329	2.313668951	Oncofetal suppressor
TIMD4	2.26436109	7.587638636	4.59095463	0.143889302	Oncofetal suppressor
IL8	0.133588206	0.561414519	8.416913849	2.17450651	Oncofetal suppressor
CTSF	0.233632588	14.3768918	14.44416333	6.744885554	Oncofetal suppressor
MASP2	2.319225638	38.44084847	30.44169789	6.526637307	Oncofetal suppressor
CXCL10	0.058858324	4.05505154	28.99919687	1.506912301	Oncofetal suppressor
CST7	0.014847487	4.561069422	5.29040478	0.818002945	Oncofetal suppressor

CYP2A7	0.060271334	52.03084404	20.75981622	1.211031654	Oncofetal suppressor
DHRS4	1.767205208	12.73887296	11.7273227	6.125158458	Oncofetal suppressor
OAS1	0.194232468	3.918127721	9.084660484	2.248923548	Oncofetal suppressor
MYLK	2.863670669	8.967210981	7.693916615	5.298441489	Oncofetal suppressor
MRPL16	7.205051885	16.01238323	19.03953741	11.34010533	Oncofetal suppressor
ABCA8	1.103018916	15.75412297	16.02064572	0.410425077	Oncofetal suppressor
SEMA4G	5.354507419	9.70175299	10.41670814	6.431739488	Oncofetal suppressor
STAB1	0.294734112	4.597438732	4.572128196	0.910798353	Oncofetal suppressor
MPND	2.101242154	7.336249448	7.503384557	2.702268894	Oncofetal suppressor
HLA-DMA	0.589120715	10.99689124	15.40231751	7.561538982	Oncofetal suppressor
HLA-DOA	0.024092009	2.612397555	5.535362529	1.087508314	Oncofetal suppressor
UGT1A3	0.005841016	4.041596186	5.019486769	1.012733566	Oncofetal suppressor
PPP1R1A	3.278919283	22.93537341	10.78416661	9.941855963	Oncofetal suppressor
BLOC1S1	3.024962625	7.244834301	8.616698189	3.524332687	Oncofetal suppressor
GNE	7.170059301	25.37342523	24.3531692	9.204015676	Oncofetal suppressor
CYP4F3	0.005466414	63.9216845	56.06533015	21.85660552	Oncofetal suppressor
MT1E	5.880282225	345.3533873	326.6478845	1.212322079	Oncofetal suppressor
LRG1	1.520449379	82.55003272	117.2515244	16.17772481	Oncofetal suppressor
COX14	3.235700257	6.07357294	6.860821458	4.084770277	Oncofetal suppressor
EI24	19.55929555	39.51244166	49.66234169	14.24021891	Oncofetal suppressor
AQP7	0.785830399	7.021306113	5.850148566	2.322012749	Oncofetal suppressor
PID1	10.19193462	15.86577974	16.54134955	6.458358394	Oncofetal suppressor
AC069257.9	1.293944589	4.847285043	6.984338523	3.043613497	Oncofetal suppressor
ART4	4.427934039	12.57826732	12.030332	2.235762128	Oncofetal suppressor
OAS2	0.134466463	3.293808782	7.854528169	1.335914772	Oncofetal suppressor
TMEM192	7.787279969	12.61077578	13.47559523	6.625200201	Oncofetal suppressor
UGT1A1	0.145284005	21.66989013	32.76638698	5.950581217	Oncofetal suppressor
ACP5	0.040900956	8.429232166	17.76275159	3.777733493	Oncofetal suppressor
FXN	1.216847277	5.324715605	4.214704318	2.032687302	Oncofetal suppressor
CPVL	4.684026458	9.691443887	14.00867134	2.210682493	Oncofetal suppressor
ABCB11	0.694295117	23.31354821	22.75550105	5.190811454	Oncofetal suppressor
FMO3	0.099683531	80.07570653	170.7302435	26.52927321	Oncofetal suppressor
C4BPA	0.260211178	301.6223782	456.450112	22.16396297	Oncofetal suppressor
FCER1G	0.701867192	38.83284914	39.40944805	9.00561944	Oncofetal suppressor
CD74	9.770662884	264.5947924	397.1111065	108.9505933	Oncofetal suppressor
SLC27A4	1.384192351	3.855013048	4.656808399	1.719333922	Oncofetal suppressor
FAM149A	0.603346321	10.86781587	9.554767084	5.595840611	Oncofetal suppressor
UGT2B15	0.106370055	247.1146567	406.0374057	159.8014358	Oncofetal suppressor
SERPINA11	6.267932945	168.9290471	224.9708157	37.83703724	Oncofetal suppressor
GLYAT	3.274866408	140.2266563	93.14986959	5.78817944	Oncofetal suppressor
PIR	0.87400393	3.942828181	6.329060495	1.166837987	Oncofetal suppressor
RBL2	8.01350214	19.39076396	15.89675508	9.01231438	Oncofetal suppressor
ACMSD	2.796338879	27.31943126	20.65230103	9.608705417	Oncofetal suppressor
CCDC152	2.048485814	6.193574842	4.431361392	3.025911078	Oncofetal suppressor

ID2	18.01010249	71.84987201	119.5291348	50.53353129	Oncofetal suppressor
MVK	1.064246077	1.916912832	6.243725684	0.84831426	Oncofetal suppressor
AOX1	3.044162676	232.6305242	225.1698827	9.712398294	Oncofetal suppressor
MAP2K3	2.401737001	4.169412259	6.074451801	1.608760749	Oncofetal suppressor
IGSF6	0.503947545	7.330063705	14.60352313	4.94984046	Oncofetal suppressor
TTPA	0.231195692	58.64494264	66.20190025	9.450045844	Oncofetal suppressor
MPEG1	0.062170926	14.70868775	23.92473013	4.387311795	Oncofetal suppressor
FOS	7.567402124	35.4764664	43.29769794	2.453830456	Oncofetal suppressor
PON3	0.177728827	88.27218113	68.62890557	7.476723193	Oncofetal suppressor
PCYT2	1.391225441	6.364238127	7.493439301	3.772379912	Oncofetal suppressor
NNMT	16.11661884	21.02154248	28.27071715	1.234100931	Oncofetal suppressor
CMC2	1.864988139	6.577422151	7.547786983	3.811807259	Oncofetal suppressor
FOLR2	0	9.444319431	11.24939532	1.612882132	Oncofetal suppressor
SLC13A5	2.513509915	25.1510184	29.15214195	2.39391781	Oncofetal suppressor
DSG1	0.007887374	6.602368809	6.116756478	0.920212085	Oncofetal suppressor
NENF	3.104160613	11.76414011	9.55746724	6.851194299	Oncofetal suppressor
PLEK	0.083332304	5.610707868	10.78833902	2.140255674	Oncofetal suppressor
IL32	23.74399528	25.09820936	99.53385518	19.71185016	Oncofetal suppressor
ST3GAL1	12.49852255	25.34868391	23.56438473	6.948229788	Oncofetal suppressor
TYROBP	0.759211696	61.49066618	78.00646773	24.71991011	Oncofetal suppressor
SLC35D1	5.647565577	14.83179169	18.93571496	6.658641367	Oncofetal suppressor
NCF2	0.381988108	3.178701692	4.896090216	1.910415509	Oncofetal suppressor
NGFR	0.136417993	6.21706467	4.877070816	0.943515091	Oncofetal suppressor
MIA3	12.47315681	25.9169038	28.78633147	12.88285211	Oncofetal suppressor
CYBB	0.053072296	13.90595097	21.14839152	4.94744317	Oncofetal suppressor
GRB14	0.302300632	24.66232209	26.03349149	8.297285239	Oncofetal suppressor
FIS1	10.32342862	24.6291828	22.50836217	9.32386644	Oncofetal suppressor
RARRES3	1.626365587	21.47892566	21.61152992	10.79983918	Oncofetal suppressor
ITGAL	0.14569764	4.220298037	5.379610552	3.140697442	Oncofetal suppressor
SLC25A42	4.241204515	10.26274553	11.7171079	4.63641009	Oncofetal suppressor
PLIN4	0.161142413	4.221511822	7.299685763	2.388855137	Oncofetal suppressor
MX1	2.079803397	2.140187665	7.042428753	2.182652492	Oncofetal suppressor
FCGRT	12.71423449	46.42823872	48.8991721	27.43192264	Oncofetal suppressor
BAAT	0.468556717	387.721607	305.3345829	102.1621261	Oncofetal suppressor
SIK1	7.977986773	2.376884355	23.43288437	4.945716201	Oncofetal suppressor
SULT1B1	1.215449054	4.32785853	5.638129166	0.09378653	Oncofetal suppressor
MT1F	2.989697488	60.78175251	30.01921272	0.319809017	Oncofetal suppressor
CLRN3	3.464026934	16.21202328	19.96436248	0.369028596	Oncofetal suppressor
ADAMTSL3	1.762473098	4.285442747	3.720811892	2.321435706	Oncofetal suppressor
BHMT2	0.086789206	130.1868261	123.2732578	71.13944515	Oncofetal suppressor
SFXN2	0.804829709	10.78726254	4.72198348	4.745214541	Oncofetal suppressor
GPR137B	3.120317597	4.481462452	5.223631222	3.220241314	Oncofetal suppressor
EPHX2	7.455111331	51.79342573	43.33371731	10.57462521	Oncofetal suppressor
CDC14B	3.169129821	5.446625041	5.751238322	1.024913714	Oncofetal suppressor

OLFML1	0.156728273	5.333585509	3.880221899	1.146089178	Oncofetal suppressor
TUBE1	4.391938519	20.94270979	17.47100663	2.952875646	Oncofetal suppressor
HLA-DRA	0.366779322	158.8581664	235.2260813	108.1855885	Oncofetal suppressor
GLYATL1	0.461964268	85.88135068	76.37964695	2.307399852	Oncofetal suppressor
ACADS	2.925369822	17.78404688	17.69869333	2.923593697	Oncofetal suppressor
MYCT1	0.478500689	5.586137221	5.113339009	2.879923008	Oncofetal suppressor
GHITM	51.03439842	82.91649392	115.5307645	53.47658076	Oncofetal suppressor
MRPS28	2.803730602	11.14940082	10.01192027	6.180352597	Oncofetal suppressor
CSTA	0	4.589161061	7.588481729	2.085923281	Oncofetal suppressor
HPX	51.32331703	341.6750078	299.8153733	28.30894214	Oncofetal suppressor
TM6SF2	2.162383723	8.061563634	8.738771457	2.736731812	Oncofetal suppressor
ALB	230.7473763	38895.13499	31228.73251	8554.226463	Oncofetal suppressor
OIT3	6.21221359	19.80763692	36.00264868	5.03314435	Oncofetal suppressor
LARP1B	7.761594653	13.34729896	12.87099161	8.446532781	Oncofetal suppressor
B2M	68.24076987	356.3532071	673.4828196	161.3769469	Oncofetal suppressor
SULT1A1	1.176196895	20.47285365	18.9367361	5.119187324	Oncofetal suppressor
SDPR	0.547366005	11.34462942	14.25719418	2.439121966	Oncofetal suppressor
AIMP2	1.134402923	3.618590394	4.70288303	2.70250413	Oncofetal suppressor
GCHFR	4.388990899	13.42672468	8.694475874	3.351096204	Oncofetal suppressor
OAF	4.218182363	44.05841576	46.58100586	20.65529552	Oncofetal suppressor
SLC27A5	0.50296569	103.5579556	95.35250428	15.42827291	Oncofetal suppressor
CLEC2B	0.230310523	6.751864638	9.808117429	3.545360399	Oncofetal suppressor
PTPRC	0.003246306	8.539541518	14.31374967	3.461292931	Oncofetal suppressor
BIN2	0.029057581	6.985074077	9.444195065	2.278913434	Oncofetal suppressor
ENTPD5	5.848413464	29.35426573	31.6901372	17.75496109	Oncofetal suppressor
VIPR1	0.23019843	7.328894694	3.506887202	0.075815893	Oncofetal suppressor
UGP2	42.89974657	95.94301427	136.8780588	53.09564114	Oncofetal suppressor
TRAPPC5	5.707142634	18.67697513	13.40428749	5.959113978	Oncofetal suppressor
IFITM2	35.32861221	50.25710942	61.60336951	34.0329575	Oncofetal suppressor
AGXT	0.579081953	166.512397	153.6240642	100.2775807	Oncofetal suppressor
CNGA1	1.727466323	12.24778892	19.32135742	2.867921524	Oncofetal suppressor
ACADVL	33.33239446	79.0246809	83.85742082	26.69812814	Oncofetal suppressor
UPB1	0.463597474	32.40224488	33.57594896	7.553352613	Oncofetal suppressor
CHDH	4.375852265	18.8867911	14.06895372	8.091605063	Oncofetal suppressor
ALDH5A1	8.419855244	34.88770276	39.48982305	18.65712962	Oncofetal suppressor
GPLD1	0.9782656	10.24112152	15.70028226	6.245079848	Oncofetal suppressor
ARHGEF6	1.653445445	3.852229965	4.406205322	1.769121597	Oncofetal suppressor
ALDH9A1	6.428225695	42.2655655	41.2251942	19.40546019	Oncofetal suppressor
AKR1A1	12.63130554	20.41309945	25.73001133	10.71064753	Oncofetal suppressor
ANXA10	0.106685584	25.29585583	21.81802501	1.531166401	Oncofetal suppressor
ACP2	3.630389511	8.48708798	9.248472191	4.893681765	Oncofetal suppressor
RBP4	447.3315337	3576.098989	3388.309289	1108.645649	Oncofetal suppressor
HSD17B6	0.729304369	254.3633901	297.398987	122.4901381	Oncofetal suppressor
GPT2	9.238038991	16.55659795	23.91912592	2.468480531	Oncofetal suppressor

CPED1	5.130794274	9.99229467	8.331281869	0.900218533	Oncofetal suppressor
CYP7B1	0.580886775	4.845527429	5.089938341	1.657028444	Oncofetal suppressor
RNASE6	0.312342983	8.092879645	9.965448401	3.669380623	Oncofetal suppressor
NAPEPLD	2.49463699	6.201063851	5.338436882	3.604349939	Oncofetal suppressor
CTSL1	55.42524151	69.78044301	108.7696279	42.11112597	Oncofetal suppressor
ITGA9	1.346283354	5.66215447	6.412823771	1.684123711	Oncofetal suppressor
GAMT	6.253813235	62.32440306	39.47503194	9.22309511	Oncofetal suppressor
ISG15	4.525037855	5.241932024	21.60106368	2.674904146	Oncofetal suppressor
SULT1A2	0.837594047	11.38627453	7.064725152	1.701467277	Oncofetal suppressor
PC	2.285440802	15.60584291	12.54283624	3.869437507	Oncofetal suppressor
SLC16A2	8.722151346	27.99621842	25.87670053	11.70044861	Oncofetal suppressor
PCK2	1.990959725	56.66645157	55.85201857	9.021488256	Oncofetal suppressor
F9	0	230.4737019	261.0229299	18.43907761	Oncofetal suppressor
CHP1	27.23797118	50.3628406	60.25490924	29.82349896	Oncofetal suppressor
PLA1A	0.959422751	11.38035145	11.69756915	4.023803557	Oncofetal suppressor
MST1P9	1.408197898	10.90032744	9.485055444	6.131409845	Oncofetal suppressor
SLC22A3	8.111575426	16.6176141	19.84789081	11.19906296	Oncofetal suppressor
C8orf40	5.328430512	22.50618765	17.11851039	10.11708676	Oncofetal suppressor
MCC	3.180275263	4.937148197	4.934381776	1.970825155	Oncofetal suppressor
CERS4	4.67475097	7.825099237	9.692500776	2.357697893	Oncofetal suppressor
PHGDH	9.190705251	19.86970009	13.5802223	5.579767093	Oncofetal suppressor
ADAP2	0.486047328	5.990676851	5.973551637	2.410510326	Oncofetal suppressor
CA5A	0.338794444	13.21771631	11.82230882	0.029674783	Oncofetal suppressor
DMGDH	0.136896942	52.90907227	47.9251993	9.13761536	Oncofetal suppressor
SHBG	0.472464729	11.36381663	10.53100378	0.419835949	Oncofetal suppressor
SCCPDH	13.09099886	40.48320858	37.47109994	23.9123267	Oncofetal suppressor
NR0B2	0.059723887	21.73123495	30.24349861	14.16811737	Oncofetal suppressor
C5orf4	7.741519091	21.56063759	18.04944395	5.951532091	Oncofetal suppressor
PTGR1	38.19318519	99.26059958	95.02059955	39.54361774	Oncofetal suppressor
MT1X	1.082535036	24.52830789	18.93529968	0.252394658	Oncofetal suppressor
ARHGEF26	5.511693302	6.669924785	10.95307064	5.523380512	Oncofetal suppressor
CCDC159	2.522395074	6.340239253	8.048424597	3.223486183	Oncofetal suppressor
KDM8	0.573066042	4.28814519	5.443237329	1.298931525	Oncofetal suppressor
TAT	0.030645081	176.4516954	220.2104298	15.40620121	Oncofetal suppressor
RP11-676J12.7	1.24610249	5.122697672	3.247109275	0.873162398	Oncofetal suppressor
CYP1A2	0	347.4620069	138.6538707	0.500315973	Oncofetal suppressor
SLC25A20	8.472079609	36.8496672	40.83350659	13.332068	Oncofetal suppressor
THNSL1	1.133669115	7.430892095	6.861029641	3.874831021	Oncofetal suppressor
ADHFE1	3.097914472	16.68477162	15.56714521	2.348965057	Oncofetal suppressor
TRIM55	4.843395825	5.049166282	13.12195846	1.482447689	Oncofetal suppressor
AGMAT	1.629363202	31.16689442	29.48186984	8.708266884	Oncofetal suppressor
ASS1	9.377059162	517.7135084	386.6372457	30.08834363	Oncofetal suppressor
HAO2	1.633144446	139.9918803	74.33027834	6.472833798	Oncofetal suppressor
HAAO	4.011762611	59.61336481	40.03980903	13.22295707	Oncofetal suppressor

HSD17B13	0.887132691	322.1493248	279.8857691	17.90356295	Oncofetal suppressor
GFRA1	0.019457904	6.806403439	4.981397153	0.099093166	Oncofetal suppressor
SMPD1	6.538335398	10.54964618	12.83260782	4.351554983	Oncofetal suppressor
CAT	11.80727867	220.9851174	237.8280507	114.8755095	Oncofetal suppressor
RARRES2	1.331167329	182.6707646	191.4360125	64.47762847	Oncofetal suppressor
IFI44	0.059156713	5.756136231	11.87560716	2.228691688	Oncofetal suppressor
DNAJC25	9.968178425	18.2323486	19.7879345	8.828147032	Oncofetal suppressor
USE1	4.161582531	12.03136869	9.825448138	4.831507003	Oncofetal suppressor
TNFAIP8L1	5.802217873	16.50418743	15.55246981	8.017631809	Oncofetal suppressor
ODZ1	0.517163663	4.632070013	5.054012259	0.215142177	Oncofetal suppressor
DECR1	7.012357518	74.89056512	72.01708702	22.67464814	Oncofetal suppressor
ANGPTL3	8.556812225	340.312937	421.4138237	74.02267811	Oncofetal suppressor
UCP2	1.151746659	4.573709761	7.748762021	3.373152853	Oncofetal suppressor
NKG7	0.183885944	4.013545949	5.489331836	0.514674264	Oncofetal suppressor
ALDH4A1	0.067411698	11.51031648	16.43191504	3.537971385	Oncofetal suppressor
MS4A4A	0.060562117	3.973747248	8.008568001	1.115170776	Oncofetal suppressor
MS4A6A	0.004456402	17.91307921	21.73852227	3.047332541	Oncofetal suppressor
C21orf33	6.556363673	15.39969542	18.43389578	9.055814659	Oncofetal suppressor
PLIN1	0.64731445	7.52173926	7.97793926	1.61461456	Oncofetal suppressor
LDHD	0.585339666	22.65996099	19.23716397	1.517577716	Oncofetal suppressor
PDE3B	2.163503093	5.996186733	8.182661601	3.740888151	Oncofetal suppressor
IGF1	1.135455449	41.37598377	23.69632174	2.871896665	Oncofetal suppressor
DDT	0.614396288	9.056384915	8.188845733	3.522784403	Oncofetal suppressor
NPC1L1	0.504791935	4.598918416	7.413582326	0.741281958	Oncofetal suppressor
CYP2B6	0.084440004	192.5118171	175.9660806	4.659313431	Oncofetal suppressor
GLYCTK	1.72854113	17.23597701	14.11354632	7.151715592	Oncofetal suppressor
MACROD1	2.280428981	9.817554235	7.139580984	2.002912908	Oncofetal suppressor
CNDP2	4.534586142	13.83125735	17.27888511	8.40963102	Oncofetal suppressor
RBP1	1.61242566	6.095638544	7.752353637	2.102828841	Oncofetal suppressor
SLC7A2	20.07904245	51.78676252	65.63763107	17.59413947	Oncofetal suppressor
CDH5	0.44938493	4.093376003	5.240339175	3.055372616	Oncofetal suppressor
ADH4	1.22714206	1334.342295	1619.559084	74.93227502	Oncofetal suppressor
GDF2	0.042793893	10.95504794	8.112425511	0.161801142	Oncofetal suppressor
TMPRSS6	4.09644416	17.55955208	20.73133632	7.372387988	Oncofetal suppressor
CYP4A11	0.042595875	318.0419581	237.1030942	36.35183813	Oncofetal suppressor
ISOC2	5.086596696	19.78166579	23.48312002	13.23363438	Oncofetal suppressor
CYP26A1	0.333396283	22.7870818	12.46178671	0.036015753	Oncofetal suppressor
MBL2	1.01752502	15.4967944	63.51642208	0.827534831	Oncofetal suppressor
CRYL1	17.65813882	64.31107131	75.38080178	23.21336447	Oncofetal suppressor
UROD	7.165239874	13.92103489	16.38167697	8.548276389	Oncofetal suppressor
ZNHIT1	4.029677979	12.46311637	11.0198787	4.243493981	Oncofetal suppressor
DCXR	5.190765693	204.2018341	213.5370594	25.45690739	Oncofetal suppressor
ZEB2	2.49468987	4.363510598	4.108012806	2.615484081	Oncofetal suppressor
METTL7B	5.116171847	45.77653204	38.46071788	8.364544999	Oncofetal suppressor

C20orf3	17.72441426	34.31033535	46.49844527	22.91272956	Oncofetal suppressor
CYP3A43	0.021001008	17.01321493	13.38871172	1.752549813	Oncofetal suppressor
CYP4A22	0.037611611	249.8080765	133.1098012	20.59211704	Oncofetal suppressor
F12	0.099733931	39.3308219	38.03139413	9.284445512	Oncofetal suppressor
NR1I3	0.250677689	24.8216288	21.54202733	1.692495192	Oncofetal suppressor
NPL	0.244244888	6.361553635	7.283400189	1.956388362	Oncofetal suppressor
ACSM5	0	71.55753182	39.29478756	2.647937129	Oncofetal suppressor
C6	1.4364685	189.5097588	173.0105453	24.02256033	Oncofetal suppressor
FPGS	2.225028202	5.127421573	4.99553481	3.295296518	Oncofetal suppressor
PROS1	44.82270043	98.53546142	107.6911914	41.06847808	Oncofetal suppressor
GPX3	47.61043599	83.429072	129.9801011	31.16119863	Oncofetal suppressor
TFR2	0.114500066	86.40188217	75.28822628	15.70873457	Oncofetal suppressor
SOWAHB	2.500690887	5.265886031	5.581575738	2.520131715	Oncofetal suppressor
SURF1	2.273916633	7.518548882	9.389730687	3.252666286	Oncofetal suppressor
HTATIP2	4.551707556	8.664956906	14.14831968	7.215608642	Oncofetal suppressor
PBLD	7.587653775	93.81040805	97.16745032	31.28482516	Oncofetal suppressor
FBXO8	3.921321657	5.781667259	10.02485746	3.455749935	Oncofetal suppressor
SERPINA10	1.362856153	106.3197815	120.1656908	68.3398221	Oncofetal suppressor
APRT	4.541453409	6.909427411	7.951054993	2.779784894	Oncofetal suppressor
PPAP2B	9.035647913	26.74601298	23.34602609	13.64667262	Oncofetal suppressor
C8B	10.72169176	231.9513294	226.0536492	144.2267748	Oncofetal suppressor
OTC	0.496571152	96.99316469	137.0329697	27.10384225	Oncofetal suppressor
SKAP1	0.146007763	9.393584884	11.3595154	1.875831794	Oncofetal suppressor
SLC46A3	2.376934791	17.0084916	27.55177405	1.499158096	Oncofetal suppressor
CXCL9	0	13.1062464	54.00324354	4.662774839	Oncofetal suppressor
CLEC4M	0.0070378	24.01261334	13.35464774	0	Oncofetal suppressor
SDS	0	23.08020231	59.52152504	1.1365761	Oncofetal suppressor
SULT1E1	13.73025209	45.28463173	20.65105616	1.534382056	Oncofetal suppressor
PEMT	0.503702434	11.55753212	10.54811537	0.805318189	Oncofetal suppressor
SLCO1B3	0.04066661	15.10575259	12.20171726	0.293538028	Oncofetal suppressor
HGFAC	0.019475547	29.11525223	19.35564795	0.205128508	Oncofetal suppressor
HGF	0.903265225	4.712122734	6.67291474	0.958164958	Oncofetal suppressor
SAMSN1	0.205895827	2.652297497	5.380644963	1.597184946	Oncofetal suppressor
MS4A7	0.031757504	13.51122253	19.25004205	2.686209412	Oncofetal suppressor
CPEB3	1.898680121	4.574299274	8.407821581	2.148398698	Oncofetal suppressor
ST3GAL6	5.676542724	10.61326257	9.325021402	1.377074166	Oncofetal suppressor
AKR7A3	0.371447652	34.11401804	44.58025923	1.007519623	Oncofetal suppressor
CD14	0.051249809	75.62857369	75.7147314	12.62289119	Oncofetal suppressor
CA14	2.307426244	4.656636865	3.356435294	0.58974134	Oncofetal suppressor
IGJ	0	24.03536505	86.43439619	11.05420997	Oncofetal suppressor
ACOX2	1.399064946	47.31875578	41.2637541	10.11764767	Oncofetal suppressor
HADHB	9.929315176	38.68641314	44.11619761	21.93627744	Oncofetal suppressor
GLS2	0.448714298	50.38313442	42.49806814	2.792717067	Oncofetal suppressor
ALDH3A2	11.13630461	41.71478361	53.51064748	27.4073816	Oncofetal suppressor

PRKAG2	4.142243553	5.28019647	7.80725498	2.221845896	Oncofetal suppressor
CD52	0.103376619	4.889042069	5.200206623	0.765768092	Oncofetal suppressor
ALDH1B1	2.382425989	44.5203202	82.69665345	24.96547437	Oncofetal suppressor
TEF	1.634136755	4.851932748	5.864029731	3.435704217	Oncofetal suppressor
TMEM176B	5.572613211	227.0451024	182.6984874	45.95657418	Oncofetal suppressor
CD82	2.554544165	3.911014188	4.259621017	2.152483156	Oncofetal suppressor
PHLPP1	4.602050447	8.833385179	6.708779958	3.976291254	Oncofetal suppressor
NT5DC1	7.755269476	11.01842666	12.47199072	6.858025297	Oncofetal suppressor
AMT	2.36140472	7.345305974	6.142401691	4.204163995	Oncofetal suppressor
C1QC	0	71.01277105	115.05832	14.93328444	Oncofetal suppressor
MAOB	5.047902111	107.1351869	101.0584651	67.32243121	Oncofetal suppressor
ALAD	7.892286432	67.67227978	57.72277648	29.0569277	Oncofetal suppressor
DHODH	1.024724507	7.616656077	7.351745789	1.909536505	Oncofetal suppressor
TSKU	9.395968391	16.94751913	17.31873634	1.781966773	Oncofetal suppressor
PALM3	3.623115264	34.0757618	43.6344104	9.276626061	Oncofetal suppressor
C1QA	0	58.22851805	78.54284077	12.36593936	Oncofetal suppressor
LILRB5	0.01825571	5.482148534	7.021369556	0.48070108	Oncofetal suppressor
GCDH	1.603662941	40.86154222	28.74276463	4.76968801	Oncofetal suppressor
IGFBP1	0.334135819	486.5286717	316.0175787	78.45387915	Oncofetal suppressor
AGXT2	4.898189693	20.62283354	15.85991244	11.96564666	Oncofetal suppressor
CCDC53	4.329362243	8.306375852	9.836953276	4.185025617	Oncofetal suppressor
C1orf162	0.553723288	7.773786614	8.971067643	1.269090127	Oncofetal suppressor
CD53	0.006625798	9.406361048	13.88753654	3.210202086	Oncofetal suppressor
CFHR3	0	93.13251615	119.1274676	2.818691263	Oncofetal suppressor
GPX1	24.67058407	49.63571563	66.3186362	16.42387833	Oncofetal suppressor
GNMT	6.208520927	233.2264779	75.12583802	14.83999362	Oncofetal suppressor
CYP2E1	0.173660502	902.6734601	911.3939977	4.274488877	Oncofetal suppressor
COX6A1	5.095869577	10.81652711	9.589784855	4.52823059	Oncofetal suppressor
IDH1	29.57889211	89.08620843	103.4566581	42.88224269	Oncofetal suppressor
FAS	1.310263616	6.156559816	10.1130481	2.487483277	Oncofetal suppressor
MFSD2A	0.245894044	6.936049561	25.71137191	0.112238708	Oncofetal suppressor
IFIT1	1.668280784	15.1207483	23.72480169	7.419505346	Oncofetal suppressor
BTD	3.597429004	10.25114436	10.08998017	2.953906742	Oncofetal suppressor
F2	12.60604032	331.2443456	284.641839	70.32702484	Oncofetal suppressor
GLUD1	39.17170374	127.2494394	172.9933538	65.01856244	Oncofetal suppressor
FGL1	1.047385877	194.0014652	163.1049498	65.20128765	Oncofetal suppressor
APOA5	1.823988472	148.0698975	92.99749909	7.388990363	Oncofetal suppressor
PNPO	0.714298526	10.32800534	11.55237227	6.960349505	Oncofetal suppressor
APOC3	203.1521955	3717.906921	2229.240451	547.3813229	Oncofetal suppressor
DHRS4L2	1.765275111	11.70053333	12.63910666	4.379351723	Oncofetal suppressor
ACSM2A	0.921667482	147.5081251	155.5325461	46.42324514	Oncofetal suppressor
ABCC2	2.047294987	33.69535014	37.88603757	7.604267922	Oncofetal suppressor
CYB5A	4.80308654	48.48356605	48.93818868	20.35300541	Oncofetal suppressor
COQ5	6.545605478	9.597817372	11.05815601	6.092539947	Oncofetal suppressor

SPI1	0.013347668	4.150094395	6.649067003	2.173674275	Oncofetal suppressor
IL33	0.341786206	11.70362007	15.3841936	2.305322984	Oncofetal suppressor
GPD1	4.778018723	21.88109015	30.24935351	1.715531108	Oncofetal suppressor
HSD11B1	0	206.5195695	163.6232346	10.65954767	Oncofetal suppressor
PEBP1	41.82787271	405.7514571	325.7287887	114.9033275	Oncofetal suppressor
NIT2	6.272167917	14.16682113	17.17644644	3.653026358	Oncofetal suppressor
ASPG	0.091915153	9.239577187	7.554304435	0.656688135	Oncofetal suppressor
ACAA1	13.26491221	41.44600286	43.10072265	5.843817663	Oncofetal suppressor
EGR1	9.76428078	49.3914058	59.58027563	6.132031976	Oncofetal suppressor
CFHR1	0.006250866	534.057795	511.1055367	181.3684591	Oncofetal suppressor
TFB2M	5.865480379	6.484902165	11.40124666	4.225541379	Oncofetal suppressor
SDHD	14.11342314	51.36186412	52.71024292	24.89010816	Oncofetal suppressor
GMFG	0.555746487	22.79060922	28.4040108	10.66785484	Oncofetal suppressor
KIAA0922	3.406143066	7.208381916	9.844891235	5.078338046	Oncofetal suppressor
CYP2C9	0.022740716	666.8888131	685.3585523	65.28190327	Oncofetal suppressor
PAH	0.354203533	205.2233993	210.127046	41.94219692	Oncofetal suppressor
CD5L	0	22.05844513	37.33972559	0.300638417	Oncofetal suppressor
COLEC10	0.388612963	27.42210077	23.99670959	0.602913407	Oncofetal suppressor
PON1	0.055454469	177.382332	197.3415968	9.652309145	Oncofetal suppressor
ARSA	1.440530574	4.048477468	4.498876646	2.084855268	Oncofetal suppressor
HLA-A	4.030353032	40.59740685	84.88831699	35.01530233	Oncofetal suppressor
SRGN	0.067908997	22.48157869	62.12169809	9.335054344	Oncofetal suppressor
MAFB	1.058096761	9.667503252	14.7849942	5.512915257	Oncofetal suppressor
BDH2	4.775510017	9.647386535	8.709008401	3.035775045	Oncofetal suppressor
LDLR	6.077523812	3.695110326	18.76693336	3.067183734	Oncofetal suppressor
RBP5	2.057607886	65.41364809	43.67669804	11.09750182	Oncofetal suppressor
ANG	8.082120421	181.5628612	151.171086	24.39321123	Oncofetal suppressor
CMBL	6.293431567	72.10649339	64.53529874	42.91866377	Oncofetal suppressor
FBP1	10.7369227	377.2714361	451.0012238	55.49429947	Oncofetal suppressor
RGS1	0.211298094	2.220672497	9.523015842	2.43475937	Oncofetal suppressor
CCL4	0	4.84681261	25.4269759	0.939271395	Oncofetal suppressor
MRPL23	2.176816241	5.031875087	3.910767634	1.902796965	Oncofetal suppressor
NR1H4	0.027935589	32.72578189	42.59828172	20.71284421	Oncofetal suppressor
TTR	808.9448762	2257.76434	1640.025482	155.8128244	Oncofetal suppressor
ARSD	1.453490813	7.07073159	6.186289743	2.33932689	Oncofetal suppressor
PNPLA4	2.94140752	4.70349064	4.715665522	2.165301914	Oncofetal suppressor
KCND3	7.630226001	11.78175882	11.25323647	0.125205469	Oncofetal suppressor
HPD	8.707409045	524.6610341	680.3442606	3.115602192	Oncofetal suppressor
GRINA	22.19578828	37.34453241	37.26792698	19.2403684	Oncofetal suppressor
HLA-DPA1	1.282804262	14.73054303	6.669799485	5.286179539	Oncofetal suppressor
TTC36	0.191589968	36.26424768	23.09975843	0.199576886	Oncofetal suppressor
CTSZ	16.7893166	90.1144414	139.1792103	49.70100993	Oncofetal suppressor
GIMAP7	0.01099376	25.54231312	20.90740615	6.252826466	Oncofetal suppressor
LY86	0	2.766737701	5.876312801	0.911025507	Oncofetal suppressor

MYO1B	23.32328693	42.18966324	42.69803744	25.65103574	Oncofetal suppressor
FOSB	1.111128469	5.140950676	52.63936586	1.013512842	Oncofetal suppressor
CYP3A4	0.690408136	1416.175438	427.0250361	21.72218851	Oncofetal suppressor
AZGP1	0.3683541	562.3261255	482.6606824	207.7398197	Oncofetal suppressor
HMGCL	4.488426753	21.77366055	20.25930466	5.382449921	Oncofetal suppressor
C5	24.1695712	130.1536379	141.4047374	75.71106911	Oncofetal suppressor
APLNR	1.312015462	6.990998924	6.159457709	1.244484066	Oncofetal suppressor
CCL5	0.138173743	22.83030941	29.35995074	1.86580941	Oncofetal suppressor
CCL16	0.146118124	60.97021323	55.67562385	16.57391588	Oncofetal suppressor
MRPL54	4.706319128	13.07642784	14.48632306	5.287931844	Oncofetal suppressor
CLEC4G	0	20.66699452	24.14996619	0	Oncofetal suppressor
SLC25A11	3.662862199	5.871086698	6.339904117	2.439513996	Oncofetal suppressor
GPX2	1.825386117	5.224641904	26.01386629	2.767031159	Oncofetal suppressor
HPR	3.324622939	115.8534717	152.8214278	8.080842804	Oncofetal suppressor
POLRMT	3.07413307	4.69263425	4.767752097	2.767407797	Oncofetal suppressor
GSS	3.308020059	6.217224727	6.85470486	4.263238879	Oncofetal suppressor
PFKFB1	0.09393308	11.08604917	12.65668782	2.239653065	Oncofetal suppressor
HLA-DPB1	6.52775827	30.41919801	16.55059217	14.7601666	Oncofetal suppressor
FBXO31	3.497696454	6.323692662	6.00328433	1.414411517	Oncofetal suppressor
LCP2	0.070654408	5.504474995	8.880074846	2.175283484	Oncofetal suppressor
CXCL2	14.6788625	22.01667427	24.39904055	0.705442205	Oncofetal suppressor
DAK	0.807852195	12.0090233	12.68271195	6.649733729	Oncofetal suppressor
HBA2	0	26.2442035	10.70659846	0.365942769	Oncofetal suppressor
UGT2B7	3.45968132	496.1950017	411.6227797	35.86502953	Oncofetal suppressor
ESRP2	3.398054066	7.325194235	7.682389828	4.207087295	Oncofetal suppressor
CPS1	8.971868274	300.2113055	413.2042483	1.816719543	Oncofetal suppressor
NUGGC	1.34679918	12.32989531	9.99157866	2.154234089	Oncofetal suppressor
RPS17	2.269388736	31.55883496	4.657970762	2.844501029	Oncofetal suppressor
DDX58	2.73784815	5.442391686	6.912755872	3.973933363	Oncofetal suppressor
CYP2J2	0.480283921	73.51553228	67.88537505	6.143712264	Oncofetal suppressor
C10orf57	5.590554535	10.39871151	12.30695318	5.720656347	Oncofetal suppressor
DMRTA1	0.377894983	5.662142582	5.12953235	3.129748582	Oncofetal suppressor
GIMAP8	0.316822089	7.726634826	8.697244569	2.775457496	Oncofetal suppressor
CD81	9.945922962	30.49022373	29.79223462	7.035370995	Oncofetal suppressor
AGTR1	0.686835006	16.08579284	14.22394661	5.127287175	Oncofetal suppressor
DNAJC16	3.813652324	10.28239216	10.33331757	6.064682861	Oncofetal suppressor
SEPP1	104.986219	178.5587397	189.1992663	80.48707901	Oncofetal suppressor
HSD17B4	25.66640428	72.97801058	90.87227061	46.80702451	Oncofetal suppressor
TGFBR2	12.09975484	19.32617045	19.73740151	11.8866053	Oncofetal suppressor
HRG	0.403795314	863.6926902	881.1460408	540.1912727	Oncofetal suppressor
LIPC	1.701495011	47.21163135	32.17983434	14.61944467	Oncofetal suppressor
PCTP	3.53892878	10.48121914	12.31167589	4.376021158	Oncofetal suppressor
HOGA1	0.029959852	6.120779573	4.925937334	1.877151804	Oncofetal suppressor
WAS	0.284166306	4.695943304	5.899343087	1.67270558	Oncofetal suppressor

RAB26	0.28259081	3.536536475	5.566888497	1.634113617	Oncofetal suppressor
UQCRQ	5.815938105	18.53490989	26.54293159	14.77465518	Oncofetal suppressor
GBP1	1.966667407	17.69559321	38.29122466	12.0748547	Oncofetal suppressor
GSTA2	124.7291445	646.2455828	87.3061283	119.6699054	Oncofetal suppressor
ARMC6	0.762510846	5.043232559	5.984305722	1.89497476	Oncofetal suppressor
FCGR2B	0.019737225	4.812274219	4.72156127	0.25585805	Oncofetal suppressor
NAT1	1.344696591	4.704899436	5.083559084	2.58089248	Oncofetal suppressor
HP	15.01142723	2388.579536	1731.592753	211.9705717	Oncofetal suppressor
CTSO	3.145867811	30.82411796	39.26159214	22.04151271	Oncofetal suppressor
CES2	4.796546887	75.44338964	76.43738233	22.58835824	Oncofetal suppressor
RPP25L	1.315063117	5.61922001	3.410093923	2.78660344	Oncofetal suppressor
LCAT	3.640094707	36.31117342	27.10773771	3.077065869	Oncofetal suppressor
ADH1B	0	669.6645923	656.4536399	134.2763291	Oncofetal suppressor
UGT2B10	0.700776772	324.6718563	410.6183762	23.92746453	Oncofetal suppressor
IVD	3.085938045	22.86212913	18.85533222	7.131861179	Oncofetal suppressor
PPIF	15.51740052	26.15872394	46.54760167	16.25811895	Oncofetal suppressor
NQO2	3.134292522	13.3134576	27.76051123	5.778653514	Oncofetal suppressor
DNAJC12	0.06308698	14.45267203	10.0604244	1.507838264	Oncofetal suppressor
CD83	0.39675417	1.999958146	8.393969543	1.946814685	Oncofetal suppressor
AIG1	8.307946162	45.71316827	46.48910341	28.91632745	Oncofetal suppressor
NDUFB7	18.73418011	55.37938632	44.58044693	19.72786162	Oncofetal suppressor
FCN1	0	3.074274698	8.011834299	0.434968632	Oncofetal suppressor
RABEPK	2.679619935	4.86173546	4.566893864	1.052621532	Oncofetal suppressor
SLC22A1	0.071668665	218.060474	169.3685994	16.0746195	Oncofetal suppressor
G0S2	15.68964448	20.32968425	49.48371953	3.901377313	Oncofetal suppressor
BTN3A2	2.651215778	5.274468557	12.78382315	4.713844508	Oncofetal suppressor
ABCB4	0.047906623	21.80022209	46.34625143	7.305991784	Oncofetal suppressor
AFM	0.124566195	375.2397452	519.5655033	163.2945623	Oncofetal suppressor
SLC25A15	2.837420319	13.29333793	28.17320725	8.783676204	Oncofetal suppressor
DCN	1.645089782	44.62410942	45.8096208	5.713418189	Oncofetal suppressor
RTP3	0.041811242	19.76509229	26.62658818	1.490523245	Oncofetal suppressor
KCNN2	1.503389958	6.528805251	2.233193975	0.268848587	Oncofetal suppressor
CTBS	5.157897233	6.380637009	10.45865618	5.149337534	Oncofetal suppressor
SC5DL	29.58856573	37.97873268	61.66171349	23.83401165	Oncofetal suppressor
FYB	0.15392706	12.73618565	20.11363942	3.807381693	Oncofetal suppressor
CYP8B1	1.212942526	71.6614163	65.30048589	3.171070028	Oncofetal suppressor
IDNK	1.344096121	14.15453736	13.72778922	5.175502534	Oncofetal suppressor
RHOB	26.91599598	43.53227438	124.7042398	22.95165118	Oncofetal suppressor
DGCR6L	5.647706072	26.55091749	29.9113629	12.54461597	Oncofetal suppressor
ABCA9	0.023393092	8.983955688	8.139219461	1.849824224	Oncofetal suppressor
HS3ST3B1	4.301704505	16.46684997	11.46089114	5.411304789	Oncofetal suppressor
ISOC1	6.399578921	19.880127	28.29461615	13.8190623	Oncofetal suppressor
GAS2	0.383451388	5.823194465	7.588736582	1.312434342	Oncofetal suppressor
CYB5D2	3.272773589	7.466950555	5.523701395	3.197816793	Oncofetal suppressor

ARG1	0.351352741	305.78771	329.6939716	75.02670121	Oncofetal suppressor
CP	0.380405799	247.5322797	336.6870553	96.83473914	Oncofetal suppressor
DBH	0.494576017	8.693911761	5.375072874	2.08852927	Oncofetal suppressor
GCGR	0.515516103	11.98878798	10.64437469	4.356214207	Oncofetal suppressor
STAB2	0.01932667	5.804032641	6.315140934	0.07949698	Oncofetal suppressor
PGM1	21.86292432	52.03193623	57.03066517	17.75367301	Oncofetal suppressor
C4BPB	2.136211568	43.37017034	71.37555849	31.59856451	Oncofetal suppressor
SEC14L2	2.42858447	29.96683626	29.16445073	3.185505378	Oncofetal suppressor
NR4A2	0.233210714	1.982821289	7.777104481	0.953198616	Oncofetal suppressor
ALPL	0.45355279	8.949740816	10.1081238	2.843245922	Oncofetal suppressor
CD86	0	2.869565797	5.467689138	1.050245078	Oncofetal suppressor
LYRM1	6.53322478	20.36772942	19.52532752	11.82569425	Oncofetal suppressor
RP11-115C10.1	0.568237149	27.2007682	17.58729819	8.379035253	Oncofetal suppressor
AGXT2L1	0.065530264	20.8494987	69.59725285	1.797567589	Oncofetal suppressor
APOL1	1.086917043	50.100074	55.37755501	29.1717875	Oncofetal suppressor
PMPCA	5.036137147	6.752719905	9.753099511	5.27377982	Oncofetal suppressor
CUX2	0.019852279	18.45702453	8.344690535	4.000615325	Oncofetal suppressor
ABHD6	3.401063207	11.44021289	16.06882522	2.31914755	Oncofetal suppressor
DNASE1L3	0.06579493	47.42263631	45.87020968	0.646793382	Oncofetal suppressor
C4orf34	14.78705126	32.85897196	30.93447678	17.65822348	Oncofetal suppressor
SERPINA6	0.618832347	169.3328943	192.6628842	78.83125353	Oncofetal suppressor
SQSTM1	6.695085163	11.75720872	15.27390795	8.892693046	Oncofetal suppressor
HACL1	4.086924894	11.55796785	12.66156398	6.275854734	Oncofetal suppressor
PIK3C2G	0.321716437	6.521890638	5.651834247	0.36642821	Oncofetal suppressor
ADH6	0.037650069	166.1943387	140.0500261	47.76439869	Oncofetal suppressor
FH	16.85730417	71.4733136	90.95939647	32.61161684	Oncofetal suppressor
C19orf66	1.812740974	15.159755	18.08265297	4.532979425	Oncofetal suppressor
ACO1	19.70184396	36.68224862	44.82563072	18.05019567	Oncofetal suppressor
CXCL14	7.362952321	29.68021346	31.16865809	0.306503924	Oncofetal suppressor
LECT2	0.095228708	20.10840676	65.17521144	7.221655211	Oncofetal suppressor
AKR1C4	0.057744538	140.5435259	128.0511886	18.92441425	Oncofetal suppressor
SLC25A13	7.42458292	38.26066716	45.37876582	12.95255543	Oncofetal suppressor
ST7	2.481906158	4.812663136	4.415139567	2.375848598	Oncofetal suppressor
C10orf125	2.47116256	19.33054784	17.00755948	5.936167832	Oncofetal suppressor
EVI2B	0.119955082	10.0670472	14.44287714	3.22879399	Oncofetal suppressor
RND1	2.882827132	4.770734837	8.261920598	2.512560724	Oncofetal suppressor
SHC2	5.573174112	10.84140966	8.632120891	2.632856397	Oncofetal suppressor
STEAP4	0.019352716	7.914711145	8.082156987	0.964295281	Oncofetal suppressor
FGL2	0.771260908	15.6765944	25.26703808	4.909745441	Oncofetal suppressor
LST1	0.535713375	7.539946827	8.284656134	1.531317119	Oncofetal suppressor
SLC45A3	0.839231932	4.855793841	3.439847516	2.28462163	Oncofetal suppressor
GOT2	10.97393283	88.6800989	96.35937416	35.623797	Oncofetal suppressor
IL1RN	0.118743414	3.01026665	6.79689705	1.291910274	Oncofetal suppressor
HSD17B10	4.941216975	37.72905347	31.2171561	21.21089621	Oncofetal suppressor

HBB	0	45.35124927	20.70006901	0.669184564	Oncofetal suppressor
MYRIP	1.020562436	5.593157528	4.741458507	2.652052344	Oncofetal suppressor
METTL7A	11.16692464	180.9184401	139.7770201	54.83822874	Oncofetal suppressor
BDH1	1.289590263	22.58437335	23.19903834	10.20763688	Oncofetal suppressor
CLU	12.19275796	407.0192632	403.7391844	233.9172996	Oncofetal suppressor
BCKDK	3.378023793	4.329957606	6.325139685	2.849591764	Oncofetal suppressor
MYO1F	0.30307961	4.756376377	6.40794475	1.069920543	Oncofetal suppressor
HCST	0.05447007	2.964813036	5.137566772	0.794371116	Oncofetal suppressor
LPA	0.089359451	23.99561108	10.26855436	3.51997796	Oncofetal suppressor
AMDHD1	1.379738352	103.3339258	67.93713492	10.54986027	Oncofetal suppressor
NLN	2.934928267	5.776003376	5.785857913	2.062126372	Oncofetal suppressor
C5orf27	0.168228103	20.94245077	21.30660327	0.359828437	Oncofetal suppressor
MSMO1	78.08391882	85.29129078	158.7119282	23.3728319	Oncofetal suppressor
APOA1	966.5229071	1785.349792	1772.45781	736.8124022	Oncofetal suppressor
DPYS	0.473558024	62.84475749	93.49989768	35.18321664	Oncofetal suppressor
AKR1D1	7.991848307	174.6224725	155.7660604	3.250171179	Oncofetal suppressor
PRADC1	3.012868484	7.346695339	8.886128284	5.152402848	Oncofetal suppressor
ENO3	0.530835706	5.982588762	20.95772928	1.563941271	Oncofetal suppressor
C1orf168	4.565962723	9.417834779	11.01526745	2.453354525	Oncofetal suppressor
BCKDHB	3.113737328	15.10960984	12.3753321	7.194622945	Oncofetal suppressor
HAO1	0	169.4692306	189.7519634	32.66189985	Oncofetal suppressor
STARD10	10.03452378	42.22748773	43.4250815	5.123223677	Oncofetal suppressor
TMEM205	12.50731651	32.15094878	37.79917917	12.3709244	Oncofetal suppressor
USP18	1.225806837	4.10083793	7.005441369	2.711458385	Oncofetal suppressor
DUSP10	2.635236582	16.81558313	10.05264696	4.727430412	Oncofetal suppressor
FAM210B	13.1326166	59.85101425	53.5356704	25.95275062	Oncofetal suppressor
CFL2	19.99682538	29.28801152	31.82684864	15.54485304	Oncofetal suppressor
CRAT	9.312399836	16.81582531	20.8267371	8.416256494	Oncofetal suppressor
GSTA1	85.83013023	606.7240953	1195.515429	437.3732789	Oncofetal suppressor
SLC39A8	4.315050852	9.078574153	11.08237989	3.138685321	Oncofetal suppressor
MTTP	10.24740642	54.2474923	77.20983901	10.9179673	Oncofetal suppressor
IGFALS	0.052591394	9.620281107	6.238228912	0.473440255	Oncofetal suppressor
FGG	893.3689504	1505.533921	1672.295395	606.6177518	Oncofetal suppressor
MGMT	2.204958231	8.61972168	7.295752445	1.943719148	Oncofetal suppressor
NDUFAF1	5.342351872	8.716110985	11.98068809	5.637544177	Oncofetal suppressor
GOLT1A	2.029897161	11.12941791	15.931482	6.263364515	Oncofetal suppressor
MSRB1	3.123960784	16.62000066	19.57171319	7.240426953	Oncofetal suppressor
GRHPR	4.151087444	27.46967125	24.76762804	5.437139412	Oncofetal suppressor
HPN	15.97548025	63.38580472	63.21037965	23.01793462	Oncofetal suppressor
C8A	0.498172255	211.8380186	220.9030627	22.10887217	Oncofetal suppressor
ACYP2	1.739530004	4.082943501	5.551729503	1.943095506	Oncofetal suppressor
RUNDC3B	0.064496726	4.454876692	5.27216884	1.339636895	Oncofetal suppressor
TNFAIP2	0.112574005	6.248205556	10.88835885	2.34025406	Oncofetal suppressor
ACOT12	0.180794275	23.52321202	40.72037853	0.961273558	Oncofetal suppressor

CCL19	0.05331337	7.791278388	16.16559298	0.633520337	Oncofetal suppressor
ACSL1	14.43826142	146.007835	177.2627435	26.81619614	Oncofetal suppressor
FCN3	0.318600894	21.28489527	40.45862151	0.113577596	Oncofetal suppressor
HLA-E	52.2250242	101.3711727	141.5594115	51.02203403	Oncofetal suppressor
CHI3L1	2.702477857	5.854726359	30.99675336	2.429187495	Oncofetal suppressor
SLC9B2	5.743933114	8.576857747	10.47098756	1.685575428	Oncofetal suppressor
MNDA	0.053649977	9.310558754	9.530074079	3.586051251	Oncofetal suppressor
SERPINF2	31.93284651	347.1833184	323.7977958	187.8700659	Oncofetal suppressor
CARD16	0.564433192	3.818161603	5.909486856	2.094322428	Oncofetal suppressor
GLTPD1	1.404586193	4.664446594	5.102905393	3.002563283	Oncofetal suppressor
BCHE	0.035842084	48.9392608	56.82525212	11.25999149	Oncofetal suppressor
HDHD3	6.356346642	16.0817062	13.30902333	8.737327533	Oncofetal suppressor
CYFIP2	2.199530346	8.799623152	8.792555437	0.973001942	Oncofetal suppressor
CYP27A1	6.225939948	77.94711162	78.14788851	20.15455956	Oncofetal suppressor
CCDC3	1.361685159	6.818993851	8.056375874	3.020991269	Oncofetal suppressor
PQLC3	3.192568613	5.656932896	6.369278018	3.688151612	Oncofetal suppressor
SHMT1	1.936426201	65.18935698	70.32609247	15.33959718	Oncofetal suppressor
DUSP6	3.302409643	14.49594291	30.67102122	9.971425194	Oncofetal suppressor
DPT	0.177820685	22.57426628	11.89426293	0.776728309	Oncofetal suppressor
CDO1	1.173283813	92.74992435	101.4874017	13.40034035	Oncofetal suppressor
SERPINA4	1.311125802	139.3739545	126.404017	34.26079361	Oncofetal suppressor
MSRB2	4.380659812	12.99495628	13.81262145	7.458262148	Oncofetal suppressor
SASH3	0.066213986	4.139514378	4.412592699	1.444333319	Oncofetal suppressor
XPNPEP2	5.176088427	0.017649631	15.83244768	0.044749573	Oncofetal suppressor
UGT3A1	2.856019515	8.954821436	11.16051865	2.760919435	Oncofetal suppressor
MCCC2	3.906286671	24.29724867	25.73121927	15.31823712	Oncofetal suppressor
SLAMF7	0.002999167	3.564555796	5.950928053	1.146926499	Oncofetal suppressor
STEAP1	3.016552472	6.829954501	6.221707529	1.197911643	Oncofetal suppressor
ALDOB	39.9202942	4436.466172	3729.789585	1907.115059	Oncofetal suppressor
HLA-B	8.884534981	43.50684736	203.3536791	31.37075802	Oncofetal suppressor
CTH	3.60362159	23.57670902	58.4187643	3.568472569	Oncofetal suppressor
COLEC11	3.331559747	19.00549186	13.45339121	2.266338384	Oncofetal suppressor
EPB41L4B	1.190050313	13.59672707	8.92204269	5.266020241	Oncofetal suppressor
TMEM150C	0.864927119	5.131800644	5.262893071	2.057565879	Oncofetal suppressor
ACSS2	6.626279461	11.31315251	27.12237689	7.895275098	Oncofetal suppressor
SIGIRR	1.678623901	5.489425546	5.656973074	0.787213596	Oncofetal suppressor
C11orf1	2.277932353	7.041116894	4.346406224	2.301139613	Oncofetal suppressor
PLA2G16	4.510785117	11.80811843	14.77539634	2.407596222	Oncofetal suppressor
GPR183	0.779755072	2.531510747	5.663478999	1.066092766	Oncofetal suppressor
HYAL1	0.348290184	25.70617233	27.07502063	5.722287338	Oncofetal suppressor
LHPP	2.109957936	6.201691079	6.599222498	2.686736167	Oncofetal suppressor
OSGIN1	0.351190266	12.56303429	7.706614131	3.485551992	Oncofetal suppressor
IRF8	1.309950119	4.067009236	6.301442707	1.805119824	Oncofetal suppressor
HRSP12	5.128494388	199.2238194	254.3735608	79.48974865	Oncofetal suppressor

ECT2	3.776591515	0.243395222	0.86665232	7.31585125	Oncofetal driver
SPOPL	8.206335423	3.005276212	3.731805227	11.88617289	Oncofetal driver
SLC39A10	13.07952612	0.806740622	1.09903144	3.542054526	Oncofetal driver
ZNF841	12.03761142	2.650935664	3.126133681	17.39876325	Oncofetal driver
C5orf42	3.297391758	0.830624546	1.0190406	2.524015925	Oncofetal driver
FAR1	9.5650198	1.649824381	2.060369066	6.690660711	Oncofetal driver
BAX	5.262794143	1.552103196	2.998204796	4.730965669	Oncofetal driver
CBLL1	8.207704256	3.476900045	3.824763296	8.462340794	Oncofetal driver
FBN1	41.87166041	1.73762997	1.354226097	3.200777509	Oncofetal driver
TUBB4A	9.948400704	0.144120635	0.548616419	2.466511891	Oncofetal driver
SCGN	15.51629066	1.152653832	0.800044952	6.061448712	Oncofetal driver
SLC30A6	8.676560391	3.63127417	4.262178716	8.086821831	Oncofetal driver
FAM219A	15.79431834	1.508136515	1.99984443	4.241861946	Oncofetal driver
DZIP1	4.066307455	1.128153664	0.772323475	4.929903354	Oncofetal driver
COL4A2	237.5336716	3.32436818	6.127231611	31.46374535	Oncofetal driver
TMED9	102.372813	16.83586693	26.00792579	48.28919074	Oncofetal driver
IGF2BP2	63.4971352	1.369849896	1.308027032	7.225142233	Oncofetal driver
C11orf80	8.4564485	0.59692011	0.977646486	2.152994067	Oncofetal driver
ZNF230	2.055183949	0.887037745	0.943241123	4.407811269	Oncofetal driver
GALNTL4	2.718232199	0.965125469	1.303317417	2.301018843	Oncofetal driver
ZNF223	5.210951219	1.424497253	1.630514149	6.682775901	Oncofetal driver
GPC3	338.4757085	1.503896967	1.729451655	311.0846369	Oncofetal driver
NUP155	3.767222128	1.949175786	1.790893073	7.711098285	Oncofetal driver
NIPAL3	6.936384593	1.042261613	0.894551893	2.593419526	Oncofetal driver
PJA1	14.31777857	2.106168577	3.221387796	6.936577028	Oncofetal driver
UGT2B11	6.544124984	1.249553622	2.382943008	3.719569199	Oncofetal driver
CNOT6	6.966979158	1.735216495	2.463802773	6.604400979	Oncofetal driver
REEP1	11.04326455	1.152524823	0.323221419	2.601719354	Oncofetal driver
NREP	8.791852566	3.228614022	4.196954247	14.84063965	Oncofetal driver
BCAM	72.86211087	6.533692727	7.296155234	55.77960337	Oncofetal driver
STXBP5	2.658773175	0.967467936	1.414198715	2.702712116	Oncofetal driver
TOR1AIP2	15.66295779	5.272194314	6.02081519	12.24851202	Oncofetal driver
JARID2	6.427497626	1.713292342	1.721335546	5.009332004	Oncofetal driver
ZBTB9	3.563888833	1.432895271	2.109882908	6.096223111	Oncofetal driver
KLHL11	2.850867	0.604862149	0.761089064	2.234661844	Oncofetal driver
MYSM1	13.73246347	3.817372748	4.718907642	9.60278371	Oncofetal driver
ZNF577	4.061621646	2.152124916	1.255253963	8.007933514	Oncofetal driver
TLCD1	4.160354139	0.753912521	1.486450161	5.051820585	Oncofetal driver
EIF1AD	4.756283283	2.147628134	2.569109385	4.996833883	Oncofetal driver
ZNF567	4.303244573	1.600853496	2.092320876	7.83901427	Oncofetal driver
XRCC1	10.58932365	3.977184448	5.397296599	17.62732938	Oncofetal driver
LIMK2	7.022692809	0.670800386	1.263362179	6.306280977	Oncofetal driver
RPS5	245.0762287	134.7334641	101.608574	242.3128899	Oncofetal driver
ZNF8	2.592935534	0.680162991	1.091313203	2.996803976	Oncofetal driver

HELZ	9.998741039	4.720603511	4.700221992	16.99410488	Oncofetal driver
KITLG	5.02563659	0.656221065	0.695346094	2.365762975	Oncofetal driver
TPGS2	11.28603223	3.491730867	4.945129274	13.57230263	Oncofetal driver
ENTPD7	6.8455562	0.979730659	3.15506197	7.568392352	Oncofetal driver
STX6	4.912561891	1.494790698	2.159592613	6.518525888	Oncofetal driver
DAB2IP	4.783917462	1.770630761	1.991859459	5.764008972	Oncofetal driver
RASAL2	6.030117433	1.813798985	2.066909161	6.272437594	Oncofetal driver
UBE2K	34.95874029	14.50612318	16.56487354	36.6400459	Oncofetal driver
ZUFSP	6.482973991	1.721104795	3.319860228	7.505999519	Oncofetal driver
CA11	21.56011373	1.208318129	1.330649478	10.86930037	Oncofetal driver
SMNDC1	13.80816273	6.170080616	7.451979697	15.54784806	Oncofetal driver
MAB21L2	10.72914704	1.079049217	0.739142843	6.630764324	Oncofetal driver
ASB1	2.851078134	0.90034553	1.12746056	2.48508087	Oncofetal driver
VPS72	7.62898711	3.032255494	3.542052419	19.23256462	Oncofetal driver
FAM208A	10.11436675	4.097706217	5.672055939	11.97998541	Oncofetal driver
SSR2	17.14030844	6.835881599	6.982658662	24.69939589	Oncofetal driver
CHMP4C	10.58181817	2.604250608	3.226076227	6.714834088	Oncofetal driver
COL1A2	100.3150453	5.56559811	6.38475476	17.49491194	Oncofetal driver
BRPF3	13.97716562	4.598647087	4.22156489	15.41689728	Oncofetal driver
RAVER2	5.605158642	0.671674576	0.778769732	3.704556899	Oncofetal driver
EFNB2	22.81591628	3.222734445	3.23670686	9.559724363	Oncofetal driver
CKAP2	7.246351685	1.691631265	3.643893755	16.47473062	Oncofetal driver
ZNF35	2.146668933	0.989740208	1.014351649	2.2937727	Oncofetal driver
GPR160	6.374427746	1.039261562	1.272444757	3.79437832	Oncofetal driver
SYNE2	12.31896524	3.35898426	3.963941451	18.59091474	Oncofetal driver
ZNF836	3.020147203	1.186173796	1.273558906	5.404051331	Oncofetal driver
BEX5	2.896726517	1.007285285	0.980149145	3.262551175	Oncofetal driver
ACSL4	13.80592876	2.188714105	5.188047528	209.2536653	Oncofetal driver
C12orf32	3.034584599	1.221108166	1.473255905	7.180745417	Oncofetal driver
MGAT5	8.375363798	0.984192895	1.147138364	3.226580885	Oncofetal driver
ARPP19	28.25712641	7.421418346	9.394766215	17.04067876	Oncofetal driver
FKBP10	43.89562551	1.523856869	1.522180012	3.290869219	Oncofetal driver
CAPG	10.37816573	0.848106453	1.472339006	7.951193267	Oncofetal driver
SRCAP	5.644165056	2.596117671	3.041148958	10.29036546	Oncofetal driver
GOLT1B	28.35786048	3.398892502	4.891772876	19.78189146	Oncofetal driver
CCDC122	3.755550475	0.804630881	1.520763363	3.770529065	Oncofetal driver
GPX7	4.785087733	1.36381928	1.814200102	9.407731024	Oncofetal driver
SUV39H2	2.602828991	0.947254603	1.602004926	5.705099825	Oncofetal driver
ZNF256	3.381590872	1.139288327	1.750060538	6.433385309	Oncofetal driver
APCDD1	5.517547425	0.992715595	0.462244251	2.991246221	Oncofetal driver
LNX2	15.63233298	4.033656125	5.277283903	13.42487187	Oncofetal driver
TAF7	53.32598287	23.33492282	27.99132942	70.50911155	Oncofetal driver
DMKN	3.799149901	0.146902072	0.132455955	4.318945182	Oncofetal driver
TOP2A	9.194001156	0.355076459	3.405073513	56.35581989	Oncofetal driver

HDAC5	15.09644072	4.758365158	3.810348628	10.06802386	Oncofetal driver
TNPO1	19.58352415	4.487922601	5.567389323	10.27101327	Oncofetal driver
NUDCD1	7.701962358	1.20606472	1.403941615	4.239061816	Oncofetal driver
MLLT4	34.28279615	13.74538613	16.6080895	45.17126694	Oncofetal driver
RPL18	65.27793346	28.85414787	26.3768301	57.81615048	Oncofetal driver
MCMBP	9.891513608	4.648963385	4.852818651	10.81849533	Oncofetal driver
FBXO30	5.487293186	1.449291729	1.95766027	7.874125028	Oncofetal driver
UBE2D2	21.37294044	7.660499366	9.439624384	19.38472438	Oncofetal driver
CCDC43	12.10959079	3.611652667	4.764944997	10.66412881	Oncofetal driver
ENC1	22.62369001	3.907235339	4.310349787	10.86447786	Oncofetal driver
PAIP2	25.62497393	6.728863407	9.705306011	17.01334792	Oncofetal driver
SUMO2	76.57864349	27.47584343	30.00311234	126.8766006	Oncofetal driver
PARG	3.965724629	1.557267369	1.855005228	4.302316561	Oncofetal driver
TBL1XR1	16.70795495	6.171162699	7.03680892	15.59953078	Oncofetal driver
ITGB1	216.6194131	84.14811671	114.6169177	267.1162777	Oncofetal driver
SOCS4	4.501425759	1.280556759	2.514876502	6.738168685	Oncofetal driver
RND2	3.440406457	0.703889137	1.03578601	3.867380035	Oncofetal driver
PLAGL2	6.762490211	0.985347477	1.701155472	5.843721613	Oncofetal driver
SMARCC1	17.6842016	6.986744998	8.505191203	16.43061492	Oncofetal driver
TMED3	17.52823835	1.04108812	1.714130259	3.805895419	Oncofetal driver
NR6A1	4.105843968	0.794536637	0.599576153	2.069420602	Oncofetal driver
IVNS1ABP	18.41088363	4.947194361	6.740514099	38.8687487	Oncofetal driver
TMEM181	8.876676038	3.331272276	4.753800807	17.67151501	Oncofetal driver
PTTG1	3.057031051	0.276598689	1.295893708	6.734699402	Oncofetal driver
HIPK1	7.411304703	2.98086907	4.114327754	8.651260347	Oncofetal driver
PYGO2	6.504671697	2.461872262	3.162583574	9.449186672	Oncofetal driver
ANO6	44.42086787	13.00485939	17.42940756	93.84368537	Oncofetal driver
CCNB1	5.441215023	0.547382342	1.410498851	8.843201366	Oncofetal driver
CDKN1A	28.20265933	4.291662368	22.88566427	33.65718937	Oncofetal driver
SOX4	40.84112445	2.943414953	3.248711385	41.62462835	Oncofetal driver
PKNOX1	5.615999561	1.349700879	1.477208588	4.264583205	Oncofetal driver
PDE9A	7.638279153	1.481974619	0.600889567	5.170929567	Oncofetal driver
SLC37A1	5.487108736	0.527785685	0.725808806	3.04904429	Oncofetal driver
MYBL2	2.203539764	0.115163511	0.417254367	9.026666269	Oncofetal driver
MATN2	13.76634998	6.852858268	5.514280512	12.83122216	Oncofetal driver
PRPF39	11.95475564	4.538254917	4.850988973	11.4051527	Oncofetal driver
GGA2	10.80193899	3.44980125	3.431785581	7.630424681	Oncofetal driver
USP37	2.129935112	1.041752508	0.976143831	4.128136056	Oncofetal driver
PPM1D	6.76490318	1.930821433	4.414025246	13.99620117	Oncofetal driver
MAPK1	14.4025861	5.069701742	5.912915152	12.4762725	Oncofetal driver
RGP1	4.484768172	1.583058792	1.418865237	3.04770768	Oncofetal driver
ANXA2	66.90275533	6.941547845	11.0950555	21.10158957	Oncofetal driver
MAPK1IP1L	22.68319985	8.601922009	12.75393695	21.79478201	Oncofetal driver
COL12A1	115.8530973	1.260957367	1.118135925	4.26835458	Oncofetal driver

FOXJ2	9.033643943	2.662208687	2.237122733	6.919267663	Oncofetal driver
FOXJ3	15.11025264	4.52869772	5.307154425	13.47745732	Oncofetal driver
BCL2L11	6.720342648	1.596678245	2.456593451	9.291769442	Oncofetal driver
TEAD4	2.528255147	0.308143493	0.823749336	2.787000248	Oncofetal driver
CALM3	110.2929392	34.02123381	36.58178728	104.0846515	Oncofetal driver
PRRC1	16.99782547	2.725811681	4.267546272	9.293002864	Oncofetal driver
RNF38	8.707100983	3.178197897	4.049251721	8.116415532	Oncofetal driver
UBAP2	9.51865242	2.293952935	2.331333002	5.702301842	Oncofetal driver
SPAG9	18.30572481	7.811538339	8.017138049	18.92559767	Oncofetal driver
ZBTB1	14.72841615	3.957601689	6.085115542	11.26159893	Oncofetal driver
HSPA2	7.932038567	0.652309474	1.127375323	3.64643651	Oncofetal driver
USP39	8.438704843	3.250165082	4.577846294	9.617134733	Oncofetal driver
MED10	16.76810399	3.953955373	5.131493998	15.36481346	Oncofetal driver
ZNF766	11.93753819	4.777705025	4.981238487	19.66269112	Oncofetal driver
NSUN5	3.259923596	1.399287256	1.525920535	3.09996309	Oncofetal driver
CCDC14	12.62766603	4.628289179	5.071938575	16.18033483	Oncofetal driver
UBE2Z	10.58713815	4.255689083	4.698675269	13.68233942	Oncofetal driver
ZNF587	9.211508006	2.763590343	3.193485675	12.37309717	Oncofetal driver
TPM2	31.55894766	6.715465482	4.504709119	12.4977334	Oncofetal driver
PAFAH1B3	2.992339648	0.596106907	0.670277529	6.088754955	Oncofetal driver
NONO	50.53642099	25.25099055	25.07983906	79.27560246	Oncofetal driver
KPNB1	46.3125562	18.82657917	24.65704921	57.12302212	Oncofetal driver
TUBD1	7.506972975	2.891887836	2.593293933	6.996059987	Oncofetal driver
NUFIP2	24.19562797	5.801678578	9.732957585	28.31331095	Oncofetal driver
ANKRD27	6.145919273	1.923636665	2.422092824	8.677578304	Oncofetal driver
H2AFX	5.578018648	1.990230964	2.869058354	7.596891556	Oncofetal driver
ERH	43.44097926	18.71539994	22.7427243	51.39354633	Oncofetal driver
C1orf144	20.91731581	8.675473242	10.01505046	22.02127707	Oncofetal driver
EFNA4	4.252870388	1.2390609	1.237409194	5.544978747	Oncofetal driver
CAPN6	14.59377288	0.155819329	0.15141359	49.948199	Oncofetal driver
SKIL	24.3142192	4.037808499	7.028999412	17.35181652	Oncofetal driver
CBX5	14.90605274	3.706651643	4.771061319	17.99482904	Oncofetal driver
MID1IP1	14.70313624	2.454742224	7.897907753	27.04483253	Oncofetal driver
PHF10	21.37343674	10.16950228	9.890716583	24.04832745	Oncofetal driver
ETNK1	3.850763107	1.532578077	2.15110707	6.502369995	Oncofetal driver
GTPBP4	9.500535919	2.837519744	3.905232679	11.50516531	Oncofetal driver
TCFL5	2.435316243	1.360363944	0.920920972	2.98243661	Oncofetal driver
ARFGAP1	11.73171587	1.894019868	2.33226014	4.877771145	Oncofetal driver
LCOR	6.756367288	2.341595938	3.099525706	8.80790991	Oncofetal driver
MGAT4A	9.268612607	1.199996002	1.558301239	7.525978633	Oncofetal driver
FADS1	10.60218017	0.929900288	5.141459256	12.8324341	Oncofetal driver
MEP1A	47.4548165	0	0.053373216	2.305562365	Oncofetal driver
ARL5B	36.58702594	4.721447884	10.37383283	21.39223607	Oncofetal driver
HSF2	8.595032949	2.259178801	2.808275016	5.769339098	Oncofetal driver

ENO2	8.379690515	0.918830885	0.803566126	3.573821725	Oncofetal driver
ATN1	37.67179757	6.590059707	7.385732927	30.60157046	Oncofetal driver
TNIK	5.95851539	1.657483978	1.922238974	5.416201805	Oncofetal driver
SALL2	3.340726043	0.241798295	0.287707995	4.191362605	Oncofetal driver
RUFY2	3.579200983	1.62609118	1.839827411	4.319488771	Oncofetal driver
TBP	9.218999169	3.810945446	4.215029827	12.36819319	Oncofetal driver
ATP1A1	195.9138868	14.97621759	18.16507886	70.04254178	Oncofetal driver
CD248	7.23336124	1.191361567	0.791991333	6.612720309	Oncofetal driver
CCNA2	3.761823381	0.250304618	1.565903739	6.399652898	Oncofetal driver
JAG1	4.792845632	1.935982398	2.349351075	9.373407761	Oncofetal driver
DR1	12.18246758	4.532822114	5.063412539	14.4055328	Oncofetal driver
GTPBP2	4.585769813	2.17688974	2.307304684	7.852503041	Oncofetal driver
TARSL2	11.51282678	2.183102219	2.984236902	5.998824725	Oncofetal driver
FADS2	5.822070426	0.270789877	2.855839765	6.244079257	Oncofetal driver
AGFG1	17.02380188	4.402858993	5.766101293	14.41826553	Oncofetal driver
DONSON	12.42734645	3.200080571	5.712412768	20.69484346	Oncofetal driver
TCEAL4	225.9017622	44.42983482	51.19820922	105.9750439	Oncofetal driver
PYCR1	6.908865896	0.107023055	0.233993281	2.22507405	Oncofetal driver
ZFR	27.00439127	10.82919524	11.78345752	27.9586002	Oncofetal driver
CEP192	4.318958978	1.549209521	1.730626621	4.557793949	Oncofetal driver
ARL6IP6	4.535158033	1.083086406	0.879164586	3.195420572	Oncofetal driver
ZNF451	5.125220474	2.272856647	2.481559636	5.092118236	Oncofetal driver
CCNB2	3.359355754	0.248223116	1.205240322	11.29160456	Oncofetal driver
EDC3	4.478249914	1.452644497	1.746819685	3.981247519	Oncofetal driver
SALL4	4.046223503	0.289017591	0.156787928	2.661172581	Oncofetal driver
RAD1	4.438702612	1.942511962	2.482055769	6.54072938	Oncofetal driver
C16orf53	8.136585196	2.058099644	1.774971271	5.696613953	Oncofetal driver
POLR2M	3.670085056	1.561688239	1.337716891	3.994493571	Oncofetal driver
ZNF579	3.538102637	0.960163809	0.766075669	4.059691169	Oncofetal driver
PARD6B	13.10986588	1.350673525	1.747357313	4.881169027	Oncofetal driver
CASK	12.25050668	1.821179538	2.650261718	6.078883112	Oncofetal driver
ZNF764	2.656951324	1.031876895	1.072256414	3.378139939	Oncofetal driver
MPZL1	40.9263864	2.787637941	3.529167031	12.27463882	Oncofetal driver
YEATS2	11.53338416	1.245632702	1.306943702	3.86565173	Oncofetal driver
GDI1	19.74548855	5.409661666	5.671801505	14.21176186	Oncofetal driver
BMP8B	5.313310137	0.108100985	0.296375457	2.636422435	Oncofetal driver
42254	46.28475627	11.69163385	12.87937082	30.46077555	Oncofetal driver
PPDPF	16.6859151	6.230072239	7.513182656	20.32955916	Oncofetal driver
ACTN4	80.23123743	22.59723868	24.0328801	141.9614124	Oncofetal driver
PVR	30.55856632	5.49459626	7.694364467	19.2208254	Oncofetal driver
SYNM	14.86964978	1.862113542	0.937820238	14.15524351	Oncofetal driver
TMEM39A	12.55499364	3.212433553	3.751792777	9.121092392	Oncofetal driver
C20orf194	3.103097941	1.056973405	0.953401944	3.594505403	Oncofetal driver
PRCC	11.67606941	4.919630361	5.798663203	15.69940569	Oncofetal driver

TMEM167A	33.23187003	7.686052134	12.33223054	23.56920251	Oncofetal driver
DCTN5	6.404910526	2.678177783	2.150472964	4.978921271	Oncofetal driver
UBE2I	3.217587724	1.177070154	1.221501362	2.623175966	Oncofetal driver
MAN2A1	25.26607448	7.83564925	8.189716752	16.22953194	Oncofetal driver
ANKRD10	25.01516882	8.743551637	10.43525172	23.74423634	Oncofetal driver
GPC6	19.9860526	4.119816602	4.448598531	14.18036199	Oncofetal driver
CBFA2T2	3.841522273	1.876879053	1.609738327	6.354940931	Oncofetal driver
CAND1	13.37333959	4.83603757	4.797110742	11.38648048	Oncofetal driver
TAOK2	3.922323106	1.636750909	2.238731809	4.105420078	Oncofetal driver
SEMA4B	5.337344314	1.371574253	1.96219079	4.407299968	Oncofetal driver
TMEM101	9.007197284	3.547553723	2.950769208	7.765531375	Oncofetal driver
MORF4L1	90.8960481	23.33715083	28.0741979	74.80124915	Oncofetal driver
RPL38	163.480136	60.71967687	46.65631	204.5694761	Oncofetal driver
WEE1	7.209191776	1.703361162	3.984856365	7.175037003	Oncofetal driver
TMEM91	2.085037784	0.75400146	0.633678489	3.133652613	Oncofetal driver
RAB11FIP3	23.78667513	3.500868638	3.243734575	7.339014961	Oncofetal driver
FAM50A	28.01975833	9.770208466	11.8081534	42.64894042	Oncofetal driver
RERE	30.22718818	5.536702786	5.578825731	15.76719281	Oncofetal driver
GOLM1	63.25102949	5.407750207	18.79817121	38.4880116	Oncofetal driver
ZNF192	5.516945565	2.355976629	2.543154334	8.135820517	Oncofetal driver
UNC5B	4.878574669	1.041451186	1.023808439	3.905203189	Oncofetal driver
ZNF609	8.652177403	4.133423866	4.232809547	11.93435119	Oncofetal driver
SLC41A1	3.679570184	0.978221361	0.962140998	4.878315874	Oncofetal driver
OSBPL1A	6.162206751	2.618182555	3.182063608	6.311909963	Oncofetal driver
GREB1L	9.508483017	0.351576191	0.447935744	2.89957004	Oncofetal driver
UTRN	24.58234369	6.64510002	7.00726535	35.20430172	Oncofetal driver
APOBEC3F	2.768447184	0.781222084	1.041493847	2.353424484	Oncofetal driver
ATP1B1	101.0230544	24.40641423	39.27994067	73.83460952	Oncofetal driver
FTH1	73.08803374	13.825544	38.65536734	75.41295017	Oncofetal driver
GTF3C4	3.595955238	1.165842386	1.534080077	3.258608177	Oncofetal driver
AMOTL1	20.08960397	2.823471886	2.807159886	9.346796689	Oncofetal driver
MAX	15.72198031	6.274981546	6.520032256	13.00060955	Oncofetal driver
PTPDC1	2.517946195	0.450974533	0.701225455	2.286837402	Oncofetal driver
POGK	9.587776461	3.16263448	3.719158206	14.34729236	Oncofetal driver
UBAP2L	24.53741618	7.503190526	9.105895259	31.19587378	Oncofetal driver
PLXNA2	2.273472261	0.927883129	0.807842599	2.404546747	Oncofetal driver
FBRS	5.692469635	2.620287553	2.736094959	6.116312568	Oncofetal driver
TULP3	10.10700571	3.743861044	3.465882544	18.7190497	Oncofetal driver
WDR20	3.137362301	1.197034174	1.629809551	3.099127762	Oncofetal driver
TRIO	5.827833708	1.660241922	2.033080328	4.243538069	Oncofetal driver
GPX8	41.00681624	1.229724782	0.783149829	2.09980545	Oncofetal driver
MACF1	15.49065208	5.967855979	6.1293868	13.63155289	Oncofetal driver
IGF2	5242.757018	116.518041	128.4453174	4214.156046	Oncofetal driver
IER3	14.55152252	2.429644335	6.36032787	8.860811446	Oncofetal driver

TNNT2	4.020506109	0.067873871	0.01324984	2.028204787	Oncofetal driver
COMMD2	8.486061239	3.298126924	4.047064093	9.505061289	Oncofetal driver
BCL9	6.37068193	1.356712261	1.322358827	11.34074067	Oncofetal driver
ZNF107	2.748574185	1.121273819	1.380274829	3.402633021	Oncofetal driver
CENPJ	7.640534926	1.169539809	2.058122105	7.859735986	Oncofetal driver
BAG5	9.865593023	3.738533979	5.267438187	10.65041533	Oncofetal driver
ZBTB6	4.368437258	1.80536894	2.208567022	6.909700246	Oncofetal driver
RAB1A	113.6178716	25.49325151	41.84565987	85.38381014	Oncofetal driver
HMGN4	14.59014512	6.393205334	7.083246141	17.81757708	Oncofetal driver
ASXL1	10.14974009	4.088946674	5.081252547	10.89870929	Oncofetal driver
KLHDC5	4.197522046	1.877579223	2.040317561	5.47774226	Oncofetal driver
EPB41L1	7.468952761	2.551961269	1.733281423	6.178048935	Oncofetal driver
MAST2	4.584571378	1.751507772	1.521641753	3.514021215	Oncofetal driver
B3GALTL	3.749917166	0.879389174	1.394471694	3.552720143	Oncofetal driver
CASC3	15.24145349	6.80015213	7.538396761	17.73328316	Oncofetal driver
PDCD4	108.6688705	13.10846694	16.08222666	30.25406626	Oncofetal driver
CADM4	8.388966509	1.88311393	1.737916276	7.074381008	Oncofetal driver
TLK2	6.14804263	2.74181995	3.301898789	9.381041054	Oncofetal driver
TRIP4	8.650472598	4.000019957	4.240463672	13.02519466	Oncofetal driver
BRWD3	8.401941775	1.860575657	1.971392737	7.025831004	Oncofetal driver
TAF11	15.12780359	7.167835226	7.314782305	16.0305432	Oncofetal driver
DYRK2	4.258510687	1.518207817	1.658336278	8.837734271	Oncofetal driver
TROVE2	11.99984258	3.68524753	6.043549121	10.30734779	Oncofetal driver
SCNM1	5.301886561	2.556241991	2.623944479	10.14053796	Oncofetal driver
CKAP2L	2.333738447	0.082771275	0.405418967	5.854320151	Oncofetal driver
ANTXR1	9.057547425	3.074219924	3.269561024	6.798545374	Oncofetal driver
RCN2	14.61971773	3.312366272	4.749042103	20.73667995	Oncofetal driver
FAM189B	5.130158936	1.11067319	0.837761187	2.283550273	Oncofetal driver
ZNF621	4.098514666	1.618459761	1.830488825	4.401490752	Oncofetal driver
PTPRA	42.96485319	9.937132173	8.767855693	23.94781978	Oncofetal driver
CYTH2	4.902340663	1.154183263	1.811958646	4.557783294	Oncofetal driver
KIAA1522	13.21555759	1.346283644	1.947232069	5.690285169	Oncofetal driver
C15orf39	5.193503737	0.673147762	0.710427621	2.844970265	Oncofetal driver
PDGFB	3.895363109	0.542018702	0.851601514	2.040561486	Oncofetal driver
DBF4	2.802005528	0.916563843	1.865028785	6.185654467	Oncofetal driver
KIF5B	58.01448937	19.47705964	29.86251846	79.73373362	Oncofetal driver
CTF1	2.381576143	0.618727035	0.521784125	2.151421773	Oncofetal driver
GOLPH3L	11.74872426	5.457695007	5.674488745	27.76912699	Oncofetal driver
STK39	23.65302186	0.518780839	0.663288191	3.608836523	Oncofetal driver
KRAS	10.94328098	3.30215517	3.931812262	8.187541684	Oncofetal driver
NARF	3.46848187	1.547339288	1.660905928	3.804779349	Oncofetal driver
FOXK2	2.816638538	0.867671929	1.079529511	3.372678249	Oncofetal driver
PTPLB	45.09135404	13.64539748	16.72351113	67.57595285	Oncofetal driver
OSBPL3	3.143487909	0.683832397	1.241035985	3.9473543	Oncofetal driver

ZBTB41	2.583579921	0.894093131	1.132277655	3.232567831	Oncofetal driver
FLNB	46.044029	7.921635475	7.183562122	24.46220203	Oncofetal driver
AC005035.1	26.89322039	9.526728375	8.909170891	19.26717722	Oncofetal driver
SERAC1	3.3218859	0.98172761	1.01376652	4.506524733	Oncofetal driver
BARD1	3.58984897	0.292122447	0.68112922	4.421807021	Oncofetal driver
C12orf35	51.47925809	19.11600573	19.46865961	47.74006211	Oncofetal driver
PKDCC	12.1511832	1.064973466	1.327259288	4.155724667	Oncofetal driver
KIF11	2.128924224	0.70785022	0.548091726	9.225339079	Oncofetal driver
TUBB6	21.43541636	1.408549687	2.837656946	5.06996016	Oncofetal driver
ERI1	4.320053417	0.864554469	1.203067231	2.488813783	Oncofetal driver
SYF2	41.72769412	18.68486614	20.31352909	45.52638298	Oncofetal driver
SLC29A1	21.57500287	10.46656171	10.37503483	73.39425288	Oncofetal driver
ILF2	56.11561261	17.68435691	23.3943625	85.80119779	Oncofetal driver
RIT1	8.914630278	2.018433665	3.180618994	10.34383003	Oncofetal driver
B4GALT5	25.7126588	5.027889625	7.853424461	14.04465629	Oncofetal driver
CD2AP	23.60948339	6.884079257	9.128480118	44.08671445	Oncofetal driver
LMNB1	4.463436369	1.501149236	2.860987894	26.76972386	Oncofetal driver
UNC45A	3.274923906	1.359456585	1.307808224	2.836240535	Oncofetal driver
BCL9L	9.028113193	1.326083359	1.288116442	4.39678287	Oncofetal driver
PTPN12	27.1883763	9.115181568	14.02664743	32.29953944	Oncofetal driver
HIP1	10.23466635	3.52578626	3.892071695	8.639521583	Oncofetal driver
NFKBIL1	5.107235005	1.94453491	1.7461502	5.496223539	Oncofetal driver
RASEF	3.110938151	0.789707492	0.94310207	12.82789682	Oncofetal driver
GJA1	135.4354795	2.881552581	3.805517301	11.21317911	Oncofetal driver
PACSIN2	23.60779394	5.231576709	7.229650446	12.71416049	Oncofetal driver
PLXNA3	5.264851803	0.679399358	0.621332698	2.474603542	Oncofetal driver
DUSP9	18.34874929	0.014254923	0.086821539	3.640065638	Oncofetal driver
MEGF8	6.20606866	2.03754952	2.460396743	9.070534453	Oncofetal driver
DSC2	25.26970242	3.330299154	4.531627543	8.818323319	Oncofetal driver
IGBP1	28.92150088	8.039650045	9.191271892	19.61098361	Oncofetal driver
BTG3	20.29907703	3.625343326	4.016184028	17.10613552	Oncofetal driver
REXO4	15.54016005	5.474622507	4.788341379	11.44996767	Oncofetal driver
BACH1	15.04742329	5.093038155	4.743301519	17.16828651	Oncofetal driver
FBXO5	2.300758477	0.570931464	0.671241831	4.266601966	Oncofetal driver
MKL2	4.060489401	1.042106815	1.255801448	3.579317991	Oncofetal driver
CAP2	3.910477758	1.098350215	2.307690839	8.440529833	Oncofetal driver
MAD2L1	3.991087365	0.542995358	1.406676762	4.234318427	Oncofetal driver
PRC1	2.460118074	0.089478482	0.747601907	12.51011054	Oncofetal driver
MORF4L2	105.4281029	23.67575283	33.83203943	71.72995143	Oncofetal driver
COPA	55.42763138	19.99319118	23.70005864	61.91123515	Oncofetal driver
MED28	15.94034301	4.487930952	4.226667727	9.72852739	Oncofetal driver
CCNK	7.399629782	2.29209011	2.896509868	5.618194552	Oncofetal driver
MARK2	4.250044891	1.754934864	2.129903223	3.886529463	Oncofetal driver
HMGN2P42	3.085915155	1.325950786	1.534783205	3.135860221	Oncofetal driver

CWF19L1	3.855211347	1.589498899	1.847234952	3.645015335	Oncofetal driver
C1QL1	14.47095454	0	0.295847734	3.744047598	Oncofetal driver
VGLL4	3.791163221	1.029869211	0.898754589	2.224784678	Oncofetal driver
SPICE1	3.162957814	0.980164603	1.564740565	3.113479287	Oncofetal driver
BICC1	4.386147015	1.928370638	2.068413259	13.63202689	Oncofetal driver
YWHAG	40.16718952	13.81179818	21.25734748	46.95419137	Oncofetal driver
SCTR	2.633073789	0.454427272	0.093145463	3.441087846	Oncofetal driver
DNM1L	11.31331373	4.270732357	5.666929396	12.76957467	Oncofetal driver
BCORL1	3.850950835	0.88586607	1.129288064	7.628022492	Oncofetal driver
VEZF1	13.18115798	5.920347152	6.529692888	23.84183618	Oncofetal driver
CCNE1	9.448329464	0.380313061	0.144241467	7.300831812	Oncofetal driver
HNRNPUL1	49.7882896	21.935323	21.49949371	127.6149633	Oncofetal driver
CCDC9	8.786407977	2.271383904	3.764084326	15.66019494	Oncofetal driver
CORO2A	12.54014009	1.797275955	1.086724228	2.896159169	Oncofetal driver
PODXL	19.1004899	1.446887064	2.053253191	7.519981216	Oncofetal driver
DLGAP5	2.040911971	0.056152811	0.599826449	5.387765593	Oncofetal driver
B4GALT4	12.77230895	2.201778041	3.154076915	6.645108024	Oncofetal driver
ABCC4	4.415122398	0.474475947	1.039775964	10.32256005	Oncofetal driver
RIOK1	16.04340439	6.164012182	8.48191412	19.34033132	Oncofetal driver
SSR1	29.72354155	10.51683403	13.18802995	43.11027892	Oncofetal driver
FARP1	17.60134262	8.695032173	8.351615475	50.91939774	Oncofetal driver
VN1R1	2.544992673	0.940955175	0.734745523	3.749012199	Oncofetal driver
WDR45L	23.0707812	7.540043127	8.737505805	18.21820556	Oncofetal driver
GPC4	16.14820533	0.91208954	0.675323687	7.548227343	Oncofetal driver
FAM60A	15.00929031	1.078281482	1.661575614	4.433695504	Oncofetal driver
SYNGAP1	4.395624355	0.593532526	0.8138815	2.299770654	Oncofetal driver
ZNF608	6.914058359	1.731806243	1.472175203	7.527240295	Oncofetal driver
NEDD1	5.640242837	2.030049994	2.576093881	4.738776916	Oncofetal driver
ATF1	12.42734645	5.431953227	6.97780186	16.80113892	Oncofetal driver
PGS1	6.141762142	1.076966733	1.725026145	4.350930757	Oncofetal driver
ATXN7L3	11.27430945	3.596199765	4.186776437	7.99497628	Oncofetal driver
PHIP	16.38360332	8.233612051	7.944627731	23.3300147	Oncofetal driver
ACTG1	316.9103261	56.76428025	75.32343472	145.8872165	Oncofetal driver
ZNF518B	3.39601031	0.662771549	0.868735347	3.250037756	Oncofetal driver
LDOC1L	2.31936928	0.914333307	1.183297302	2.675201946	Oncofetal driver
C16orf87	3.958970745	1.75950906	2.182708515	4.022230856	Oncofetal driver
KRT19	107.3349567	0.738037123	1.184289959	2.135228276	Oncofetal driver
MFSD6	3.42513869	1.235952137	1.132519279	15.78960224	Oncofetal driver
ARID5B	22.76892291	4.562658679	5.015035986	11.8095241	Oncofetal driver
TUFT1	13.11723897	0.594618707	0.955834969	7.992984453	Oncofetal driver
RPL13A	424.2400822	143.7914118	130.4894791	463.0033802	Oncofetal driver
ZNF28	6.275804152	1.409694356	1.93755546	4.708302412	Oncofetal driver
ATP11A	10.78935506	1.503509332	2.557903512	8.694845444	Oncofetal driver
NUP188	3.525451819	1.569758427	1.706047854	3.506060306	Oncofetal driver

MAPK8IP3	6.228105677	1.521569194	1.767169783	3.554730292	Oncofetal driver
F2RL1	44.90687134	0.348060104	1.428491351	2.322197241	Oncofetal driver
CTNNAL1	34.3769855	6.646579769	8.398528777	15.99629187	Oncofetal driver
TES	19.04429546	1.876304191	2.772445496	4.89840535	Oncofetal driver
PLEKHG2	3.672888611	0.598170946	0.966967145	3.569943197	Oncofetal driver
EZR	193.8316703	15.40021978	32.62005825	80.20985344	Oncofetal driver
ADNP2	4.698008869	1.652749789	2.049602123	3.960105593	Oncofetal driver
SIPA1L3	5.435215917	0.997106465	1.234953723	11.71437938	Oncofetal driver
HSPA4	48.68414455	14.15998946	20.38222208	34.95023704	Oncofetal driver
UBE2Q2	39.58387727	3.449672793	3.737793004	18.53927456	Oncofetal driver
CSNK2A1	40.2811185	13.46584836	13.28381005	33.62377904	Oncofetal driver
RAB3B	9.329432897	0.060920816	0.094639388	2.341304343	Oncofetal driver
LAPTM4B	30.12140346	8.926947994	7.236160933	32.34090965	Oncofetal driver
MAML1	2.777132821	1.198105578	1.523338476	2.909350155	Oncofetal driver
TJP1	19.41544574	5.845078293	7.054202352	16.28781853	Oncofetal driver
PRKCI	18.2548592	3.133985375	3.23390424	6.864512465	Oncofetal driver
SGMS2	6.413064181	1.96877565	2.605441306	5.02297105	Oncofetal driver
KPNA2	10.53202221	1.482044356	4.375039876	19.52449518	Oncofetal driver
DHX40	10.32892277	3.944512821	4.038145474	13.1450034	Oncofetal driver
ARHGEF17	5.402250057	1.383686142	0.887959876	2.345611327	Oncofetal driver
UBE2J1	48.66975025	14.44266395	15.65141554	33.86989817	Oncofetal driver
ATXN2L	19.71715676	4.499191337	5.54106733	11.78905082	Oncofetal driver
FLT1	13.15299346	2.369828791	2.732100982	6.749579409	Oncofetal driver
TPR	40.35428775	14.86088497	17.42562612	42.7648546	Oncofetal driver
ZNF852	3.059039127	0.55559784	0.611941287	3.082159931	Oncofetal driver
42256	29.0200858	7.480652863	9.787107815	19.664125	Oncofetal driver
DDOST	34.52779613	12.04032657	15.5773338	29.06407329	Oncofetal driver
TCP1	32.80174018	13.15098602	16.58169045	45.59390445	Oncofetal driver
TPM1	120.3864921	5.652400376	6.144850023	22.13345593	Oncofetal driver
BTAF1	21.83608061	7.406726266	8.868944387	19.5258139	Oncofetal driver
GNL3	43.4761531	12.01465714	15.58439985	31.28963505	Oncofetal driver
SERPINI1	3.95977715	0.93058173	0.871974494	2.950824342	Oncofetal driver
FBL	67.34445	31.50600589	29.78700011	221.594964	Oncofetal driver
KHDRBS1	34.30104637	15.3745598	16.10556862	37.31625476	Oncofetal driver
B3GNT5	2.783684893	0.065770532	0.317623088	3.413941617	Oncofetal driver
CDK2	7.180670139	1.433031558	1.957189236	3.924493873	Oncofetal driver
ZNF404	2.880138233	1.252698752	0.75458884	3.573159812	Oncofetal driver
CHML	10.2029798	1.140401866	1.172167208	9.511971236	Oncofetal driver
MTMR2	9.193406593	1.521039149	1.936063459	3.908914317	Oncofetal driver
CAMSAP2	15.0563482	2.803924219	3.329446855	12.51514993	Oncofetal driver
DUSP12	7.828805087	3.482513196	3.077032519	11.41596652	Oncofetal driver
SUPT5H	41.66379157	11.54195754	13.58993758	74.00000504	Oncofetal driver
MCM8	3.4449957	1.066331851	1.343718357	13.89862372	Oncofetal driver
AURKA	2.315508162	0.450081656	1.541971885	6.434279435	Oncofetal driver

ZNF318	6.984361379	3.122817296	3.529990522	12.98645448	Oncofetal driver
CERS6	30.95104063	2.947294864	2.356488672	7.369645051	Oncofetal driver
MAP7D2	3.745946268	0.023006967	0.194022075	11.09989406	Oncofetal driver
ANKRD52	10.80230417	1.505821661	1.725997206	4.185759369	Oncofetal driver
ZNF613	4.685822968	1.267412951	1.607208346	3.801051123	Oncofetal driver
LGI4	42.99805701	4.01612288	3.70405506	8.441198341	Oncofetal driver
PAG1	4.759542745	1.804607637	2.297215326	8.12319523	Oncofetal driver
DMWD	3.889466337	1.357237761	1.308096097	4.821685214	Oncofetal driver
HN1L	21.69688656	8.239949201	7.146380282	21.09367975	Oncofetal driver
THOC2	32.79321174	11.05139731	12.7904072	43.82064398	Oncofetal driver
MZT1	4.3664786	1.047922828	1.384766801	5.742115703	Oncofetal driver
RBFOX2	27.50877236	7.579318448	6.344768984	22.68536117	Oncofetal driver
MCM6	9.368627163	1.270076221	4.263700792	19.53854591	Oncofetal driver
ITPR3	4.91169545	0.666638062	0.542830114	4.431911051	Oncofetal driver
C22orf23	4.199856934	0.428045382	0.707180508	2.227686615	Oncofetal driver
HIC2	7.698830166	0.561916254	0.526527252	2.417555538	Oncofetal driver
PBX2	25.11472408	8.331543709	9.568391652	22.41134655	Oncofetal driver
MTX3	5.008039358	1.197606175	1.620051433	4.380564374	Oncofetal driver
CRMP1	2.821863926	1.20230468	0.82505752	4.084560878	Oncofetal driver
VIL1	8.256962046	2.52034345	1.480539835	9.761485589	Oncofetal driver
RUSC1	2.2059475	0.65260596	1.319002924	2.918988171	Oncofetal driver
TRIM28	33.23945356	12.67902383	11.86746865	38.46587991	Oncofetal driver
UBE2M	22.32153423	9.558768003	11.37648513	38.27072796	Oncofetal driver
PRRC2B	15.34252783	4.623103444	4.503024779	9.162505081	Oncofetal driver
YWHAZ	57.16143576	12.22236062	21.93316301	38.89891277	Oncofetal driver
PRRC2C	81.56273121	33.42883818	35.48911166	127.4410575	Oncofetal driver
42069	19.55665342	8.152528138	9.892696404	28.41848963	Oncofetal driver
HUNK	2.424003488	0.188682652	0.097705032	3.844233263	Oncofetal driver
STRN4	9.94337805	2.864867581	3.560029738	15.49818768	Oncofetal driver
TEAD2	4.163675398	1.412248726	1.832855578	13.45838554	Oncofetal driver
ZNF629	3.236820511	1.087314801	1.079141623	3.467190135	Oncofetal driver
TLN2	9.800880839	0.414315693	0.289380535	3.203894862	Oncofetal driver
ZNF346	2.489948251	1.072185557	1.140536412	3.413993237	Oncofetal driver
CSNK1D	8.600040391	2.060244287	3.038380684	11.42486147	Oncofetal driver
PEAK1	3.116772773	1.264233611	1.537918977	5.563236053	Oncofetal driver
PPP6R1	21.7831742	7.124250429	11.09057548	23.3286071	Oncofetal driver
PGM3	32.23261153	5.052821174	6.922651052	17.53800214	Oncofetal driver
CBX1	29.21318781	8.704749803	9.762837716	46.35139595	Oncofetal driver
RUSC2	4.209035171	1.746739815	1.928762601	4.008717459	Oncofetal driver
CBX6	5.091652532	1.600115953	1.624287196	6.411504808	Oncofetal driver
BEX4	30.76683246	4.992754449	6.102150092	16.79793832	Oncofetal driver
FBXW11	12.39441682	4.618905489	5.01647934	9.648800681	Oncofetal driver
PDGFRB	10.24566101	3.402694544	3.122778645	6.675427089	Oncofetal driver
MSH6	21.91582915	5.352932356	8.109569319	20.88742037	Oncofetal driver

BTN2A1	7.623371481	2.847548987	3.3468334	7.893476486	Oncofetal driver
PDK1	4.279814028	1.607166155	2.615823342	5.037229161	Oncofetal driver
C10orf47	10.44942044	3.400954852	2.823316337	6.474822176	Oncofetal driver
AKAP7	11.25959195	3.870814912	5.03657187	16.37626609	Oncofetal driver
NAV2	16.9833676	2.747475716	3.178013052	12.92571014	Oncofetal driver
SF3A1	17.81071851	7.905185967	9.380374272	23.82470938	Oncofetal driver
FAM161A	2.363583071	0.666509915	0.973537818	3.139797717	Oncofetal driver
METTL6	2.600121534	1.089199283	0.955916379	2.450432935	Oncofetal driver
WAC	21.57322046	10.90219394	10.28378808	33.41937123	Oncofetal driver
PM20D2	6.097529802	1.683862736	1.446671485	13.7394876	Oncofetal driver
ZNF611	7.43291085	1.336923008	1.416479616	4.861400662	Oncofetal driver
MPP6	9.624137167	3.562860128	2.741149211	7.273228927	Oncofetal driver
TMEM51	9.454181982	2.133616209	2.05956488	5.506256065	Oncofetal driver
SEC23IP	15.66900943	5.350678253	5.994711679	12.68718098	Oncofetal driver
DPY19L3	9.785153957	3.029591677	2.907420703	9.586610198	Oncofetal driver
CCDC117	21.23644286	10.70553599	9.691871719	27.51787972	Oncofetal driver
RCOR1	9.75990602	2.696352753	3.288856511	9.287647203	Oncofetal driver
NES	6.618378104	2.230284979	2.603600769	8.429795396	Oncofetal driver
WASF3	4.336116191	1.479920275	1.932431967	7.2559846	Oncofetal driver
MAPK13	2.677916146	0.714451204	0.993613169	4.347474941	Oncofetal driver
C1orf116	3.16217467	0.27694501	0.35068007	3.277731347	Oncofetal driver
PUS7L	3.721304537	1.368714291	1.801626588	12.49507631	Oncofetal driver
ZBED4	5.534296911	1.323918143	1.501125495	5.188294593	Oncofetal driver
NRAS	34.71128162	7.002593768	11.30603422	19.26675812	Oncofetal driver
PCNP	55.02788769	13.81378516	21.05467537	37.7962221	Oncofetal driver
PCID2	5.851916408	2.574311795	2.78775172	7.694320453	Oncofetal driver
FAM219B	4.782172751	1.589966092	1.52652675	3.685158817	Oncofetal driver
FAM127B	10.93737474	4.297301372	2.77595571	10.82479787	Oncofetal driver
C10orf88	4.351768902	1.531525885	1.581011198	3.955958145	Oncofetal driver
MSI2	5.33136179	0.865999994	1.208653134	4.111058289	Oncofetal driver
JOSD1	14.42419957	4.455246568	6.939924532	11.54944478	Oncofetal driver
GTPBP1	4.198587809	1.001067914	1.493273551	2.496134025	Oncofetal driver
RIMKLB	5.789061323	1.11733521	1.193631952	5.620517048	Oncofetal driver
CBX3	31.69974422	13.5263274	16.76462934	30.3129544	Oncofetal driver
RPS19	241.8303257	77.50278576	90.0026485	234.6314678	Oncofetal driver
GLTSCR2	324.9635878	78.50915966	70.85183905	281.7135563	Oncofetal driver
MSH2	4.437904672	1.415873635	2.399086175	11.90503795	Oncofetal driver
ADAM10	33.10633712	6.246270422	8.754755345	35.31988403	Oncofetal driver
CASP2	3.395224052	1.137396189	1.515646257	3.478972406	Oncofetal driver
LRRC1	25.01405859	0.150012837	0.503075301	3.397398555	Oncofetal driver
SLC22A23	3.788011375	1.506506458	1.452827566	7.176628513	Oncofetal driver
42066	3.516695624	0.210723208	0.212354083	2.488487544	Oncofetal driver
AFP	31679.25128	2.647354334	1.941874476	9.055643854	Oncofetal driver
STXBP1	5.807969476	1.205754835	1.420228252	3.801407016	Oncofetal driver

POFUT2	9.068261433	1.445061597	1.882300644	3.943018162	Oncofetal driver
RAB34	12.87461807	1.076585042	1.362436327	5.035671588	Oncofetal driver
TAF12	13.2173128	6.528928405	6.315887606	13.20275161	Oncofetal driver
PPRC1	3.793651844	1.034154248	1.871109042	3.341616875	Oncofetal driver
R3HDM1	6.440778866	2.641038466	3.398143156	6.657476341	Oncofetal driver
SOGA1	3.96318367	0.574398564	0.506338162	2.672609671	Oncofetal driver
RNMT	12.69061149	3.230966452	4.190949916	9.695025028	Oncofetal driver
JRKL	4.05161424	1.23761805	1.812561221	7.687692834	Oncofetal driver
OCLN	25.33300554	5.381557987	7.545398564	17.33458533	Oncofetal driver
BUB3	19.6021592	8.293067157	10.96342171	19.97873532	Oncofetal driver
ZNF701	3.773261113	0.801730557	0.823605245	2.673465285	Oncofetal driver
COL5A1	149.7752376	1.076870455	1.683475006	4.836602326	Oncofetal driver
CENPE	3.510525064	0.422988112	1.184959986	5.264376904	Oncofetal driver
PHC3	5.589018246	2.147416095	2.165996872	4.676479821	Oncofetal driver
SRI	4.181230299	1.837027876	2.290557462	4.742166209	Oncofetal driver
CLSTN1	14.48627541	4.707943987	4.484329434	12.01452375	Oncofetal driver
FOXO4	11.79722635	2.311809757	3.296899294	5.777069897	Oncofetal driver
PTP4A3	5.983774069	0.604066103	1.012882439	7.761505951	Oncofetal driver
KIAA1586	7.81027008	2.515368628	2.927385621	9.72627169	Oncofetal driver
COMP	2.80172076	0	0.018672017	2.08590751	Oncofetal driver
MLL4	5.342308058	2.572081159	2.574107639	6.942259596	Oncofetal driver
MTMR1	4.821517338	2.100748308	2.212215265	5.063655871	Oncofetal driver
ARMCX3	40.45377988	7.679816868	11.6476561	36.51539812	Oncofetal driver
MARK4	9.143660328	3.04450445	3.661112765	17.05525719	Oncofetal driver
RASSF8	9.795363762	2.174045113	2.113137773	9.581638686	Oncofetal driver
TCEAL8	77.7415381	20.49013311	24.56801434	101.5287977	Oncofetal driver
TPM3	38.82730535	14.79033279	21.1216539	58.85186864	Oncofetal driver
COL1A1	220.0038718	5.060255814	6.437367503	30.59414225	Oncofetal driver
LRRC59	28.84002477	8.218471333	12.24372222	23.60368609	Oncofetal driver
AZIN1	31.02487332	10.73601216	13.28910879	26.16134231	Oncofetal driver
KDELR3	60.1503325	3.319789692	3.690845014	14.77733433	Oncofetal driver
VCAN	24.62584479	0.671981532	1.00693676	4.673797344	Oncofetal driver
TMEM65	8.880342571	1.403367987	1.763275447	9.57929209	Oncofetal driver
SYNJ2	3.335532148	1.140405945	1.410608385	13.23440047	Oncofetal driver
AHNAK	113.148334	23.10653438	20.1225932	65.05669958	Oncofetal driver
GPRC5B	13.9789431	1.249600058	1.31408219	6.534588851	Oncofetal driver
NPEPPS	15.44231448	4.528748817	4.789679555	10.96106148	Oncofetal driver
DOCK7	4.221312692	1.759335786	2.21283349	4.879115116	Oncofetal driver
ZNF471	2.652101317	0.775228726	1.534385924	4.369547584	Oncofetal driver
ZBTB34	5.670836433	0.801081984	0.966765391	3.827355235	Oncofetal driver
SPATS2	7.184276198	1.557118265	1.709738888	6.297604126	Oncofetal driver
MECP2	4.030591096	1.92415581	1.958626576	5.831734255	Oncofetal driver
RALGAPB	8.087575665	2.663615967	3.507659063	9.606411797	Oncofetal driver
HN1	4.460300105	1.363618561	2.778663933	7.111015125	Oncofetal driver

JMJD6	3.294478659	1.349252826	1.838040112	4.467994181	Oncofetal driver
SF3A2	26.04207767	6.480807368	6.739590322	13.85364279	Oncofetal driver
VPS33B	2.137465348	1.104920414	0.968537769	2.716622192	Oncofetal driver
MAPRE1	33.99661261	6.40581333	10.98229707	32.03100385	Oncofetal driver
CMTM4	13.02393665	0.900565318	0.720242513	2.762047064	Oncofetal driver
LHFP	12.79940137	5.875625497	3.067870413	11.04797148	Oncofetal driver
UBR5	8.246748958	3.695292035	3.945795277	13.86564844	Oncofetal driver
KIAA0355	6.531139066	2.092091735	2.959266477	8.888980699	Oncofetal driver
UHRF2	5.223130008	2.034419904	2.330347019	6.42663238	Oncofetal driver
VLDLR	11.92723137	0.91310047	0.537948441	2.477505562	Oncofetal driver
KDM4C	3.543675941	1.406396163	1.856167071	3.732743873	Oncofetal driver
RNF219	3.752345722	0.951082864	1.399651704	4.634731904	Oncofetal driver
TRIP12	20.67779109	9.087128118	10.96378374	20.71209077	Oncofetal driver
SLBP	25.77614582	7.683600306	10.72335342	34.31315966	Oncofetal driver
GLS	12.92433914	2.110041151	3.357428901	7.404376261	Oncofetal driver
SVIL	11.82278872	3.731190851	3.255536494	23.5127808	Oncofetal driver
BTBD10	7.56979679	2.026719626	3.080482096	5.294405834	Oncofetal driver
STK24	20.97300609	6.923491628	8.329221807	27.48157231	Oncofetal driver
C1orf9	36.04171746	2.645943544	3.991948434	16.01560019	Oncofetal driver
PTBP3	27.98172763	6.141774171	8.66965911	21.53870732	Oncofetal driver
GAB2	7.830947403	1.827696452	1.991847258	4.656044026	Oncofetal driver
MAD2L2	4.954383394	2.039155582	1.882382189	4.155411411	Oncofetal driver
SLC26A3	3.678638266	0.194831326	0.09698554	2.467710037	Oncofetal driver
MTF2	7.031221905	2.99527859	3.399911705	7.961023864	Oncofetal driver
PHF16	8.29995913	3.132402632	2.694604071	7.169170958	Oncofetal driver
THOC6	5.278281558	1.572019073	1.670108176	4.763664211	Oncofetal driver
KIAA1958	3.195486092	1.136371269	1.248643982	3.530437376	Oncofetal driver
SUN1	8.620245356	3.518908694	4.966189908	9.486924484	Oncofetal driver
OLA1	13.05663649	4.712354933	7.701951877	13.16811202	Oncofetal driver
FAM117B	2.54930556	0.985604513	0.920314107	2.492721372	Oncofetal driver
KCNC3	2.578168984	1.167831755	1.367853395	12.50806526	Oncofetal driver
SLC6A6	10.95360669	0.822453134	1.312450084	3.198332461	Oncofetal driver
FAM126A	15.33818625	2.388857379	2.740795301	8.392567807	Oncofetal driver
NCKAP1	41.16091123	10.58507738	12.11397058	24.40103227	Oncofetal driver
ARID1B	4.647748201	2.336692654	2.207754147	6.432260494	Oncofetal driver
USH1C	10.07736835	0.197346672	0.216393344	2.025775934	Oncofetal driver
UEVLD	8.887486842	3.291028501	4.003480956	7.505791221	Oncofetal driver
HGS	4.085235447	1.359610308	1.818640267	4.714048174	Oncofetal driver
MED14	9.044804188	3.379982254	3.474833463	8.659338954	Oncofetal driver
IPMK	6.970499233	1.836524694	2.334331931	5.465095401	Oncofetal driver
NGRN	7.772051362	2.640470962	2.564272432	12.7545927	Oncofetal driver
APPL1	12.80095574	3.065625347	3.367105785	7.710176141	Oncofetal driver
LTN1	7.464124015	3.20607118	4.236685396	12.30159507	Oncofetal driver
RUNDC1	4.534185096	1.839655858	2.283273536	4.64680126	Oncofetal driver

CPSF6	11.21553134	4.373131707	5.278458844	545.3721939	Oncofetal driver
PPP1CB	40.13956192	12.16527348	14.75219	30.56722127	Oncofetal driver
METTL9	13.92789235	3.436263079	3.510569199	7.385110749	Oncofetal driver
NACC2	3.944095288	1.658833606	2.126880457	3.999043223	Oncofetal driver
LOX	143.8720657	0.621352212	0.405789686	2.369239457	Oncofetal driver
CRKL	14.04154012	5.943423349	6.55466688	18.93348126	Oncofetal driver
UBXN7	11.28891596	2.906955077	2.650397789	5.93701711	Oncofetal driver
CKB	491.6719943	1.766726483	1.734979462	6.20357511	Oncofetal driver
UBA2	35.26357039	12.56681065	12.97405345	64.99838295	Oncofetal driver
DEDD2	5.971097395	3.393680082	2.546004208	6.296243562	Oncofetal driver
LARP4B	15.72962334	3.331668395	4.322762943	14.2324165	Oncofetal driver
C20orf20	7.537096158	2.164848745	4.092397829	10.84548023	Oncofetal driver
HNRNPC	94.10692338	38.31686957	47.49882011	103.4195358	Oncofetal driver
AXIN1	6.034500927	1.201865903	1.141635758	3.400895816	Oncofetal driver
C17orf96	2.195099931	0.306284263	0.633780058	2.634774976	Oncofetal driver
NSUN7	5.528522005	0.161141227	0.452978074	2.598087512	Oncofetal driver
DLG3	3.40581389	0.653898444	0.664391367	2.091661582	Oncofetal driver
CRTC3	3.462894148	1.549959857	1.409523744	3.730510446	Oncofetal driver
MTMR3	4.961082491	2.194400459	2.06773988	4.539197529	Oncofetal driver
ADARB1	7.161217893	0.958114414	1.242625985	3.780143651	Oncofetal driver
IPO7	40.09227206	13.95770307	15.21715683	33.0912128	Oncofetal driver
BCL10	6.080154884	2.161197631	2.66272407	4.977499922	Oncofetal driver
RDH13	2.577742625	0.85694798	0.791507017	2.181608191	Oncofetal driver
PDP1	6.111769331	1.040035644	1.277570742	2.400008326	Oncofetal driver
VASH1	2.49607502	1.028635089	1.26487555	2.695312558	Oncofetal driver
RAD21	39.56912781	11.08375141	14.62349823	46.04320557	Oncofetal driver
HLTF	13.237407	4.867516549	5.316854737	17.33823093	Oncofetal driver
DBN1	14.76305423	0.752209628	1.247597611	4.939149967	Oncofetal driver
SMC5	19.92564971	8.587291614	10.76403652	21.01635393	Oncofetal driver
CENPH	2.830550291	0.559743931	1.040385254	4.407154906	Oncofetal driver
C1orf52	7.803208143	2.253767096	1.885962957	4.767001619	Oncofetal driver
ODC1	37.172244	7.409514747	12.97562574	35.00206653	Oncofetal driver
PSPC1	12.73438617	4.282874142	6.441515376	14.22706596	Oncofetal driver
ZBTB45	2.162299525	0.613355958	0.550096157	3.408584488	Oncofetal driver
LRIF1	7.657219208	3.252744226	3.831175403	8.502542432	Oncofetal driver
TMEM117	3.030180267	1.291061515	1.349769115	5.851867224	Oncofetal driver
MAP4K3	18.39875126	5.142720491	5.365564518	12.38538426	Oncofetal driver
CPD	26.00375763	8.248977815	8.420529497	49.21881868	Oncofetal driver
TMEM164	7.475663368	0.966533775	1.058071501	7.178421536	Oncofetal driver
LAMC1	223.5739509	7.506430257	7.589214043	52.87956668	Oncofetal driver
AAED1	6.196659278	1.682552337	3.153173277	4.955246155	Oncofetal driver
SEC61G	9.565263647	2.414607448	3.268109793	8.041318472	Oncofetal driver
ZNF622	20.13114719	8.083787074	10.60681924	25.90418183	Oncofetal driver
CDKN2B	10.42587743	0.355196279	0.503994147	5.397958583	Oncofetal driver

SLC16A3	11.62219011	0.119037716	0.223081647	2.858102237	Oncofetal driver
NFYA	11.05812454	4.479174607	4.824870064	9.793005455	Oncofetal driver
AP3B1	12.4613474	3.974587364	5.154855984	10.34259964	Oncofetal driver
PDGFA	10.35278996	0.650929508	1.666843977	6.700557427	Oncofetal driver
TMF1	26.22530707	5.049797228	6.313589563	13.90873696	Oncofetal driver
RAB23	10.51308358	1.766291102	2.233697471	4.711620622	Oncofetal driver
ARID3A	57.72881805	0.974907861	1.570958665	3.988618674	Oncofetal driver
ARMCX6	10.71562286	2.964078164	3.97215188	7.899100321	Oncofetal driver
RBM12B	3.904277043	0.775752777	1.020694438	5.533486172	Oncofetal driver
EEF1E1	2.260947385	0.742730857	1.080560635	3.493737418	Oncofetal driver
MOSPD1	33.34606774	1.616508226	2.401884488	5.696160586	Oncofetal driver
PAK4	11.07440729	3.694487428	4.416794352	23.89023925	Oncofetal driver
LTBP2	3.46464749	0.721025011	1.407186835	2.802255746	Oncofetal driver
ST20	2.977224677	0.408252505	1.028079244	4.810440456	Oncofetal driver
HMGN1	38.86962624	10.79607127	19.03347764	78.43016087	Oncofetal driver
HOMER1	2.968508171	0.324561239	0.909226691	2.137857398	Oncofetal driver
NSD1	5.620295423	2.714425738	2.492630144	8.33763157	Oncofetal driver
KDM6A	5.446158524	2.221009116	2.393018121	6.17484922	Oncofetal driver
PROSER1	6.50862607	1.892960121	2.188939446	4.719238976	Oncofetal driver
STAM	10.28487143	2.500760308	3.082027792	8.105254676	Oncofetal driver
PFKP	37.91487705	0.57813687	0.708724643	4.926515149	Oncofetal driver
PI3	2.369164835	0.178433585	0.167195992	3.257334595	Oncofetal driver
CHD3	11.65019509	2.747467609	2.770186393	9.442444207	Oncofetal driver
CERS5	5.893106818	2.001329408	2.531328542	4.704626308	Oncofetal driver
TCEAL3	27.89188965	3.671582598	5.039149368	13.58018492	Oncofetal driver
NT5DC2	16.93497946	0.361020821	0.419798267	3.384103738	Oncofetal driver
CCDC97	6.35699688	2.971739054	2.842714711	19.16586859	Oncofetal driver
CNTNAP2	6.980436163	1.326404786	1.448765919	3.370978206	Oncofetal driver
OLFML2A	3.565641783	0.763090563	0.21736966	3.131010733	Oncofetal driver
CSTF2	2.203284637	0.938748111	1.013482779	3.747610415	Oncofetal driver
ZNF552	7.180009785	2.442582811	2.198513551	6.811372064	Oncofetal driver
HK2	13.48254147	0.410873932	0.404088228	3.069891544	Oncofetal driver
MAU2	4.677129792	2.219224791	2.19333511	5.405661133	Oncofetal driver
LDB1	14.45650298	6.369260374	6.943224051	14.26693372	Oncofetal driver
PRR14L	7.871670037	2.519632115	2.472051281	7.741839056	Oncofetal driver
ZNHIT6	6.10749944	2.744612025	2.590439778	5.863587734	Oncofetal driver
KLHL7	5.461785694	2.079178644	2.105473101	5.172307023	Oncofetal driver
WNK1	23.38451274	10.50912477	11.97408592	39.6642526	Oncofetal driver
LEPREL4	12.59838412	0.740854678	0.323251707	3.158648787	Oncofetal driver
BMS1	19.13332775	8.019077366	10.58817699	24.15989606	Oncofetal driver
RAB11FIP4	3.794911077	0.791043963	0.78512125	3.020828877	Oncofetal driver
HAUS2	3.477883053	1.635961497	1.733282609	3.793069327	Oncofetal driver
PPME1	3.432419382	1.016947477	1.125934174	2.31463308	Oncofetal driver
CD200	6.042577318	0.476149867	0.454580515	2.303861082	Oncofetal driver

SMARCA4	19.21626186	5.779565572	7.957510373	16.10222929	Oncofetal driver
SUV420H2	3.09085697	0.404773816	0.509786975	2.497122647	Oncofetal driver
ATP9A	18.7158132	2.708526139	2.793131969	12.81633794	Oncofetal driver
SLC22A17	6.807249927	0.977096974	0.536707731	4.152777101	Oncofetal driver
GMCL1	9.606532061	4.655300707	4.448337149	21.07493088	Oncofetal driver
TP53BP2	11.84215324	2.73587442	5.10137827	8.862521821	Oncofetal driver
ASAP1	10.95245186	3.395389802	4.512441671	8.789901609	Oncofetal driver
DLG5	8.474876796	1.01729651	0.881116236	3.151599212	Oncofetal driver
ST6GALNAC2	13.91311788	1.183629228	0.70578154	6.090618828	Oncofetal driver
SEMA5A	13.65977377	3.916709386	2.151621817	6.127772794	Oncofetal driver
FUNDC1	10.49183487	3.606041999	4.109890204	9.388134126	Oncofetal driver
YOD1	5.338196324	1.215707063	2.441020454	6.958452217	Oncofetal driver
TTC7A	4.383480641	1.514192181	2.110196755	4.353246693	Oncofetal driver
POLR2A	15.4298325	6.061756098	8.204689419	16.50050096	Oncofetal driver
WASF1	9.403104678	1.490653876	2.207612792	4.111195423	Oncofetal driver
SMAD5	13.01849564	3.772010375	4.310361425	17.15451273	Oncofetal driver
RSPH3	6.264490069	1.966358489	2.289375672	6.630092176	Oncofetal driver
FAF2	13.1657608	6.135178402	6.604303448	12.78076061	Oncofetal driver
UHRF1BP1	7.160775164	1.694321511	1.873777231	4.554964652	Oncofetal driver
SAE1	13.63883186	5.76952499	7.162810485	33.68274243	Oncofetal driver
ZNF597	3.205062348	1.327015739	1.549032967	3.556014667	Oncofetal driver
DVL3	17.97781975	4.628801597	4.828356678	10.26153999	Oncofetal driver
LSM14A	53.61828193	23.7356026	24.58861734	98.27229242	Oncofetal driver
CDV3	91.17750867	35.12345203	37.94819128	73.78504685	Oncofetal driver
CCDC127	7.592587548	3.777896144	3.805730821	8.025336625	Oncofetal driver
C10orf2	2.483737366	1.085911805	1.375029749	4.790041226	Oncofetal driver
HMGA1	36.04064531	4.446107601	5.320014127	30.84679296	Oncofetal driver
ANKRD13C	7.145987152	2.67081099	3.077868983	6.641829969	Oncofetal driver
C9orf40	3.489127496	0.672511352	0.837445707	4.740339371	Oncofetal driver
C6orf228	5.314363543	2.148129747	2.778684805	8.946194746	Oncofetal driver
42070	36.48646972	16.46995824	18.39524174	42.19762889	Oncofetal driver
ISYNA1	5.52917865	1.669219171	1.446048416	5.393493949	Oncofetal driver
DSTYK	3.741893364	1.775824832	1.412679273	3.539562195	Oncofetal driver
PIK3C2B	2.626983157	1.054278269	1.045267993	2.319714422	Oncofetal driver
RRP12	2.755135542	0.753891736	1.114939869	2.279918689	Oncofetal driver
CHD8	10.75881895	4.140983611	4.596257297	9.848017139	Oncofetal driver
BMF	5.602357192	1.394926746	1.455848277	8.338684869	Oncofetal driver
BMP4	9.46577671	1.832402577	0.458351545	2.927621002	Oncofetal driver
ZMYM1	5.33965199	2.025856872	1.938233731	6.159090966	Oncofetal driver
SASS6	2.857580504	1.099028695	1.165825827	5.14304633	Oncofetal driver
FMO1	5.644579111	0.558855587	0.377898495	3.806343077	Oncofetal driver
LEMD2	5.093641642	2.241738835	2.734418027	5.122336201	Oncofetal driver
C9orf86	8.488776548	1.112940169	1.067020132	2.320570692	Oncofetal driver
C6orf211	23.24126601	8.25863541	10.24886259	19.24712812	Oncofetal driver

RPL39L	3.256642152	0.271959717	0.746293504	8.781048265	Oncofetal driver
TLE1	24.92843401	8.683910874	9.419105126	24.02119857	Oncofetal driver
ETV3	6.003369988	1.710051932	2.557438064	11.52266473	Oncofetal driver
MED15	6.48081206	1.641502984	1.898939676	3.705406122	Oncofetal driver
KLHL22	2.185211981	0.700875395	1.332341423	3.49715461	Oncofetal driver
ATAD5	2.701130348	1.207825135	1.333564682	6.948043225	Oncofetal driver
CDK1	3.076722879	0.105352959	0.542948559	6.181828948	Oncofetal driver
ZBTB26	3.534532935	1.90618544	1.09245033	3.757230016	Oncofetal driver
STX7	19.16793103	7.262193485	9.409417578	19.31829062	Oncofetal driver
SLC3A1	6.346004976	0.961470744	1.987927577	13.60230817	Oncofetal driver
GJC1	3.36284751	0.218607389	0.108589712	3.088052516	Oncofetal driver
NOMO2	7.864147388	1.932384205	4.559698952	6.864604884	Oncofetal driver
DCAF7	25.56500659	8.905162649	9.193394649	25.41694254	Oncofetal driver
CTBP2	7.433757143	1.383298863	0.97916548	2.6890064	Oncofetal driver
SLC1A5	16.81443147	1.612102361	1.348081567	3.112649643	Oncofetal driver
HNF1B	5.07527001	2.469842905	2.482606638	13.45264798	Oncofetal driver
LPCAT1	17.97995442	1.724920271	2.555158453	5.040701042	Oncofetal driver
C5orf24	20.38153126	6.190641506	10.45884463	18.72295316	Oncofetal driver
RHOU	31.27681507	8.223306862	13.07183542	22.01142646	Oncofetal driver
HEATR6	4.221603891	1.573852413	1.528806069	4.520561949	Oncofetal driver
BEX2	4.332663304	0.33268153	0.574238614	7.705602474	Oncofetal driver
GFPT1	48.36598159	4.065711369	6.323288982	18.77279441	Oncofetal driver
EIF2AK3	8.588809351	1.500575884	2.208525514	4.33990153	Oncofetal driver
SCD5	4.542039024	0.963729557	0.758888315	2.256652819	Oncofetal driver
SMC3	54.75461392	24.85398279	23.03595475	67.68438323	Oncofetal driver
BAG3	18.09085462	2.076364143	4.375836207	8.69089812	Oncofetal driver
AGAP1	2.922860804	0.861737264	0.858768868	3.77396687	Oncofetal driver
PEA15	48.19078317	12.41638306	14.26796727	60.56183287	Oncofetal driver
POLD1	4.419734036	0.681195843	1.035829142	8.739542238	Oncofetal driver
NCAPD2	6.085638544	1.641077367	1.796489614	9.947415462	Oncofetal driver
CENPF	2.173693455	0.213627671	1.020719845	15.94992493	Oncofetal driver
SMAD3	10.9034938	3.346386495	3.366413225	14.25033035	Oncofetal driver
CNN1	13.61327491	1.420827558	0.231976791	2.432804799	Oncofetal driver
ALPK3	9.882807508	0.367028425	0.382713764	10.04820163	Oncofetal driver
IREB2	12.26199607	4.929624918	5.279806806	13.43812364	Oncofetal driver
WDYHV1	3.095826352	0.97547599	1.755683387	3.358316915	Oncofetal driver
NCK2	26.15700123	3.505330766	3.000636825	24.08095745	Oncofetal driver
MAP4K4	40.62457942	11.20924506	12.55037176	38.56725774	Oncofetal driver
C3orf58	15.87926131	3.054723144	3.400346575	12.20479968	Oncofetal driver
CNOT3	6.342229139	2.401337183	2.630402138	9.257956284	Oncofetal driver
SPTY2D1	10.7774952	3.686191244	4.787457448	9.218188453	Oncofetal driver
TTYH3	18.35005472	0.994453545	3.809144248	9.473416678	Oncofetal driver
HIVEP2	4.582942488	1.649574134	2.003666226	8.973722413	Oncofetal driver
FUT11	6.720572681	1.352784294	1.833646993	3.983475019	Oncofetal driver

ZNF585A	2.266124313	0.926112995	1.04319099	2.210714546	Oncofetal driver
EPRS	33.879586	10.9213184	13.18984853	27.27004359	Oncofetal driver
SULF1	5.300666465	0.882418971	0.668862195	4.380261787	Oncofetal driver
NDRG1	42.20183868	15.42194048	6.222611669	69.39304378	Oncofetal driver
SLC1A4	4.734056654	1.590560939	1.304090182	5.89438098	Oncofetal driver
SOS1	24.91524207	4.83618756	6.473431817	11.93162409	Oncofetal driver
BUB1	2.009875145	0.088755326	0.383841196	3.293900922	Oncofetal driver
SLC6A8	4.515655981	0.213477708	0.319351034	8.407840814	Oncofetal driver
SUPT16H	19.65312267	8.966733058	9.492065519	30.18452871	Oncofetal driver
MUC13	2.500162187	0.170808306	1.258331572	10.54098206	Oncofetal driver
FAM220A	4.237578123	1.642140303	1.627337086	3.708889444	Oncofetal driver
ADM2	3.216307314	0.125203959	0.293296804	2.642485303	Oncofetal driver
EML2	3.946431147	1.62033646	1.415045408	8.25409182	Oncofetal driver
CCNJ	3.971344776	1.027576133	1.255904703	2.826515935	Oncofetal driver
BLMH	7.532949316	3.064387141	1.919280845	5.106007439	Oncofetal driver
ZDHHC9	20.63191814	5.969612498	9.034483082	19.92521036	Oncofetal driver
FAT1	12.37337095	1.19079697	6.224621557	8.91642436	Oncofetal driver
C5orf22	8.036006495	3.514132274	3.695167672	7.985632075	Oncofetal driver
KDELR1	197.2962082	38.24653421	38.61361896	97.21607597	Oncofetal driver
ZNF649	4.597013877	1.148922019	1.3038385	4.147589056	Oncofetal driver
SESTD1	16.07913747	2.319136963	1.870455709	9.998763672	Oncofetal driver
C20orf112	5.06552625	1.451499048	1.562259667	3.758338917	Oncofetal driver
CHD2	22.05840791	9.669370753	10.96932362	25.4923108	Oncofetal driver
ZNF529	3.363457989	1.437063303	1.811664778	3.679834629	Oncofetal driver
ZNF284	2.426434392	0.503590005	0.56799689	4.327008067	Oncofetal driver
ACTR8	8.483224419	2.339682959	2.301473796	5.23875964	Oncofetal driver
SMC4	23.28300821	5.760773769	7.855111649	46.96932526	Oncofetal driver
KRT10	7.508676059	3.189508738	3.806196283	8.627565959	Oncofetal driver
ZDHHC20	7.106547092	1.922251235	2.753650973	5.478869994	Oncofetal driver
ZNF165	2.221626561	0.61568479	1.437695082	2.527384515	Oncofetal driver
ZNF805	2.43337226	0.821944655	0.964150368	2.919792751	Oncofetal driver
VPS35	17.14552593	6.419010133	7.405817737	14.43556879	Oncofetal driver
FUBP1	21.02905686	8.016990124	9.333921317	38.23379498	Oncofetal driver
BTBD3	10.73968212	1.491112286	2.001315617	4.13509621	Oncofetal driver
PRRC2A	18.51945028	5.884823818	7.207623846	14.30857161	Oncofetal driver
SLC35F5	11.58995418	3.042358538	3.516793077	8.790631343	Oncofetal driver
SENP1	3.86524442	1.326171325	1.894283835	4.020853863	Oncofetal driver
ZNF526	3.122027688	0.864477778	0.756356778	5.664431267	Oncofetal driver
HSPA5	220.9412035	96.07487013	118.2745814	217.0377187	Oncofetal driver
ZCCHC11	10.21099952	3.714278093	3.842446487	11.47538279	Oncofetal driver
CEP350	14.89053112	4.423206503	4.805727914	20.02638043	Oncofetal driver
PRPF4	8.304755291	2.871351055	4.712118895	9.509571537	Oncofetal driver
YTHDF1	19.7875725	4.44942236	5.045459462	10.97704305	Oncofetal driver
WDR37	3.527721913	1.430755949	1.746595072	3.449578108	Oncofetal driver

E2F3	2.903937029	0.845387978	1.705539203	7.709787118	Oncofetal driver
ASCC3	10.05449885	2.868133936	4.601975959	13.13572195	Oncofetal driver
CCT6A	41.72181852	16.08871739	23.12263561	50.26727208	Oncofetal driver
PSPH	7.204041451	1.968170252	2.291299391	5.986592028	Oncofetal driver
DICER1	13.6901349	4.522412282	5.447027066	14.46436531	Oncofetal driver
BCL2L12	7.053036732	2.854340661	3.37183994	21.84377749	Oncofetal driver
PRMT1	9.924257072	3.258704739	3.846009813	8.794093504	Oncofetal driver
PROM1	52.66708872	0.681317512	0.63323942	2.89838158	Oncofetal driver
DYNC1H1	21.99045453	4.265011421	5.513039026	11.51963604	Oncofetal driver
PKIB	37.79429431	1.27828913	1.168921325	2.71108108	Oncofetal driver
PMM2	4.121677873	0.964246226	1.65560992	3.078911097	Oncofetal driver
FRAS1	5.875751945	0.286713409	0.157733898	2.111207434	Oncofetal driver
PRKAB2	45.65292189	21.27761026	21.25919694	60.34635626	Oncofetal driver
ABCA1	31.44124736	9.029302227	10.42918511	25.25295281	Oncofetal driver
SP3	12.49752247	5.009302865	5.942983396	14.82455021	Oncofetal driver
ZNF525	6.004883331	0.677323209	0.643242543	3.116357869	Oncofetal driver
PCGF2	6.253198158	1.102578274	1.714570846	4.276126945	Oncofetal driver
PARM1	114.058606	1.12895929	0.49826664	2.742400325	Oncofetal driver
CEP250	5.342718066	1.925420198	2.28469586	5.734254253	Oncofetal driver
HNRNPA0	26.00498505	9.444883847	11.88900528	23.04640735	Oncofetal driver
FLYWCH2	4.896081142	1.297323277	1.427645539	5.081453332	Oncofetal driver
KIAA1217	17.79019013	6.554567533	6.076582667	13.69867744	Oncofetal driver
SHC1	25.18987954	8.128368968	8.674044871	18.02041184	Oncofetal driver
SPSB1	56.87599675	1.34935221	2.908620671	4.918113506	Oncofetal driver
KCNJ16	8.46338383	0.555729374	0.685171074	5.598993672	Oncofetal driver
DMPK	6.460699991	1.316648379	1.335643575	6.400793787	Oncofetal driver
TUBG1	16.03277181	5.409293329	6.979253099	16.40511536	Oncofetal driver
ZNF211	4.454920722	1.87760132	2.022377592	6.120700442	Oncofetal driver
PPP1R2	11.56245139	3.81869446	4.853704853	9.47699613	Oncofetal driver
TSPYL2	15.95696735	5.667999814	6.617345672	15.5375437	Oncofetal driver
TCF7L2	21.59149232	6.961623967	6.966473159	17.05252094	Oncofetal driver
LSM11	3.571582795	0.706807302	0.457158129	2.378850321	Oncofetal driver
CSTF3	13.54526656	3.391604913	5.309488791	10.71929109	Oncofetal driver
YAE1D1	4.826675799	2.364505466	1.689857502	4.920885302	Oncofetal driver
TPX2	5.465810876	1.268031715	3.056064453	23.06371884	Oncofetal driver
PNMA1	7.76086869	1.359724865	1.588952024	4.559871089	Oncofetal driver
HSPA13	15.30618706	1.762578357	4.177236774	11.35811167	Oncofetal driver
TRNP1	23.95464667	1.531837881	1.43536405	3.793296285	Oncofetal driver
TOPBP1	9.272557471	2.993077551	4.397883329	16.36810932	Oncofetal driver
HIST3H2A	3.925304323	0.249087529	0.194500176	2.751737849	Oncofetal driver
COL6A3	23.27818328	2.770692647	2.24009012	6.179395318	Oncofetal driver
MICAL3	2.113740039	1.045170277	1.026803665	3.585209062	Oncofetal driver
MMGT1	11.17434249	3.531027852	3.860948165	9.918411219	Oncofetal driver
GSK3A	7.616911867	3.368658295	3.600751678	11.98997887	Oncofetal driver

NPTN	31.26137979	7.106574433	8.005130637	22.12568565	Oncofetal driver
LMNB2	4.204473702	0.716447635	0.916413788	2.595013892	Oncofetal driver
HAUS6	4.057845098	1.687380639	2.157608203	9.328469336	Oncofetal driver
RHPN2	12.33942183	2.865688138	3.992911278	13.27052578	Oncofetal driver
ZNF614	7.807056546	0.929617889	1.031827012	4.647504567	Oncofetal driver
UBE2S	3.983347195	0.554179599	2.515252062	6.623990604	Oncofetal driver
CCDC149	2.300881403	0.908689004	0.817321804	2.128492382	Oncofetal driver
GOPC	14.58856436	5.9335541	8.001903667	15.35288829	Oncofetal driver
GXYLT1	8.267952845	3.639240677	4.49868503	12.30210347	Oncofetal driver
IMPA2	12.89810481	3.099575762	4.301603236	7.537153448	Oncofetal driver
PURB	8.892670049	3.15739853	4.740062617	13.08178358	Oncofetal driver
ZNF720	2.679762632	0.9397793	1.56855594	3.863616852	Oncofetal driver
PRLR	8.428331912	4.416639223	3.094001826	20.68752968	Oncofetal driver
NCOR2	13.68959532	2.859934781	3.508201409	7.264000436	Oncofetal driver
KLF5	14.47211912	0.942463348	1.19566845	27.47800979	Oncofetal driver
SPARC	1197.625884	46.31003443	37.71018203	121.9428177	Oncofetal driver
FLVCR1	15.16068534	0.773529812	1.24296542	2.80639979	Oncofetal driver
MAFG	4.668851956	1.218906483	1.703970582	3.000450289	Oncofetal driver
ADCY6	5.526876355	1.610679163	1.566581919	3.644015386	Oncofetal driver
WDR27	2.371574493	0.845005385	0.902750791	2.750229623	Oncofetal driver
ACLY	35.00216038	5.252625909	12.21847345	20.84423024	Oncofetal driver
ZNF549	2.36750803	0.545531364	1.234534082	3.674140138	Oncofetal driver
42249	53.18145189	17.66358512	21.4179017	50.86336653	Oncofetal driver
TAOK1	10.61859361	3.048664054	3.607815918	11.14185968	Oncofetal driver
RSL1D1	52.98574356	28.13879122	23.75560071	57.40446975	Oncofetal driver
RUNX1	2.254724428	0.737940943	1.315042721	2.920225272	Oncofetal driver
RNPS1	7.594328602	2.329770439	2.841778564	7.227092522	Oncofetal driver
MEX3D	4.350517023	0.882378209	1.280872812	2.81354884	Oncofetal driver
PLOD2	39.48130441	8.747100926	12.15300999	50.97974819	Oncofetal driver
MFAP1	32.44598203	10.18715161	12.93612227	34.70779136	Oncofetal driver
LRRC16A	11.38475303	0.949334302	1.432551146	4.509131761	Oncofetal driver
ARHGAP5	31.11561093	8.433574002	9.83949716	21.24649052	Oncofetal driver
RARS2	50.28992546	13.67355143	15.17776963	29.76857638	Oncofetal driver
ENAH	22.71348031	3.526421608	4.468029651	9.605854493	Oncofetal driver
RSL24D1	30.95249504	11.05863641	10.83667845	26.57610061	Oncofetal driver
C11orf9	19.02724726	1.500591303	1.648759984	4.368184055	Oncofetal driver
ERO1L	25.00397034	5.876195255	8.499461839	21.64259616	Oncofetal driver
SKP2	37.51991258	6.294896931	6.552156871	19.96645003	Oncofetal driver
ZNF432	8.586390032	1.197984283	1.848883258	6.363260621	Oncofetal driver
BIN1	33.33966841	3.057927778	3.32201472	8.508812567	Oncofetal driver
ZNF845	3.840405268	1.447261524	1.58437109	6.975577124	Oncofetal driver
KCTD15	5.851817645	2.232734317	1.157508626	11.0468139	Oncofetal driver
TCF7L1	10.10742496	5.070836713	2.683908542	15.45046422	Oncofetal driver
RIOK2	7.092306046	3.042652609	3.436664683	6.827929197	Oncofetal driver

FAM222B	10.22344753	2.28832986	2.752847447	6.640233033	Oncofetal driver
H2AFZ	12.20730322	4.023472029	5.537824543	12.231809	Oncofetal driver
PMEPA1	10.39236878	1.96054656	1.524598922	30.41441873	Oncofetal driver
CANT1	6.827014296	3.313209998	3.025751277	10.93458798	Oncofetal driver
ZNF124	4.331672921	0.875252881	1.136222058	2.045779677	Oncofetal driver
ZNF765	4.927849279	0.554941105	0.804618259	5.204914861	Oncofetal driver
LUZP1	5.06304026	1.695674203	1.891998013	5.068156782	Oncofetal driver
GGA3	3.919884408	1.678126668	2.096586259	5.460312357	Oncofetal driver
EHMT2	2.869991292	1.415311991	1.365882754	4.015121508	Oncofetal driver
CDH2	57.16579368	15.94052835	20.51444827	48.4967846	Oncofetal driver
FZD6	15.06499427	0.886461563	1.803660178	5.273073902	Oncofetal driver
MOB4	11.55180094	3.992667451	5.560169642	12.70821204	Oncofetal driver
KDM3A	5.763773803	1.962019706	2.133123405	9.024591657	Oncofetal driver
MOB3B	10.96052334	0.752948812	0.744392616	7.259498422	Oncofetal driver
C19orf54	4.576446142	1.818500671	1.91777957	5.882960976	Oncofetal driver
PAFAH1B2	15.14989379	4.975997821	6.271161165	11.93258925	Oncofetal driver
MMP14	41.4152085	3.556416388	4.231459379	16.72094256	Oncofetal driver
CENPQ	5.823958605	1.675619183	1.769115638	14.24549441	Oncofetal driver
HIF1A	88.89110534	9.442907793	17.86194799	32.10670196	Oncofetal driver
CHD4	40.65777858	16.37601087	18.91234976	52.52117385	Oncofetal driver
SMURF2	8.283707448	1.981917533	2.078841125	7.669334931	Oncofetal driver
THOC5	4.480028424	1.926344344	1.655626578	4.033028275	Oncofetal driver
CARHSP1	7.714641682	4.043977587	3.329983315	8.63517457	Oncofetal driver
CDYL	7.974364718	1.964709611	2.304376929	5.310347787	Oncofetal driver
CHST3	10.95540027	0.821286017	0.55364182	2.076970851	Oncofetal driver
QSER1	8.935926889	3.279120582	2.911466615	9.077746232	Oncofetal driver
TGIF2	6.078913032	1.712926651	1.904785885	4.658844283	Oncofetal driver
FAM108C1	2.756302678	1.16950498	1.063932585	3.775398209	Oncofetal driver
SPAST	7.308022436	2.662285227	3.197847048	6.867381222	Oncofetal driver
MTHFD1L	4.772123377	0.137413707	0.756949845	2.393581806	Oncofetal driver
STK35	4.842153733	1.707423516	2.048408031	3.88189066	Oncofetal driver
ACBD6	9.611794088	4.387436478	4.569730445	12.58344314	Oncofetal driver
COL6A1	87.82922381	17.57967111	15.1211867	44.13363776	Oncofetal driver
PPIH	7.378502867	2.159659445	3.305869895	8.375372141	Oncofetal driver
USP51	2.685181633	1.238184132	1.006969823	2.817791349	Oncofetal driver
UBE2T	2.56715138	0.270682911	1.068892609	7.731381607	Oncofetal driver
CAMTA1	4.262424422	1.914937113	2.105574333	4.80690145	Oncofetal driver
SULT1C2	2.022150274	0.117678214	0.457148359	9.275985998	Oncofetal driver
ZNF417	4.29161194	1.631488558	2.151266473	6.181965705	Oncofetal driver
UGCG	36.24530515	4.131632567	10.50636425	17.3422776	Oncofetal driver
C14orf118	3.262537197	1.173457879	1.274184478	3.574959057	Oncofetal driver
HKDC1	2.916766035	0.109934638	1.675049239	6.648033112	Oncofetal driver
ZNF134	5.617012164	2.032911251	2.521646635	7.924535397	Oncofetal driver
STXBP6	2.084832304	0.510505411	0.322889098	3.175794035	Oncofetal driver

NEDD9	17.0286011	0.99189368	2.821801565	5.19248833	Oncofetal driver
SRP68	32.42048946	13.31349483	14.54709663	36.51229402	Oncofetal driver
ASPHD1	9.531932401	0.106874926	0.150216053	6.590497218	Oncofetal driver
SNRPD2	42.12524845	18.57248714	17.55858749	49.03355737	Oncofetal driver
ZNF616	7.720059462	2.622915124	2.78711217	7.485196122	Oncofetal driver
RACGAP1	2.946511711	0.349157708	0.854036094	6.760230566	Oncofetal driver
BRD3	6.501634246	3.000719076	3.261100631	7.676630217	Oncofetal driver
NRM	2.148544646	1.061193517	1.080410887	13.58231585	Oncofetal driver
CSNK1G1	3.809899718	1.494726751	1.505985726	6.958509695	Oncofetal driver
SPP1	28.64831663	4.490833319	6.28249169	105.478342	Oncofetal driver
MBOAT7	8.925329148	1.416596071	2.552653979	4.338788677	Oncofetal driver
PCGF3	10.96098466	3.946306863	3.178056905	8.184501008	Oncofetal driver
CHSY1	7.713182527	1.260309658	2.222939182	6.714449865	Oncofetal driver
SNRPA1	9.453830681	3.513249816	3.849417705	11.78198076	Oncofetal driver
KIF21A	28.13767071	6.672258663	9.038552015	16.36862088	Oncofetal driver
CDK8	18.20328161	3.050771153	3.965738096	7.077151978	Oncofetal driver
LPHN1	2.356588405	0.462606266	0.366973219	2.713307011	Oncofetal driver
ADNP	15.51748667	6.746350473	6.115894829	17.55980016	Oncofetal driver
DHX15	18.75656704	5.782297515	7.110709095	18.56158772	Oncofetal driver
GPATCH3	3.8414619	1.341900756	2.107210533	3.730807088	Oncofetal driver
ZNF652	8.515294902	3.970999946	4.356026819	11.87565283	Oncofetal driver
SGPP1	17.64376348	6.776704999	9.315148996	22.01673582	Oncofetal driver
PLP2	19.72440446	3.735079509	4.646349155	26.81825459	Oncofetal driver
SIPA1L2	16.53690579	1.061765068	0.734808987	4.049774853	Oncofetal driver
COL4A1	326.2193782	5.31968576	9.501465687	61.32401479	Oncofetal driver
RP11-382A20.3	16.95404556	2.362169396	2.256802039	5.363621686	Oncofetal driver
PHLDB1	2.174716551	0.47853927	0.584413408	2.363863421	Oncofetal driver
NBL1	10.06878455	1.106927702	0.830886762	3.492018694	Oncofetal driver
TUBA1C	14.20279911	1.998434907	2.889280134	5.382205254	Oncofetal driver
SH3RF1	8.426318363	2.237063485	2.655383907	5.43058028	Oncofetal driver
PRR12	6.195970454	1.723351767	1.934444617	10.6588042	Oncofetal driver
ARL5A	15.99683564	5.133251952	7.322632007	13.64665499	Oncofetal driver
FBLIM1	11.50973021	1.034801287	1.55877291	3.89993097	Oncofetal driver
SCAF1	13.20613708	4.856513624	5.168147527	18.64911468	Oncofetal driver
MAFF	9.601241801	1.224268971	7.148721004	10.5673328	Oncofetal driver
RLIM	13.34344117	3.263535155	4.312978142	9.650612031	Oncofetal driver
MAP3K1	10.30243556	2.548552376	3.095708759	9.424320366	Oncofetal driver
UACA	56.76480494	11.09548592	10.37221425	29.46185959	Oncofetal driver
BORA	2.606642964	1.065581441	1.448487606	3.498098901	Oncofetal driver
APPBP2	11.99995094	4.921776508	5.649911351	13.58815107	Oncofetal driver
OTUD3	6.796954343	0.626835313	0.832565375	3.167584663	Oncofetal driver
NPNT	69.0927325	1.753806412	1.283771112	3.17131598	Oncofetal driver
RP11-1407O15.2	2.447613374	0.858319926	0.970390271	2.813284615	Oncofetal driver
MSRB3	8.73889076	2.129882612	1.442666681	4.006674761	Oncofetal driver

XPR1	12.68992333	1.824832641	2.86067966	10.66590966	Oncofetal driver
CSNK1E	10.33087201	2.640374907	2.514409079	6.960599336	Oncofetal driver
MANF	33.70604573	12.23183781	15.92177886	45.13114196	Oncofetal driver
FAM168B	23.9756452	7.209773986	7.078761428	25.37181396	Oncofetal driver
DCUN1D2	4.911969529	1.302942897	1.913459253	4.427592347	Oncofetal driver
TMCO3	34.50165591	2.427103832	2.546737336	17.9569511	Oncofetal driver
PERP	113.5513471	27.70438537	46.08955165	89.06479203	Oncofetal driver
ANKZF1	8.247889322	3.90691693	3.664015861	8.801456279	Oncofetal driver
EHD2	23.82362286	3.832634065	2.633941853	21.74917564	Oncofetal driver
DSG2	79.73042846	3.995165448	6.327571468	30.91985836	Oncofetal driver
ZNF384	8.262779297	3.160935311	3.752918723	14.48985026	Oncofetal driver
GMPS	13.44340938	4.560099972	5.864776784	17.10798761	Oncofetal driver
KIAA1462	2.462685459	0.90675016	0.924672028	7.482561268	Oncofetal driver
42255	12.03981491	1.838169221	2.200569991	6.566986757	Oncofetal driver
ZNF460	2.312506477	0.88180668	0.762333597	2.001924558	Oncofetal driver
HPS3	6.124944304	1.839913807	2.763394824	5.189548454	Oncofetal driver
ITGAV	45.50421084	3.666348393	6.30928155	18.78054173	Oncofetal driver
AP4E1	2.189716796	0.808651601	1.365999658	2.374603644	Oncofetal driver
STT3A	30.02413738	12.68037698	12.24887349	25.08522488	Oncofetal driver
NEK7	13.65459884	5.180657306	5.480529145	21.30601816	Oncofetal driver
C12orf49	13.6818548	1.022498475	1.311087496	2.660706392	Oncofetal driver
ZNF581	6.887173409	1.411970451	1.149789774	4.127919785	Oncofetal driver
AMMECR1	11.31608179	0.985952356	1.211082985	3.461292931	Oncofetal driver
PTGFRN	7.312761242	1.880182105	3.125622723	7.650824332	Oncofetal driver
EXT1	17.66449646	5.307962991	5.488189847	22.26727996	Oncofetal driver
SLC39A1	25.87222603	7.929465396	11.31748946	29.38500142	Oncofetal driver
TCOF1	7.153257661	2.466858161	2.23450467	6.045577219	Oncofetal driver
USP46	7.028511876	0.771045398	1.195139767	2.118068148	Oncofetal driver
AQP3	49.05953444	9.76817843	9.976689888	21.74052849	Oncofetal driver
FAM63B	9.908459481	2.907689836	3.459280468	14.09370143	Oncofetal driver
ZMIZ1	14.49631988	3.40853525	3.488631974	13.64997452	Oncofetal driver
SPTAN1	33.88751701	11.7052205	14.00585039	29.40819801	Oncofetal driver
COL5A2	64.01229601	2.670299995	4.459609619	7.479106928	Oncofetal driver
WBP5	141.7226483	13.03861095	21.33652731	96.72603848	Oncofetal driver
PPP1R13L	7.972932222	0.939499428	1.05032543	6.470241895	Oncofetal driver
DOT1L	4.388280361	0.999798985	1.593878809	3.33245954	Oncofetal driver
PFKFB3	8.313841381	2.35158312	5.20537861	8.74128809	Oncofetal driver
ANP32B	210.2413736	83.68589016	79.78839683	226.0034359	Oncofetal driver
TBC1D12	2.433594337	1.114523625	1.28097852	3.52281333	Oncofetal driver
IFT52	5.785087845	1.644382713	1.988645699	4.797111964	Oncofetal driver
SLC10A3	4.524429326	0.958589309	0.958675172	2.443799415	Oncofetal driver
SMG9	6.924620491	2.450295796	2.656774861	9.613646024	Oncofetal driver
AVL9	4.980114424	1.61938041	1.819837871	3.55226714	Oncofetal driver
MGA	4.881808237	2.253075336	2.438225148	8.027665749	Oncofetal driver

RCC1	11.71048939	1.735430436	2.808777809	5.574484443	Oncofetal driver
DOCK1	9.74211258	3.833298496	4.640308587	10.05051368	Oncofetal driver
CDK16	11.23871001	1.776956732	2.596642935	5.432504863	Oncofetal driver
C10orf12	2.331761517	0.843234224	1.261656094	2.852268021	Oncofetal driver
FNBP4	20.87850898	8.849977093	11.7305183	23.28167343	Oncofetal driver
LIN37	6.773234782	2.639902018	2.62421285	5.562516585	Oncofetal driver
MRE11A	6.357687134	2.159227942	3.375705028	8.196077793	Oncofetal driver
CDKN1C	187.9640156	2.978005478	1.708593036	6.921821019	Oncofetal driver
DCAF16	13.38669264	2.612384767	2.279593754	7.767990642	Oncofetal driver
FZD5	29.4854089	8.300343089	7.190823432	30.93711155	Oncofetal driver
CTGF	97.11760795	17.13148557	28.44256148	63.9388938	Oncofetal driver
VPS13A	8.745990176	2.603981783	3.360950709	7.949053908	Oncofetal driver
SYT15	3.091983273	0.955574355	0.773689187	3.19063749	Oncofetal driver
SERPINH1	30.78034483	1.650986341	1.723697447	4.790043822	Oncofetal driver
FNBP1L	48.3347928	6.414576412	8.007165843	25.57348946	Oncofetal driver
SPINK1	233.883531	0.857968156	0.456286899	15.64723241	Oncofetal driver
G2E3	3.808435904	0.868837179	1.116285497	2.40821891	Oncofetal driver
NXT1	3.196903213	0.990139564	1.305766451	3.327327993	Oncofetal driver
C2orf49	7.859763766	2.863252789	3.075452443	9.000595721	Oncofetal driver
AKAP12	34.38692212	10.37688976	10.37011476	32.07624401	Oncofetal driver
ZNF322	4.481519819	1.175895442	1.450754103	3.25115869	Oncofetal driver
NRSN2	7.809864632	0.814730277	1.285758763	5.507496372	Oncofetal driver
TRIM68	3.060239707	0.779023865	1.185061136	2.084899531	Oncofetal driver
CST1	22.48576958	0	0	2.206687654	Oncofetal driver
C11orf84	8.770913481	0.770245726	0.74235735	4.089157622	Oncofetal driver
NOTCH3	5.317939251	0.859881839	0.425576989	5.486951967	Oncofetal driver
GCNT4	3.729329165	0.479423417	0.449229742	13.68795143	Oncofetal driver
AZI1	2.27641503	0.602113271	0.627421187	4.303494342	Oncofetal driver
TNNT1	2.769545532	0	0	3.697075506	Oncofetal driver
AGRN	12.04298759	1.638722368	1.915808648	4.47662146	Oncofetal driver
TOX2	5.037020199	1.219092588	0.999525787	3.408308512	Oncofetal driver
MFGE8	138.3785144	1.697127566	1.094055289	3.178879862	Oncofetal driver
ZNF710	3.035222155	0.730037734	1.571384888	3.331739878	Oncofetal driver
MYH9	83.53783235	34.40837155	44.47981854	90.34948826	Oncofetal driver
PRDM2	5.913625104	2.774692138	3.042111833	6.657673187	Oncofetal driver
MSANTD3	8.245916937	1.497950917	3.086548138	5.207016896	Oncofetal driver
MAP3K7	7.931047942	3.265193878	4.429146052	9.901005541	Oncofetal driver
RBM12	10.78363529	3.499465459	4.632157214	12.73312974	Oncofetal driver
KIAA1429	7.617500967	3.523317006	3.740297428	9.753005511	Oncofetal driver
RBPJ	19.06965156	4.614295278	4.904166714	10.44618666	Oncofetal driver
UBE2C	2.145692563	0.050191921	0.54085509	6.012968203	Oncofetal driver
GK5	3.781291366	0.725518795	1.09720794	4.191147313	Oncofetal driver
ZNF362	2.711948002	1.129893895	1.1744428	2.576287255	Oncofetal driver
YAP1	31.04424133	6.76017857	7.042339781	17.75483372	Oncofetal driver

FNDC3B	18.17103962	5.369607154	6.129702508	14.55984675	Oncofetal driver
WHSC1	2.993304693	0.748639689	1.026381833	5.400225308	Oncofetal driver
GLIS3	6.173047089	0.901703105	0.797058127	7.614546293	Oncofetal driver
CCT8	45.78626498	20.97928475	21.66076836	60.35101686	Oncofetal driver
FOXP1	8.788891957	4.101600292	3.641082891	11.02911661	Oncofetal driver
TTC28	4.220768988	2.146922609	1.790392504	4.423272789	Oncofetal driver
MYO6	26.94674351	6.267611197	9.485781319	24.24168667	Oncofetal driver
FRMD6	6.066734197	1.056241171	1.040947754	2.670906963	Oncofetal driver
UNC5CL	11.62549266	2.799746052	3.321276142	10.05591541	Oncofetal driver
S100A1	15.53646315	0.251610212	0.286740021	6.051832925	Oncofetal driver
FAM126B	2.734136515	1.117334012	1.188641771	3.299828492	Oncofetal driver
COL15A1	7.344103424	0.499501123	0.415715187	4.19042633	Oncofetal driver
FAM199X	5.949527536	2.057190643	3.632742668	8.842496399	Oncofetal driver
IGF1R	19.05381312	0.839867562	0.339926815	2.136610742	Oncofetal driver
HNRNPA1	158.0943066	57.80475108	60.74585464	172.2680507	Oncofetal driver
NEO1	9.32225351	2.843756962	2.890478206	6.530229036	Oncofetal driver
EIF2C2	4.267821439	0.883579146	0.787935372	2.871059869	Oncofetal driver
ESCO1	12.20487	4.034137713	5.52780681	11.67821683	Oncofetal driver
UBXN2A	5.397262094	2.181198904	2.212045242	4.554936909	Oncofetal driver
CDC73	8.475301673	3.528057394	4.675795854	9.506775855	Oncofetal driver
ZDHHC12	3.982408118	1.561984382	1.864805073	3.64441073	Oncofetal driver
ALCAM	31.61894234	11.74839136	18.44781742	33.32686885	Oncofetal driver
SFN	2.530621458	0.015658734	0.058690243	10.10693775	Oncofetal driver
STC1	7.604640113	0.3523687	1.102139535	4.352001123	Oncofetal driver
RPS21	29.93457662	9.294392646	9.80652042	19.31621472	Oncofetal driver
PKM	44.83786858	3.56959593	4.407194276	17.46235666	Oncofetal driver
CCDC99	2.381814127	0.875030533	0.981482821	4.140529876	Oncofetal driver
IFT80	2.900195293	1.199600911	1.156320487	4.481194163	Oncofetal driver
KIAA0947	7.494404983	2.828388223	3.235005458	11.99699024	Oncofetal driver
SEZ6L2	14.58424194	0.085170561	0.098584615	9.320105691	Oncofetal driver
EGLN3	3.651404357	0.176280453	0.38017265	3.517818714	Oncofetal driver
MDM1	4.88103477	1.050156167	0.925094988	3.409686497	Oncofetal driver
SLC44A3	10.31400931	2.095727025	1.681284196	8.625484074	Oncofetal driver
CUEDC1	2.310026147	0.878736616	1.08632725	2.712095327	Oncofetal driver
PRMT5	9.3076655	3.12996286	3.600659959	6.827956175	Oncofetal driver
DCDC2	10.21516602	1.491999067	3.143646075	43.36570399	Oncofetal driver
RAP2A	17.1978922	3.902671034	5.359076683	27.03956593	Oncofetal driver
ZNF682	3.369253314	1.498692841	1.823984247	3.550265891	Oncofetal driver
MAP1B	20.29235697	2.183375408	3.025257089	6.43532027	Oncofetal driver
WWTR1	10.36309752	4.163282063	4.380403815	13.91210126	Oncofetal driver
TTC23	4.31210159	1.561516006	1.90589282	5.392523795	Oncofetal driver
S100A10	508.1726044	20.68465029	32.68671884	144.1382873	Oncofetal driver
LIMS1	14.0562244	3.826092453	4.642703382	8.898862744	Oncofetal driver
NGFRAP1	39.49823934	15.58705143	15.73869146	43.2217791	Oncofetal driver

DNMT3A	4.82755305	1.244607233	1.546573828	6.019365653	Oncofetal driver
TMEM50A	29.75841287	9.196613499	12.58380773	28.31952443	Oncofetal driver
NANP	2.922565219	0.898658876	1.255792053	3.386698251	Oncofetal driver
ZNF865	2.30308432	0.93059018	0.713002192	2.821290176	Oncofetal driver
HSP90AB1	472.5838754	180.0702093	172.2075061	520.4538576	Oncofetal driver
TEAD3	14.64227367	1.93338589	3.021482966	6.434771151	Oncofetal driver
LCORL	2.485986128	0.971115048	1.112167293	3.495100765	Oncofetal driver
PTAR1	17.5318394	4.940208771	6.288082271	17.3257875	Oncofetal driver
ELF4	8.551718945	0.93986873	1.390306724	2.877202613	Oncofetal driver
GPATCH8	13.57718982	5.777526363	5.67185711	21.40168664	Oncofetal driver
C14orf129	7.097000688	1.3274438	2.241463675	3.770976109	Oncofetal driver
ODF2	5.573357425	2.726183271	2.750738271	5.81843065	Oncofetal driver
ZCCHC10	8.918448631	3.246147752	5.538632769	10.44156701	Oncofetal driver
HYOU1	13.28287167	4.863082032	8.16167385	13.59278444	Oncofetal driver
C22orf29	2.734028398	1.026080981	1.001165567	2.400939895	Oncofetal driver
GPD2	4.712547221	1.185149938	1.813647336	3.895769035	Oncofetal driver
TMEM98	22.24796303	2.508415765	1.346295125	7.353317442	Oncofetal driver
ZNF92	5.890746748	2.022668502	1.796110818	6.734159027	Oncofetal driver
ITGA2	11.05351282	0.35924027	0.507362683	3.340660277	Oncofetal driver
UBE2O	3.600335294	1.572995035	1.607267496	5.273997104	Oncofetal driver
KLHL23	3.912605194	2.104350285	1.80720991	11.30314588	Oncofetal driver
DSP	163.9194318	21.50930332	33.00486644	96.972187	Oncofetal driver
TCERG1	13.98563739	4.787450495	5.480110528	14.27374774	Oncofetal driver
H2AFY	5.045080549	1.57366748	2.501853676	4.467385449	Oncofetal driver
BMPR2	14.57212229	4.382108607	4.892959134	10.44896695	Oncofetal driver
ZNF600	12.13904415	3.604274388	4.407123605	9.37844267	Oncofetal driver
ANLN	3.865458727	0.065075429	0.365862207	8.902276975	Oncofetal driver
SPTLC1	23.56459164	5.315770955	6.435726097	13.73760587	Oncofetal driver
RCC2	6.693244171	2.150950704	2.824485732	9.739485326	Oncofetal driver
NNAT	9.454429811	1.082074212	0.894640685	2.887626999	Oncofetal driver
TTC9C	6.819756705	3.07296686	2.378662912	5.747416518	Oncofetal driver
NEDD4L	28.53706086	2.699017398	3.707381829	6.744041365	Oncofetal driver
HUWE1	19.19774147	7.544075029	7.476545108	22.48168262	Oncofetal driver
ZNF445	4.789272177	1.802910577	2.000052603	5.834230683	Oncofetal driver
SNX27	10.46106556	3.218156168	3.467672184	10.99411965	Oncofetal driver
RPAP1	2.765160035	1.098822685	0.931039161	2.127605296	Oncofetal driver
SOD3	12.13417183	2.914617903	1.921855472	9.339933791	Oncofetal driver
TWF1	25.40392908	8.839777703	14.72107129	61.85501746	Oncofetal driver
AGPAT1	16.8086145	3.328850283	4.6070669	9.107665757	Oncofetal driver
SMAP1	10.04374535	2.313728538	2.572292814	5.445578925	Oncofetal driver
ENO1	177.5479649	32.13579725	42.85084311	77.5553679	Oncofetal driver
COX19	6.166582957	1.523350172	1.726819027	4.251290171	Oncofetal driver
TLE3	2.970602049	0.736681855	0.751373546	2.660038724	Oncofetal driver
UCK2	3.757865915	0.829750536	0.706032756	6.059423647	Oncofetal driver

AQP1	40.28801388	4.104477289	3.711991831	34.43691412	Oncofetal driver
DYNLRB1	30.70745167	14.16438941	15.00479142	30.98894162	Oncofetal driver
WFS1	4.500732854	1.49654893	1.415123459	3.857208919	Oncofetal driver
DDX52	7.224730461	2.332935166	3.407532778	6.973320146	Oncofetal driver
GMFB	6.819145388	1.863705871	3.027620109	6.286062272	Oncofetal driver
H2AFY2	4.912049802	1.623159563	3.100366217	5.268646025	Oncofetal driver
PLXNC1	2.931458841	0.976766417	1.213829032	2.539143359	Oncofetal driver
RPL36A	2.007756791	1.08016159	0.923695054	4.130817462	Oncofetal driver
CDC7	2.044943243	0.554973567	1.174069858	4.905169909	Oncofetal driver
PVRL2	38.731626	10.43389142	12.96731692	41.76334649	Oncofetal driver
NIPA1	4.742389681	2.0117874	2.42178205	4.600976201	Oncofetal driver
FEM1B	19.92505658	7.260933834	8.141943358	19.64629815	Oncofetal driver
PTK2	12.8679983	3.735690837	4.061834877	8.821658727	Oncofetal driver
WDR54	2.003632004	0.352786906	0.600420682	2.198361885	Oncofetal driver
DOCK9	5.211171242	2.020032768	2.249513135	4.783636382	Oncofetal driver
TFRC	35.38965403	2.482853454	4.4743869	15.90944623	Oncofetal driver
CHEK2	2.125357737	0.571288808	1.121822582	4.917594801	Oncofetal driver
GLTSCR1	2.774143862	0.710413779	0.67912046	3.310018068	Oncofetal driver
INPP5F	49.20386058	0.835769756	1.29170259	2.214322332	Oncofetal driver
WDR83	3.147793028	1.404741082	1.414378905	3.451026384	Oncofetal driver
HSPH1	28.65075834	10.49660602	13.44676823	28.40800729	Oncofetal driver
C2orf29	15.06209854	5.402797317	6.440706465	15.27524904	Oncofetal driver
OCRL	12.74011484	4.9669809	4.827777748	14.65453654	Oncofetal driver
CUL4B	19.71556615	8.167964227	9.278114826	21.88471147	Oncofetal driver
INCENP	3.383354873	0.860845313	1.393270027	9.479146057	Oncofetal driver
EPCAM	322.2361436	2.354218298	11.65784174	47.11229349	Oncofetal driver
RAB22A	7.983006733	2.949525421	3.952394395	7.113663929	Oncofetal driver
RFC4	4.192668696	1.573371033	1.699608342	9.87715798	Oncofetal driver
SLC38A1	8.918371641	0.554304355	1.463083786	11.24041404	Oncofetal driver
GOLGA5	42.15785039	17.34163253	22.45062107	42.45437476	Oncofetal driver
PTK7	12.1540599	0.143755303	0.160359187	4.056421833	Oncofetal driver
RRAS2	16.09823875	7.667518125	8.125136848	18.49614593	Oncofetal driver
PDCL	8.415651766	1.596524157	2.268488862	8.339887035	Oncofetal driver
RPL35	708.9569516	216.1476101	203.678658	475.8759955	Oncofetal driver
FOXK1	2.950756832	1.123441617	1.097949445	2.924045115	Oncofetal driver
SH3RF2	6.453483132	2.072790323	1.307282155	13.14271195	Oncofetal driver
IGF2BP3	7.965935728	0.060484184	0.045339954	3.514873631	Oncofetal driver
MEIS2	5.582998723	2.265993118	1.226378871	6.327600644	Oncofetal driver
SORT1	15.67447148	3.612868645	2.506512219	9.205334514	Oncofetal driver
PLCE1	13.64123544	0.37920816	0.185292677	5.007846791	Oncofetal driver
CEP89	5.542376734	2.311665066	1.884191476	9.445411578	Oncofetal driver
GPR37	4.25077502	1.792616683	1.692492757	7.828583009	Oncofetal driver
CDCA4	2.5550321	0.37691876	0.414603478	2.902578743	Oncofetal driver
ITPKC	7.326530326	1.186902444	1.92773074	3.902194502	Oncofetal driver

RPS9	65.91652332	32.04337349	27.49156526	62.81423117	Oncofetal driver
MDK	43.12346488	1.167152994	2.365122137	46.18279654	Oncofetal driver
MELK	3.056564176	0.046646812	0.41211377	6.965872026	Oncofetal driver

Abbreviations: RPKM, Reads Per Kilobase per Million mapped reads.

Supplementary Table 2. Summary of the different AS events regulated by ESRP2 in 97H and Huh7 cells

AS type	Total events		Shared events
	97H	Huh7	
SE	1640	1765	581
A3SS	234	165	34
A5SS	165	173	30
MXE	338	277	55
RI	219	186	36
Sum	2596	2566	736

Abbreviations: AS: alternative splicing; SE: skipped exon; A3SS: alternative 3' splice site; A5SS: alternative 5' splice site; MXE: mutually exclusive exon; RI: retained intron; Sum: summary.

Supplemental Table 3. ESRP2-affected SE events shared by 97H and Huh7 cell lines with FDR value less than 0.05

Gene	Chromosome	Exon start	Exon end	IncLevel		FDR	IncLevel		FDR
				97H_ESRP2	97H_Vec		Huh7_ESRP2	Huh7_Vec	
ACSL3	2	222900673	222900780	1	0.471	0	0.651	0.435	0.001063479
ANAPC11	17	81893551	81893614	1	0.787	0	1	0.735	3.18E-12
ARFGAP2	11	47172709	47172751	0.551	0.017	0	0.178	0	0.003939306

ARHGEF11	1	156938417	156938513	0.509	0.958	0	0	0.963	0
BAIAP2	17	81110913	81110959	0.744	0.072	0	0.721	0	3.07E-05
CD44	11	35211245	35211449	0.701	0.038	0	1	0.146	8.01E-10
CEACAM1	19	42511575	42511628	0.251	0.977	0	0.033	0.579	0
CTNND1	11	57791384	57791673	0.115	0.406	0	0.055	0.452	0
ENAH	1	225504990	225505053	0.564	0.08	0	0.83	0.09	0
EXOC7	17	76091142	76091235	0.239	1	0	0.02	0.589	0
FN1	2	215380810	215381080	0.402	0.496	0	0.225	0	0.006029803
INF2	14	104715283	104715340	0.961	0.399	0	0.985	0.353	0
RAC1	7	6398661	6398718	0.143	0.013	0	0.245	0.001	0
SLK	10	104010815	104010908	0.474	0.012	0	0.805	0.1	0
USO1	4	75795335	75795356	0.642	0	0	0.983	0	0
SCRIB	8	143807551	143807614	0.46	0.891	6.90E-12	0.19	0.973	0
DGUOK	2	73957124	73957240	0.463	1	1.52E-10	0.613	1	2.15E-10
RALGPS2	1	178892229	178892307	0.681	0.132	3.50E-10	0.909	0.211	2.73E-11
SPTAN1	9	128592982	128593042	0.913	0.584	1.42E-09	0.96	0.331	0
EIF4G1	3	184315762	184315856	0.098	1	3.48E-09	0.713	0.397	1.45E-13
FGFR3	4	1803691	1803836	0.112	0.842	8.75E-09	0.695	0.597	8.67E-06
MAP3K7	6	90544551	90544632	0.739	0.282	3.29E-08	0.827	0.313	7.42E-14
NF2	22	29683019	29683064	0.96	0.381	5.24E-08	0.76	0.274	2.17E-07
YAP1	11	102209516	102209564	1	0.474	3.73E-07	1	0.532	0.001460476
EPN1	19	55677549	55677734	0.44	0.012	1.08E-05	0.596	0.273	1.28E-06
METTL23	17	76729689	76729794	0.952	0.558	1.08E-05	0.742	0.26	0.001808785
EPB41	1	29096154	29096604	0.531	0.09	1.49E-05	0.305	0.075	0.038863203
AURKA	20	56390555	56390665	1	0.662	0.000118066	1	0.543	4.99E-08
MYOF	10	93392916	93392955	0.758	0.963	0.000131866	0.312	0.966	4.45E-06
LRRC14	8	144518748	144518815	1	0	0.000206041	1	0	0.03936474
CYP4F3	19	15643906	15644051	0.673	0.126	0.000323865	0.944	0.101	2.26E-12

ZDHHC16	10	97453798	97453846	1	0.656	0.000456597	1	0.431	2.40E-05
CLK4	5	178617343	178617434	1	0.157	0.000604885	1	0.383	0.007020293
KIF23	15	69425281	69425323	0.447	0.186	0.000643692	0.647	0.2	7.43E-10
SLMAP	3	57925844	57925934	0.547	0.846	0.001197572	0.139	1	6.09E-10
METTL26	16	634888	635063	0.732	0.467	0.001571588	0.737	0.34	0.005324772
MYO1B	2	191402631	191402718	0.214	0.525	0.001978789	0.045	0.486	0
UAP1	1	162592731	162592782	0.687	0.251	0.002095852	0.871	0.054	0
STAG3L1	7	75365951	75366059	1	0.646	0.002225222	1	0.225	0.002098887
GNAS	20	58898940	58898985	0.389	0.547	0.00223901	0.403	0.813	2.01E-05
AC135983.2	15	32524470	32524600	0.925	0.348	0.002879224	0.923	0.438	0.025747776
GOLIM4	3	168040785	168040869	0.669	0.886	0.005615664	0.403	0.813	2.01E-05
UCHL1	4	41257114	41257126	0	0.946	0.006175407	0.969	0.639	0.00424439
GALNT11	7	152027596	152027718	0	0.186	0.007753774	0.462	0.565	0.023617591
SCAMP3	1	155261656	155261734	0.738	0.915	0.008169679	0.647	0.858	0.046618279
ZNF664	12	124003422	124003540	1	0.482	0.011785251	1	0.041	0.000430979
EEF1AKMT3	12	57774628	57774767	1	0	0.012459183	0.094	1	0.038809154
CCDC50	3	191375061	191375589	0.103	0.005	0.012855359	0.389	0.018	0
ESYT2	7	158752780	158752843	0.482	0.314	0.013232155	0	1	1.37E-08
STYXL1	7	76000889	76001002	1	0.8	0.024790239	0.617	1	2.42E-08

Abbreviations: SE, skipped exon; FDR, false discovery rate; lncLevel, exon inclusion level. Likelihood-ratio test was used for statistical analysis.

Supplementary Table 4. List of ESRP2-regulated AS targets crucial for HCC progression.

AS tagets	HR	95% CI	P.value[1]	P.value[2] (log-rank test)	P.value (T. vs. N)[3] (Student's t test)
BAIAP2	0.39	(0.18-0.86)	0.02	0.006808707	6.11E-17
CCDC50	0.27	(0.11-0.71)	0.0078	0.000567168	1.05E-09
CEACAM1	9.3	(3-29)	0.00014	1.25E-05	0.031818723
CYP4F3	0.004	(0.00043-0.037)	0.0000011	1.09E-05	9.94E-29
EPN1	0.13	(0.023-0.77)	0.024	0.000122828	5.47E-08
FN1	3.3	(1.5-7.3)	0.0023	0.000220509	4.80E-82
MAP3K7	0.31	(0.12-0.81)	0.016	0.001135039	7.05E-06
NF2	0.32	(0.13-0.76)	0.01	0.005561328	3.25E-22
RALGPS2	0.2	(0.055-0.74)	0.016	0.031076459	1.35E-10
SCAMP3	0.012	(0.00061-0.23)	0.0032	0.003541004	2.75E-12
SCRIB	0.22	(0.073-0.69)	0.0093	0.002531667	1.03E-14
SPTAN1	0.27	(0.08-0.95)	0.041	0.000682317	3.20E-10
UAP1	0.41	(0.21-0.81)	0.01	0.000191772	4.35E-32
USO1	0.41	(0.21-0.82)	0.011	2.87E-05	1.75E-61
ZDHHC16	22	(6.7-71)	3.30E-07	1.08E-06	0.031961108

[1], Cox's proportional hazards regression model was used to predict the significant prognostic factor for OS.

[2], Kaplan-Meier survival analysis was performed and log-rank test was used for statistical analysis.

[3], The PSI index of each AS target was analyzed in TCGA-HCC cohort. Student's t test was used to campare the PSI difference between tumor and non-tumor tissues.

Abbreviations: AS, alternative splicing; HR, hazard ratio; CI, confidence interval; PSI, percent spliced in.

Supplementary Table 5. The predicted binding free energy results for TAB3-TAK1_FL/ΔE12 heterodimers. The energy of each complex was calculated by the PRODIGY web server.

Binding energy	TAB3-TAK1_FL complex	TAB3-TAK1_ΔE12 complex
complex1_ΔG (kcal mol^{-1})	-11.1	-10.3
complex2_ΔG (kcal mol^{-1})	-11	-16
complex3_ΔG (kcal mol^{-1})	-9.5	-12.1
Average_ΔG (kcal mol^{-1})	-10.5333	-12.8

Supplementary Table 6. The log data from PRODIGY records the number of contact points among interacting residues and the spectrum of molecular forces.

Parameters	TAB3-TAK1_FL.complex1	TAB3-TAK1_ΔE12.complex2
No. of intermolecular contacts	63	134
No. of charged-charged contacts	9	13
No. of charged-polar contacts	5	17
No. of charged-apolar contacts	16	35
No. of polar-polar contacts	2	11
No. of apolar-polar contacts	15	34
No. of apolar-apolar contacts	16	24
Percentage of apolar NIS residues	39.4	38.93
Percentage of charged NIS residues	21.39	21.37
Predicted binding affinity (kcal.mol^{-1})	-11.1	-16
Predicted dissociation constant (M) at 25.0˚C	7.30E-09	1.80E-12

Supplementary Table 7. Sequences of primers used for qPCR, BGS, and RT-PCR

	Primer	Sequences (5'-3')
qPCR	ESRP2_qF	TTGCAGCAAGGCTGATGTG
	ESRP2_qR	GTTGAGGCAGAGTGCTACACC
	18S_qF	CTCTTAGCTGAGTGTCCCGC
	18S_qR	CTGATCGTCTTCGAACCTCC
BGS	ESRP2_BGS_F	GGATAAGGTGTTGTAGTAGGTGAG
	ESRP2_BGS_R	ATAATCCCCCAAACCAAAC
RT-PCR	TAK1_E12_F	GAGGGCAAGAGGATGAGTGCTGACA
	TAK1_E12_R	CCTGACCAGGTTCTGTTCCAGTTAC

Abbreviations: qPCR, quantitative real-time PCR; BGS, bisulfite genomic sequencing; RT-PCR, reverse-transcription PCR
